# Tosylhydrazine-promoted self-conjugate reduction–Michael/aldol reaction of 3-phenacylideneoxindoles towards dispirocyclopentanebisoxindole derivatives

**DOI:** 10.3762/bjoc.18.49

**Published:** 2022-04-27

**Authors:** Sayan Pramanik, Chhanda Mukhopadhyay

**Affiliations:** 1 Department of Chemistry, University of Calcutta, 92 APC Road, Kolkata-700009, India

**Keywords:** chemoselective conjugate reduction, dispirocyclopentanebisoxindole scaffolds, metal-free, one-pot operation, reductive cyclization

## Abstract

An efficient tosylhydrazine-mediated conjugate reduction of 3-phenacylideneoxindole and sequential Michael/intramolecular aldol reaction is reported under base-catalyzed conditions towards the formation of densely substituted dispirocyclopentanebisoxindole derivatives. The reaction proceeded in a diastereoselective manner to afford four chiral stereocenters. The method also has advantages of wide substrate scope, readily available starting materials and operational simplicity through one pot reaction.

## Introduction

**Figure 1:**
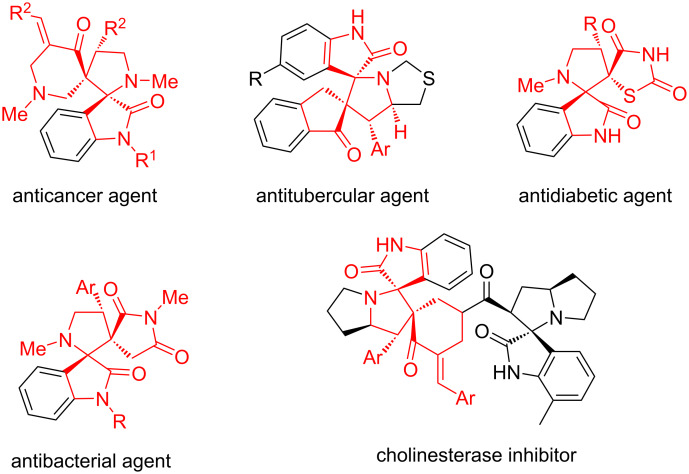
Representative bioactive dispirooxindoles.

There is a vast demand of the structurally complex spirooxindole scaffold which is an important structural motif that is embodied in a number of bioactive natural products and medicinally relevant compounds [1–2]. Among various spirooxindole motifs, bispirooxindoles fusing two spirooxindole cores exhibit a wide range of important biological activities, for example, anticancer, antitubercular, antidiabetic, antibacterial as well as cholinesterase inhibition (Figure 1) [3–7]. These unique chemical and biological characteristics of bispirooxindoles have stimulated the concentration of the synthetic community on developing new synthetic strategies towards their synthesis [8–14].

Among the known synthetic methodologies, the domino cycloaddition reaction of benzimidazolium salts with two molecules of 2-(2-oxoindolin-3-ylidene)acetates provided as an important tool for the construction of dispirooxindoles [15]. In particular, most of the dispirooxindole derivatives were synthesized on the basis of Michael acceptor ability of 3-alkylidene oxindoles with various donor synthons [16–22]. However, in spite of the progress in the synthesis of dispirocyclopentanebisoxindoles with concise structural and stereochemical diversity, the one-pot operation without chiral catalyst is still an atom-economical and operationally simple procedure to demonstrate the construction of dispirooxindoles. In the recent years, the diastereoselective construction of dispirocyclopentanebisoxindoles consisting of two spirooxindole motifs has acquired attention from several research groups. In 2013, Yan et al. reported the construction of dispirocyclopentanebisoxindoles from the base-catalyzed domino reaction between two molecules of 3-phenacylideneoxindoles by participation of different solvents [23]. In 2016, the same research group reported the synthesis of dispirocyclopentanebisoxindoles from two molecules of 3-phenacylideneoxindoles, accomplished by reduction of one molecule of 3-phenacylideneoxindole by thiol [24]. In 2017, Thennarasu et al. [25] reported the tandem oxidation/Michel-aldol reaction of 3-phenacyloxindoles for the synthesis of dispirocycliopentanebisoxindoles. Again, in 2018, Yang et al. [26] synthesized functionalized dispiro[indoline-3,1-cyclopentane-3,3-indolines] via cyclodimerization of 3-phenacylideneoxindolines with benzoyl hydrazides and arylhydrazines. Encouraged by these synthetic methodologies and in continuation to our efforts towards the generation of new cascade reactions to construct carbo- and heterocyclic moieties [27–28], we aimed to synthesize dispirocyclopentanebisoxindoles in one pot operation using a new protocol.

**Scheme 1:**
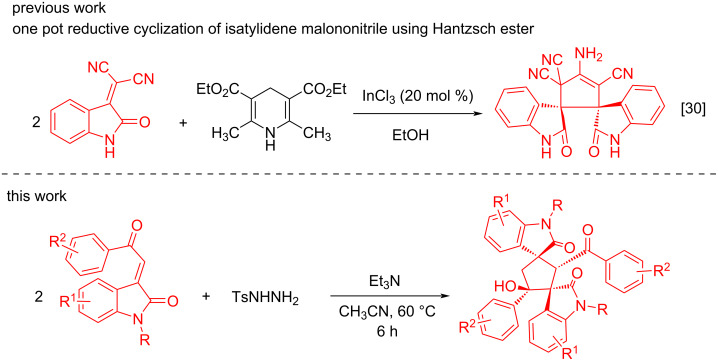
Reductive cyclization for the synthesis of dispirocyclopentanebisoxindole derivatives.

Chemical hydrogenation of double bonded compounds like α,β-unsaturated ketones, approaching to saturated ketones, is an important functional group transformation for the synthesis of heterocyclic and carbocyclic building blocks and reactive intermediates. Besides the use of various reducing agents, it is observed that tosylhydrazine develops the transition-metal-free and highly chemoselective conjugate reduction of α,β-unsaturated ketones [29]. Using this method, we developed the in situ reduction of 3-phenacylideneoxindoles by tosylhydrazine and sequential Micheal addition/cyclization with another molecule of 3-phenacylideneoxindole towards bisspirocyclopentenebisoxindole derivatives. Although Shanthi et al. [30] reported the In(III)-catalyzed reductive cyclization of isatylidene malononitriles using the Hantzsch ester as reducing agent for the synthesis of dispirocyclopentanebisoxindole. Our work utilizes tosylhydrazine as chemoselective reducing agent for 3-phenacylideneoxindoles and thereafter base-catalyzed dimerization produces dispirocyclopentanebisoxindoles in a transition-metal-free protocol (Scheme 1).

## Results and Discussion

There is extensive application of 3-phenacylideneoxindoles generating diverse reaction strategies following the regioselective and diastereoselective synthesis of carbocyclic and heterocyclic frameworks [31–32]. During our preliminary investigations as shown in Table 1, we commenced our study by reacting two equivalents of (*E*)-3-(2-(4-chlorophenyl)-2-oxoethylidene)indolin-2-one (**1a**) under reflux conditions with one equivalent of tosylhydrazine (**2**) as hydrogen source in ethanolic solution in the presence of K_2_CO_3_ as base. After the reaction was completed, we observed that the reductive cyclo-dimerization occurs smoothly to give the corresponding dispirocyclopentanebisoxindole **3a** in 68% isolated yield with good diastereoselectivity (>95:5 dr) (Table 1). NMR and HRMS analyses confirm the structure of the product **3a**. With 3-phenacylidene oxindole **1a** and tosylhydrazine (**2**) as model substrates, we started the optimization of the reaction upon various parameters such as temperature, base and solvent. A variety of bases were examined in the reaction, both inorganic and organic bases were well acted for this reductive cyclization, among the bases screening, Et_3_N was the most effective when employed with a particular solvent (Table 1). In order to improve the yield of the reaction we optimized the reaction temperature and it was found that the reaction at 60 °C gave a satisfying yield of 82% with reduced reaction time (Table 1).

**Table 1:** Optimization study for the formation of **3a**.^a^

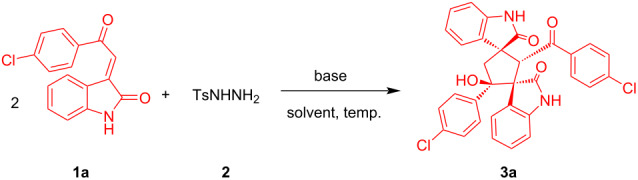					

entry	solvent	base [equivalents]	temp [°C]/time	yield^b^	dr

1	EtOH	K_2_CO_3_ (0.5)	80, 9 h	68	>95:5
2	CH_3_CN	Et_3_N (0.5)	rt, 12 h	trace	n.d.
**3**	**CH** **_3_** **CN**	**Et** **_3_** **N (0.5)**	**60, 6 h**	**82**	**>95:5**
4	CH_3_CN	Et_3_N (0.5)	80, 6 h	72	>95:5
5	CH_3_CN	DBU (1)	60, 6 h	45	n.d.
6	CH_3_CN	Cs_2_CO_3_ (0.5)	60, 8 h	63	>95:5
7	CH_3_CN	NaOH (0.5)	60, 8 h	35	n.d.
8	EtOH	Na_2_CO_3_ (0.5)	80, 9 h	41	n.d.
9	CH_3_CN	Et_3_N (1)	60, 12 h	55	>95:5
10	CH_3_CN	Et_3_N (1)	60, 6 h	60	>95:5
11	dioxane	K_2_CO_3_ (0.5)	80, 9 h	40	n.d.
12	toluene	Et_3_N (0.5)	100, 10 h	–	n.d.
13	CH_3_CN	DABCO (0.5)	60, 8 h	62	>95:5
14	MeOH	K_2_CO_3_ (0.5)	80, 9 h	49	n.d.
15	MeOH	Et_3_N (1)	60, 9 h	54	n.d.
16	DCM	Et_3_N (0.5)	rt, 6 h	–	n.d.

^a^Reaction conditions: substrate **1a** (1 mmol), **2** (0.5 mmol), different catalysts, different solvents, different temperatures, different times. ^b^Isolated yields.

**Scheme 2:**
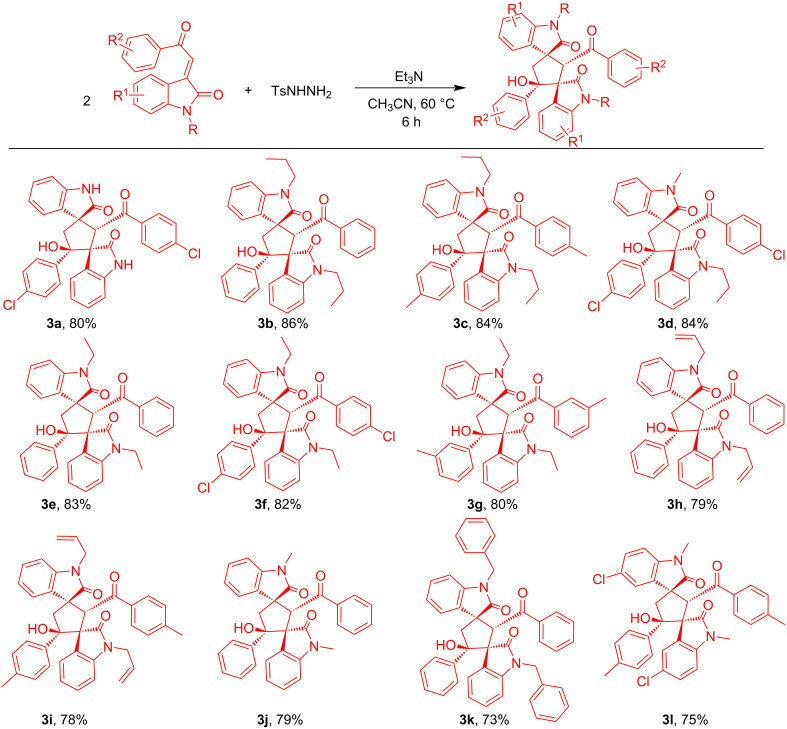
Substrate scope of product **3** (part 1). Reaction conditions: substrates: **1** (1 mmol) and **2** (0.5 mmol), were stirred with Et_3_N (1 equiv) at 60 °C for 6 h in 6 mL CH_3_CN.

**Scheme 3:**
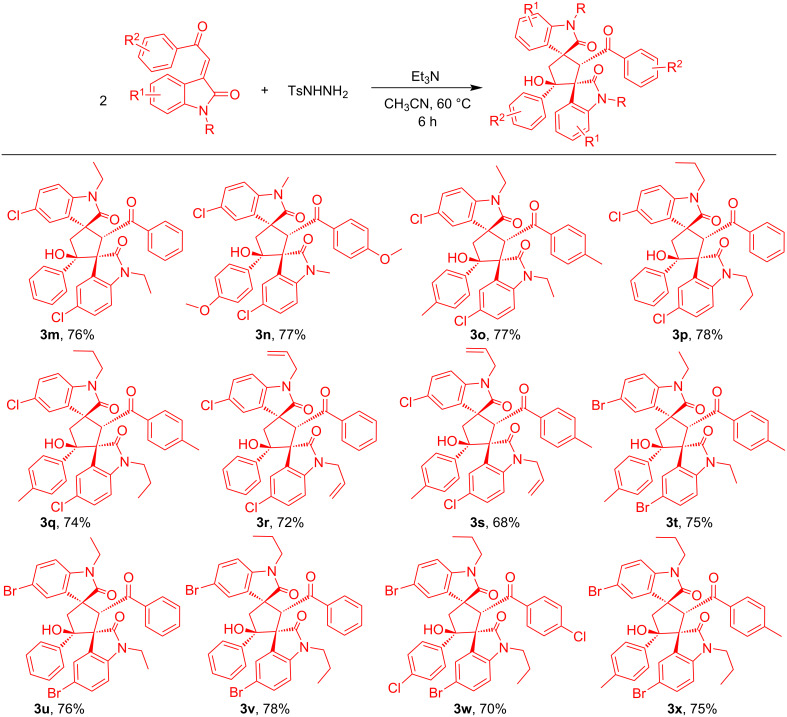
Substrate scope of product **3** (part 2). Reaction conditions: substrates: **1** (1 mmol) and **2** (0.5 mmol), were stirred with Et_3_N (1 equiv) at 60 °C for 6 h in 6 mL CH_3_CN.

Solvents also played an important role and the effect of solvents on this reductive cyclization was also examined. A moderate yield was obtained when MeOH, EtOH or dioxane were employed as solvent. Unfortunately, when the reaction was conducted in a less polar solvent like toluene or DCM the desired product was not obtained. After investigatiing different solvents, it was revealed that CH_3_CN is the perfect solvent of choice (Table 1). Together, this screening resulted in the optimal conditions, which are: **1a** (1 mmol), **2** (0.5 mmol) and Et_3_N (0.5 mmol) in CH_3_CN medium at 60 °C (Table 1, entry 3). With these optimized conditions in hand, we explored the scope of this transformation. At first we investigated the substrate scope of 3-phenacylideneoxindoles varying substitution patterns and it was observed that 3-phenacylideneoxindoles with various substitutions on the indoline ring could be unambiguously converted into the corresponding products (Scheme 2, Scheme 3). Halo-substituted indolines (5-Cl, 5-Br) were found to furnish the desired products in good yields (70–80%) which provided possibilities of further functionalization. In addition, the electronic nature of the substituent on the aryl ring of **1** was examined and believed to have subtle influence on the yield of the product. The aryl ring with an electron-withdrawing group at the *para* position (**3a, 3d, 3f** and **3w**) and electron-donating group at the *para* and *meta* position (**3c, 3g, 3i, 3l, 3n, 3o, 3q, 3s, 3t and 3x**) gave a good yield of the corresponding product, respectively. Here also, the halo-substituted aryl ring on **1** can be further used for transition-metal-catalyzed cross coupling reactions, thus providing a very good application. Then the effect of N-protecting groups on the indoline ring was also investigated. Varieties of N-protected (Me, Et, propyl, benzyl and allyl) 3-phenacylideneoxindoles were used and provide good to excellent yield of the dimerized products (Scheme 2, Scheme 3). During the detailed study of the reaction, we conducted the reaction with 2-(2-oxindole-3-ylidene)acetate, but unlike 3-phenacylideneoxindole this substrate does not take part in the reductive cyclization.

**Figure 2:**
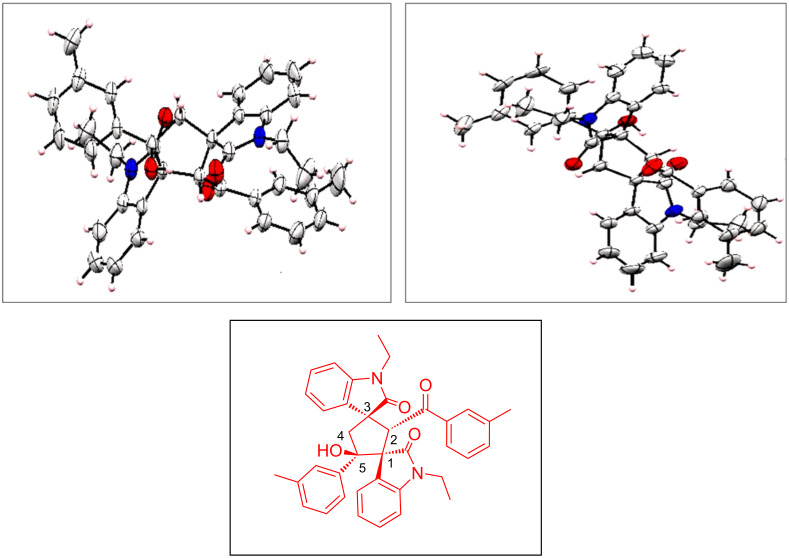
ORTEP diagram of product **3g** (CCDC NO. 2072521).

In the prepared dispirocyclopentylbisoxindoles, there are four diastereomeric centers in the newly formed cyclopentane ring, so there is a possibility for existence of many diastereomers for each product, but the single crystal X-ray diffraction analysis confirmed the diastereoselectivity of **3**. A single crystal of **3g** was successfully obtained by slow evaporation of the solvent with the structure unequivocally confirmed by X-ray diffraction analysis (Figure 2). The single crystal X-ray diffraction analysis also revealed the presence of four chiral stereocenters with two oxindole moieties at 1,3-possition that are in *trans* orientation to the 2-benzoyl group and the 5-aryl group is in *cis* orientation (Figure 2). This observation proved that the most thermodynamically stable diastereomer was formed by multistep reaction progression where, to overcome severe steric hindrance, two oxindole moieties are in *trans* orientation. HPLC data of compound **3o** ensures >95:5 dr (Supporting Information File 1).

**Figure 3:**
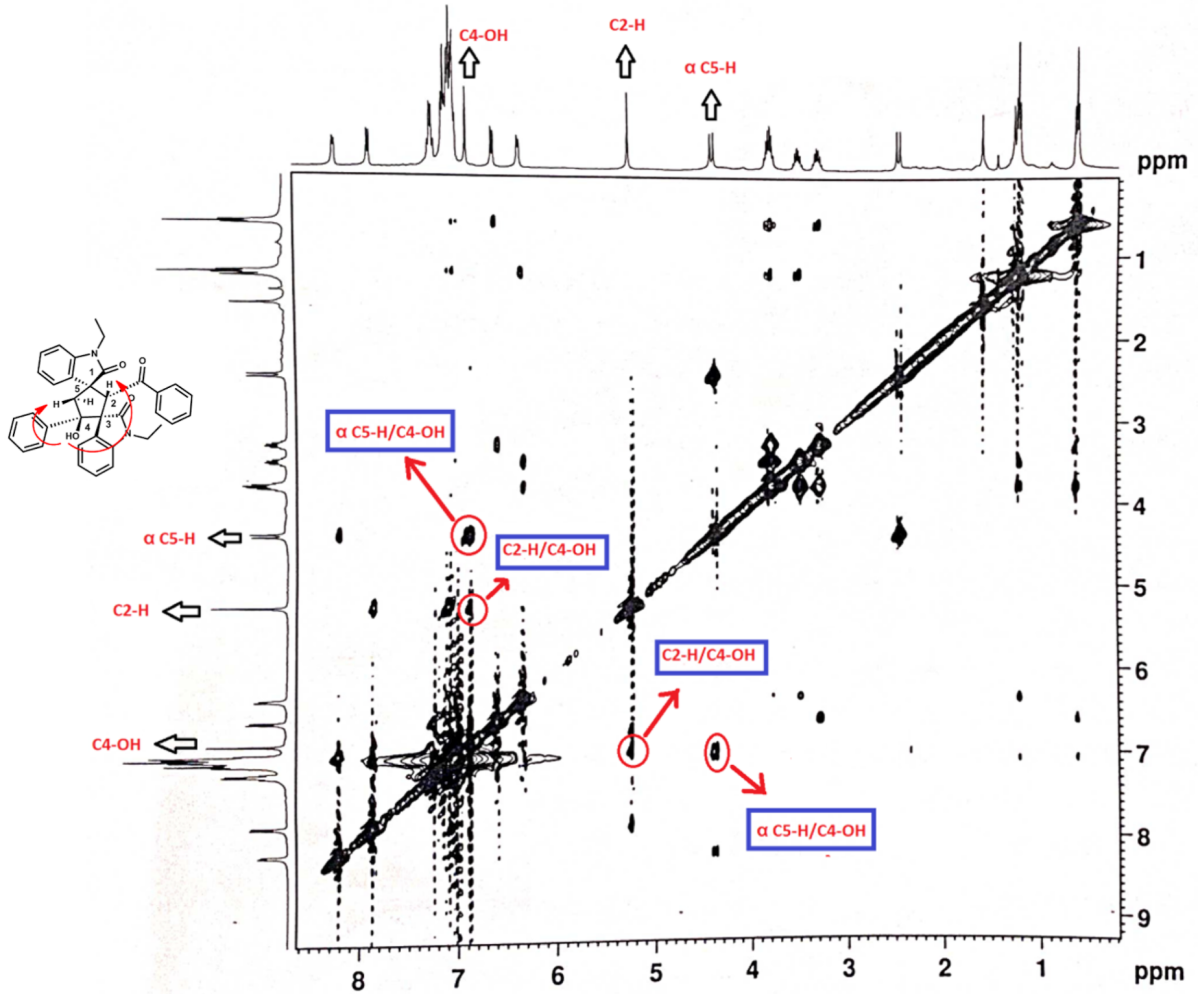
NOESY Spectra of compound **3e**.

The structure was further confirmed by NOESY spectra of compounds **3e** (Figure 3) and **3j** (Supporting Information File 1). The correlation between two *syn*-Hs of C4-OH/C2-H and another two *syn*-Hs of C4-OH/α C5-H were well visualized in the NOESY spectra.

**Scheme 4:**
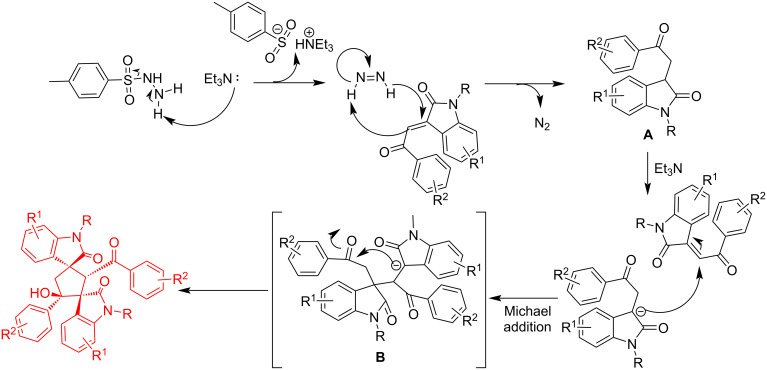
Plausible mechanism of the reaction.

In order to explain the formation of dispirocyclopentanebisoxindole after studying some related literatures [23–25,29] a plausible reaction mechanism is shown in Scheme 4. At first, tosylhydrazine as hydride source [29] promotes reduction of 3-phenacylideneoxindole towards 3-phenacylindolinone **A**. Then, under basic conditions, the carbanion of 3-phenacylindolinone, which acts as Michael donor, is formed. After this the carbanion undergoes Michael addition with another molecule of 3-phenacylidieneoxindole to yield intermediate **B**. Finally, an intramolecular aldol condensation takes place between the carbanion and the carbonyl group of intermediate **B** towards the formation of the dispirocyclopentanebisoxindole derivative.

**Figure 4:**
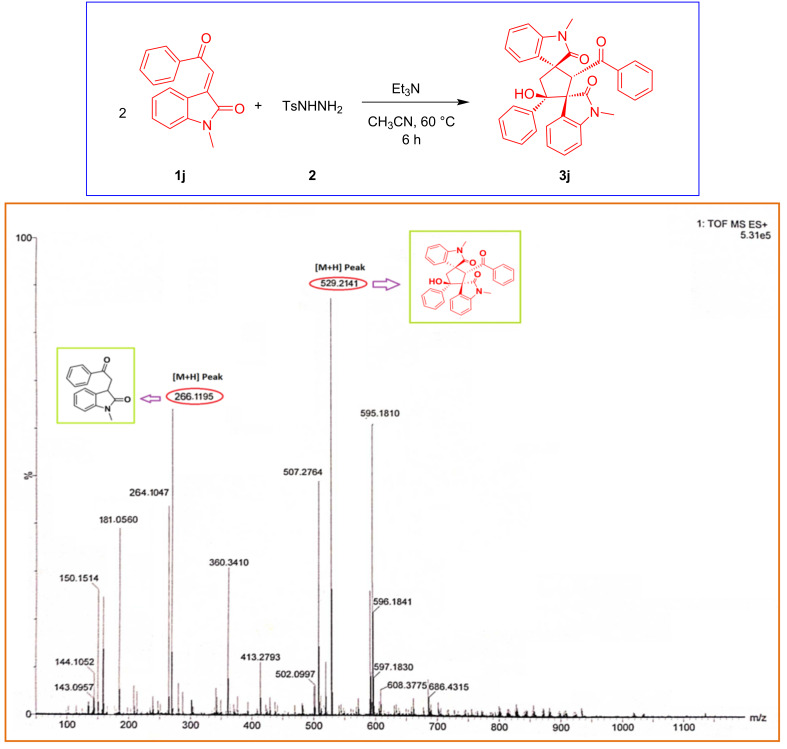
HRMS spectrum of the crude reaction mixture after 1 hour of the reaction.

To support the proposed mechanism at first we performed an HRMS analysis of the crude reaction mixture before completion of the reaction, starting with (*E*)-1-methyl-3-(2-oxo-2-phenylethylidene)indolin-2-one (**1j**) and tosylhydrazine under the optimized reaction conditions. To our delight, the HRMS spectrum clearly shows the peaks of the corresponding intermediate **A** at 266.1195 [M + H] and final product **3j** at 529.2141 [M + H] (Figure 4).

**Scheme 5:**
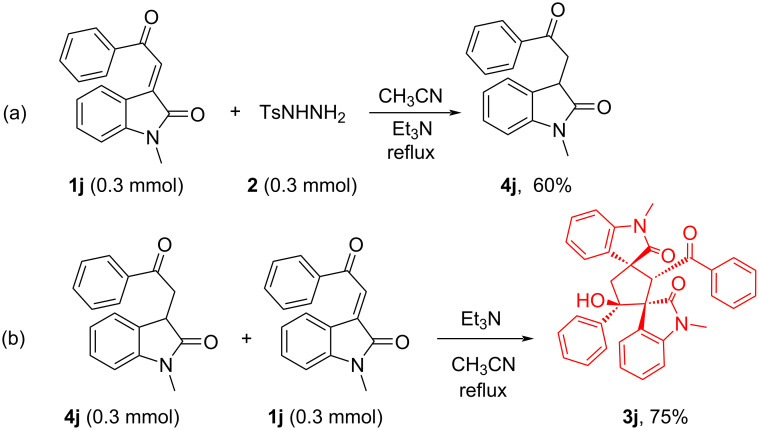
Control experiment.

Further we attempted a study to isolate the corresponding intermediate **A**. We carried out a reaction by taking an 1:1 equivalent mixture of tosylhydrazine and (*E*)-1-methyl-3-(2-oxo-2-phenylethylidene)indolin-2-one (**1j**) in acetonitrile with Et_3_N catalyst under refluxing conditions, and isolated the corresponding saturated ketone 1-methyl-3-(2-oxo-2-phenylethyl)indolin-2-one (**4j**) in 60% yield, which results from reduction of the double bond of α,β-unsaturated ketone (Scheme 5). The structure of compound **4j** was determined by NMR spectral data. After that we conducted another reaction between saturated ketone **4j** with unsaturated ketone (*E*)-1-methyl-3-(2-oxo-2-phenylethylidene)indolin-2-one (**1j**) under base-catalyzed conditions, gratifyingly we obtained our desired product dispirocyclopentanebisoxindole **3j**. Therefore, we can conclude that 3-phenacylindolinone is in situ generated in the reaction medium from reduction of 3-phenacylidieneoxindole by tosylhydrazine, which undergoes cyclodimerization with another equivalent of 3-phenacylidieneoxindole under base-catalyzed conditions. This study also proves that two equivalents of 3-phenacylideneoxindole are required for reductive cyclodimerization.

## Conclusion

In conclusion, we have successfully developed a simple and efficient methodology towards the one-pot synthesis of dispirocyclopentanebisoxindole derivatives through base-catalyzed reductive cyclization of two molecules of 3-phenacylideneoxindoles. In this reaction tosylhydrazine serves as reducing agent to reduce one molecule of 3-phenacylideneoxindole to 3-phenacylindolinone which, under base-catalyzed conditions, involves a sequential Michael addition/intramolecular aldol condensation with another molecule of 3-phenacylideneoxindole to give dispirocyclopentanebisoxindoles. The notable advantage of this method is a metal-free reducing agent, mild reaction conditions, readily available starting materials and variety of substrates. Further investigation towards the application of this methodology and structural diversifications of this moiety are underway in our laboratory.

## Experimental

**General method:** All commercially available chemicals were purchased from Aldrich, USA or Spectrochem, India, and used without further purification. All solvents were used as received. The progress of the reaction was checked by TLC glass sheets pre-coated with silica gel (with binder, 300 mesh, Spectrochem) and column chromatography was performed using silica gel (100–200 mesh). Bruker 300 MHz and 400 MHz instruments were used for ^1^H and ^13^C NMR spectra at 300 MHz, 400 MHz and 75 MHz, 100 MHz respectively. Chemical shifts are reported in parts per million (ppm) downfield from an internal TMS (tetramethylsilane) reference. Coupling constants (*J*) are reported in hertz (Hz), and spin multiplicities are represented by the symbols s (singlet), brs (broad singlet), d (doublet), t (triplet), q (quartet) and m (multiplet). HRMS with an ESI resource were acquired using a Waters XEVO-G2S Q TOF mass spectrometer. HPLC were recorded using an Agilent 1200 Series auto sampler HPLC system. Melting points were recorded with an open capillary on an electrical melting point apparatus and the single crystal structure of the synthesized compound was confirmed by an X-ray crystallography experiment on a Bruker SMART diffractometer.

**General procedure for the synthesis of compound 3:** In a manner similar to [28], 3-phenacylideneoxindole (1 mmol), tosylhydrazine (0.5 mmol) and Et_3_N (1 equivalent) were added with 6 mL CH_3_CN in a dry 10 mL round bottomed flask provided with a reflux condenser. Then the reaction mixture was stirred at 60–70 °C for 6 hours. After reaching the completion, the reaction mixture was examined by TLC. Then the reaction mixture was cooled to room temperature, diluted with 10 mL of water and extracted with EtOAc (3 × 10 mL). The organic layers were combined and washed with brine, then dried over anhydrous Na_2_SO_4_. After the solvent was removed under reduced pressure, the crude product was purified by column chromatography using 100–200 mesh silica gel and petroleum ether–ethyl acetate mixture as the eluent to afford the desired product **3**.

## Supporting Information

File 1 Experimental and analytical data.

File 2 CIF-file of compound **3g**.

File 3 Check-CIF-file for compound **3g**.
