# Supplementary material for: Tosylhydrazine-promoted self-conjugate reduction–Michael/aldol reaction of 3-phenacylideneoxindoles towards dispirocyclopentanebisoxindole derivatives
Source: Beilstein J Org Chem. 2022 Apr 27;18:469–78. doi: 10.3762/bjoc.18.49 (PMC9062653; doi:10.3762/bjoc.18.49)
Supplement: File 1 — Experimental and analytical data. [file Beilstein_J_Org_Chem-18-469-s001.pdf]

BEILSTEIN JOURNAL OF ORGANIC CHEMISTRY

Supporting Information

for

Tosylhydrazine-promoted self-conjugate reduction–Michael/aldol reaction of 3-phenacylideneoxindoles towards dispirocyclopentanebisoxindole derivatives

Sayan Pramanik and Chhanda Mukhopadhyay

Beilstein J. Org. Chem. **2022,** *18,* 469–478. doi:10.3762/bjoc.18.49

Experimental and analytical data

License and Terms: This is a supporting information file under the terms of the Creative Commons Attribution License (https://creativecommons.org/licenses/by/4.0). Please note that the reuse, redistribution and reproduction in particular requires that the author(s) and source are credited and that individual graphics may be subject to special legal provisions.
The license is subject to the *Beilstein Journal of Organic Chemistry* terms and conditions: (https://www.beilstein-journals.org/bjoc/terms)

Contents:

Experimental section:

1. General methods……………………………………………………S2
2. General procedure for the synthesis of compound.....................S2–3
3. Characterisation of compounds ………………………………S4–16
4. ^{1}H and ^{13}C spectra of all compounds......................................S17–47
5. HPLC of **3o**…………………………………………………S48–49
6. ORTEP diagram of product **3g**..S50
7. NOESY Spectra of product **3e** and **3j**………………………S51–52
8. HRMS of crude reaction mixture………………………………..S53
9. References...……………………………………………………..S54

General Information:

All commercially available chemicals were purchased from Aldrich, USA or Spectrochem, India, and used without further purification. All solvents were used as received. The progress of the reaction was checked by glass sheets pre-coated TLC with silica gel (with binder, 300 mesh, Spectrochem) and column chromatography was performed using silica gel (100-200 mesh). Bruker 300 MHz and 400 MHz instruments were used for ^{1}H and ^{13}C NMR spectra at 300 MHz, 400 MHz and 75 MHz, 100 MHz respectively. Chemical shifts are reported in parts per million (ppm) downfield from an internal TMS (tetramethylsilane) reference. Coupling constants (J) are reported in hertz (Hz), and spin multiplicities are represented by the symbols s (singlet), brs (broad singlet), d (doublet), t (triplet), q (quartet) and m (multiplet). HRMS with an ESI resource were acquired using a Waters XEVO-G2S Q TOF mass specrtrometer. 2400 Series II CHNS Analyzer, Perkin Elmer USA was used for elemental analyses. HPLC were recorded using anAgilent 1200 Series auto sampler HPLC system. Melting points were recorded with an open capillary on an electrical melting point apparatus and the single crystal structure of the synthesized compounds were confirmed by an X-ray crystallography experiment on a Bruker SMART diffractometer.

General procedure for the synthesis of compound 1:

In a manner similar to [*1*] a mixture of isatin (A) (1mmol) and acetophenone (B) (1 mmol), was taken in a 10 mL round-bottom flask containing 5 mL ethanol solvent and triethylamine base (0.4 mmol). The reaction mixture was heated to reflux for 6 h maintaining anhydrous conditions. After complete conversion of the starting materials (monitored by TLC), ethanol was distilled out under reduced pressure and the crude product was purified by column chromatography using 100–200 mesh silica gel and petroleum ether–ethyl acetate mixture as the eluent to afford 3-hydroxy-3-(2-oxo-2-phenylethyl)indolin-2-one (C) . Then the product C was acidified with an AcOH and HCl mixture (4:1) and heated at 80 °C for 1 hour, after this the reaction was diluted with 50 mL cold water and extracted with EtOAc (3 × 10 mL). The organic layer was combined and washed with brine and dried over anhydrous Na_2SO_4. After this the crude product was purified by column chromatography using 100–200 mesh silica gel and petroleum ether–ethyl acetate mixture as the eluent to afford the desired product **1**.

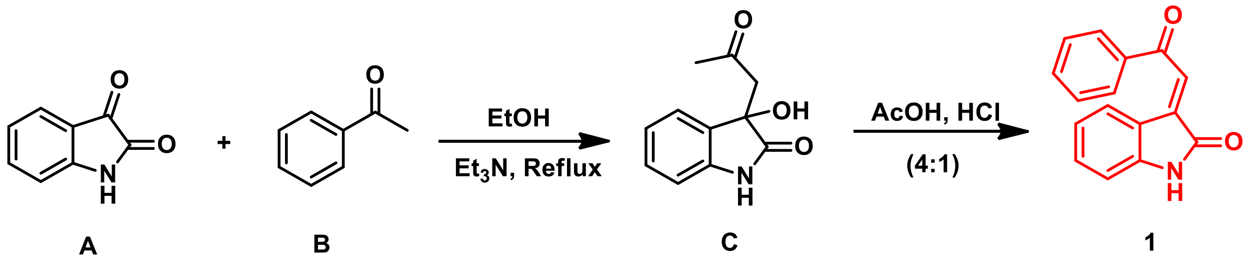

Procedure for the synthesis of compound 3: In a manner similar to [1] 3-phenacylideneoxindole (1 mmol), Tosyl hydrazine (0.5 mmol) and Et_3N (0.5 mmol) were added with 6 mL CH_3CN in a dry 10 mL round bottomed flask provided with a reflux condenser. Then the reaction mixture was stirred at 60–70 °C for 6 hours. After reaching the completion, the reaction mixture was examined by TLC. Then the reaction mixture was cooled to room temperature and diluted with 10 mL of water and extracted with EtOAc (3 × 10 mL). The organic layer was combined and washed with brine and dried over anhydrous Na_2SO_4. After the solvent was removed under reduced pressure, the crude product was purified by column chromatography using 100–200 mesh silica gel and petroleum ether–ethyl acetate mixture as the eluent to afford the desired product **3**.

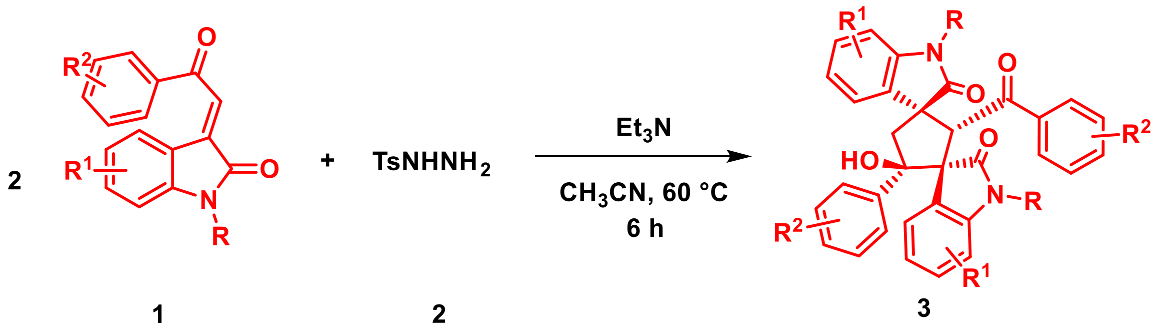

Characterization of Compounds

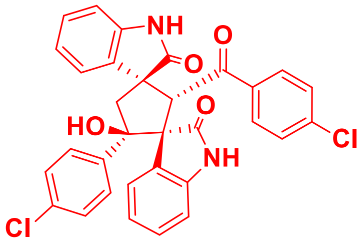

3a: White solid, Yield: 455.3 mg, 80% ; R_f : 0.3 (20 % ethyl acetate in petroleum ether); M.P. = 220-222 °C ; ^{1}H NMR (300 MHz, $CDCl_3$): δ = 11.07 (s, 1H), 10.14 (s, 1H), 7.93 (d, *J* = 7.8 Hz, 1H), 7.78 (d, J = 7.8 Hz, 1H), 7.26 (d, *J* = 8.7 Hz, 2H), 7.20 -7.14 (m, 5H), 7.00 - 6.96 (m, 4H), 6.92 (d, *J* = 8.4 Hz, 2H), 6.64 (d, *J* = 7.5 Hz, 1H), 6.46 (d, *J* = 7.8 Hz, 1H), 5.18 (s, 1H), 4.07 (d, *J* = 13.8 Hz, 1H), 2.41 (d, *J* = 13.8 Hz, 1H); ^{13}C NMR (75 MHz, $CDCl_3$): δ = 195.8, 184.7, 177.6, 142.8, 141.2, 137.7, 137.5, 135.4, 132.3, 131.0, 128.7, 128.2, 127.9, 127.1, 127.0, 126.2, 126.0, 122.7, 120.8, 109.5, 108.7, 83.3, 66.6, 63.9, 54.0, 45.7; HRMS (ES^+): calcd for $[C_{32}H_{22}Cl_2N_2O_4]H^+$: 569.1035; found: 569.1039.

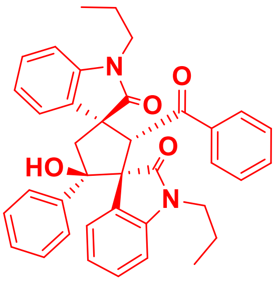

3b: White solid, Yield: 503.3 mg, 86% ; R_f : 0.3 (15 % ethyl acetate in petroleum ether); M.P. = 224-226 °C ; ^{1}H NMR (300 MHz, $CDCl_3$): δ = 8.25 – 8.22 (m, 1H), 7.91(d, *J* = 7.2 Hz, 1H), 7.30 – 7.25 (m, 2H), 7.15-7.03 (m, 12H), 6.97 (s, 1H), 6.66 (d, *J* = 7.5 Hz, 1H), 6.38 - 6.35 (m, 1H), 5.28 (s, 1H), 4.43 (d, *J* = 13.8 Hz, 1H), 3.82 - 3.71 (m, 2H), 3.31 – 3.22 (m, 2H), 2.47 (d, *J* = 13.8 Hz, 1H), 1.71 – 1.62 (m, 2H), 1.06 – 0.96 (m, 5H), 0.65 (t, *J* = 7.2 Hz, 3H) ; ^{13}C NMR (75 MHz, $CDCl_3$): δ = 196.4, 183.6, 176.3, 144.2, 141.9, 138.0, 137.3, 132.0, 130.6, 128.5, 128.2, 127.8, 127.5, 127.3, 127.1, 127.0, 126.8, 125.8, 125.7, 123.9, 121.5, 107.8, 107.6, 84.5, 66.5, 64.4, 54.2, 46.3, 42.3, 41.5, 20.7, 20.2, 11.5, 11.3; HRMS (ES^+): calcd for $[C_{38}H_{36}N_2O_4]H^+$: 585.2753; found: 585.2758.

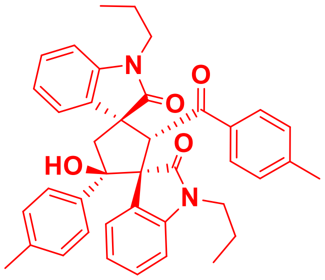

3c: White solid, Yield: 515.5 mg, 84% ; R_f : 0.3 (15 % ethyl acetate in petroleum ether); M.P. = 230-232 °C ; ^{1}H NMR (300 MHz, $CDCl_3$): δ = 8.23 – 8.22 (m, 1H), 7.89 (d, *J* = 7.5 Hz, 1H), 7.28 – 7.23 (m, 2H), 7.10 – 7.04 (m, 5H), 6.96 – 6.90 (m, 6H), 6.66 (d, *J* = 7.5 Hz, 1H), 6.40 (s, 1H), 5.25 (s, 1H), 4.40 (d, *J* = 13.8 Hz, 1H), 3.81 – 3.76 (m, 2H), 3.39 – 3.22 (m, 2H), 2.44 (d, *J* = 13.8 Hz, 1H), 2.24 – 2.22 (m, 6H), 1.68 – 1.63 (m, 2H), 1.23 – 0.95 (m, 5H), 0.62 (t, *J* = 7.2 Hz, 3H); ^{13}C NMR (75 MHz, $CDCl_3$): δ = 195.9, 183.7, 176.5, 144.2, 142.8, 141.9, 137.1, 135.2, 134.7, 130.7, 128.4, 128.1, 127.9, 127.2, 126.9, 125.7, 125.6, 123.9, 121.4, 107.7, 107.6, 84.4, 66.6, 64.2, 54.3, 46.5, 42.3, 41.5, 21.4, 20.8, 20.7, 20.3, 11.5, 11.2; HRMS (ES^+): calcd for $[C_{40}H_{40}N_2O_4]H^+$: 613.7644; found: 613.7649.

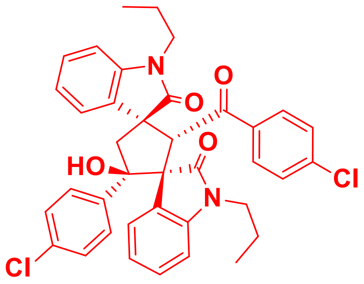

3d: White solid, Yield: 548.7 mg, 84% ; R_f : 0.3 (15 % ethyl acetate in petroleum ether); M.P. = 235-237 °C ; ^{1}H NMR (300 MHz, $CDCl_3$): δ = 8.20 – 8.17 (m, 1H), 7.87 (d, *J* = 7.5 Hz, 1H), 7.28 (t, *J* = 8.7 Hz, 1H), 7.26 – 6.97 (m, 12H), 6.68 (d, *J* = 7.8 Hz, 1H), 6.46 – 6.43 (m, 1H), 5.17 (s, 1H), 4.36 (d, *J* = 13.8 Hz, 1H), 3.83 – 3.71 (m, 2H), 3.38 – 3.26 (m, 2H), 2.45 (d, *J* = 13.8 Hz, 1H), 1.72 – 1.64 (m, 2H), 1.31 – 1.17 (m, 2H), 0.99 (t, *J* = 7.2 Hz, 3H), 0.68 (t, *J* = 7.2 Hz, 3H); ^{13}C NMR (75 MHz, $CDCl_3$): δ = 195.1, 183.4, 176.1, 144.1, 141.9, 138.5, 136.5, 135.5, 133.7, 130.2, 128.8, 128.6, 128.4, 128.1, 127.4, 127.1, 126.2, 125.7, 124.1, 121.7, 108.0, 107.9, 84.1, 66.5, 64.3, 54.1, 46.2, 42.4, 41.6, 20.7, 20.4, 11.5, 11.2; HRMS (ES^+): calcd for $[C_{38}H_{34}Cl_2N_2O_4]H^+$: 653.1974; found: 653.1983.

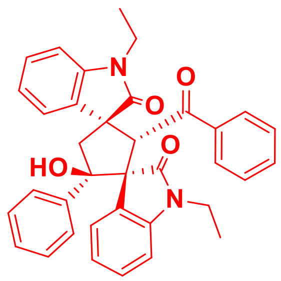

3e: White solid, Yield: 462.5 mg, 83% ; R_f : 0.3 (15 % ethyl acetate in petroleum ether); M.P. = 218-220 °C ; ^{1}H NMR (300 MHz, $CDCl_3$): δ = 8.24 (d, *J* = 3.3 Hz, 1H), 7.91 (d, *J* = 7.2 Hz, 1H), 7.51 – 7.49 (m, 2H), 7.17 – 7.06 (m, 12H), 6.93 (s, 1H), 6.66 (d, *J* = 7.5 Hz, 1H), 6.40 (d, *J* = 4.2 Hz, 1H), 5.29 (s, 1H), 4.42 (d, *J* = 13.8 Hz, 1H), 3.83 (q, *J* = 6.3 Hz, 2H), 3.51 (quintet, *J* = 7.2 Hz, 1H), 3.32 (q, *J* = 7.2 Hz, 1H), 3.47 (d, *J* = 13.8 Hz, 1H), 1.24 (t, *J* = 6.9 Hz, 3H), 0.64 (t, *J* = 6.9 Hz, 3H); ^{13}C NMR (75 MHz, $CDCl_3$): δ = 196.4, 183.2, 176.0, 143.5, 141.6, 138.0, 137.3, 132.1, 130.7, 128.6, 128.3, 127.9, 127.5, 127.3, 127.2, 127.0, 126.8, 125.8, 125.7, 124.0, 121.5, 107.7, 107.5, 84.5, 66.7, 64.0, 54.2, 46.1, 35.5, 34.1, 12.5, 11.5; HRMS (ES^+): calcd for $[C_{36}H_{32}N_2O_4]H^+$: 557.2440; found: 557.2447.

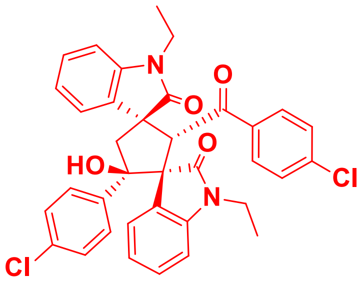

3f: White solid, Yield: 411.8 mg, 82% ; R_f : 0.3 (15 % ethyl acetate in petroleum ether); M.P. = 231-232 °C ; ^{1}H NMR (300 MHz, $CDCl_3$): δ = 8.27 – 8.24 (m, 1H), 7.92 – 7.90 (m, 1H), 7.29 – 7.26 (m, 2H), 7.17 – 7.03 (m, 11H), 6.94 (s, 1H), 6.66 (d, *J* = 6 Hz, 1H), 6.41 – 6.38 (m, 1H), 5.29 (s, 1H), 4.43 (d, *J* = 13.8 Hz, 1H), 3.87 – 3.79 (m, 2H), 3.54 – 3.51 (m, 1H), 3.33 – 3.31 (m, 1H), 2.48 (d, *J* = 13.8 Hz, 1H), 1,24 (t, *J* = 7.2 Hz, 3H), 0.64 (t, *J* = 7.2 Hz, 3H); ^{13}C NMR (75 MHz, $CDCl_3$): δ = 196.4, 183.1, 176.0, 143.4, 141.5, 137.9, 137.2, 132.0, 130.6, 128.5, 128.2, 127.8, 127.5, 127.2, 127.1, 127.0, 125.7, 125.6, 123.9, 121.5, 107.6, 107.4, 84.5, 66.6, 63.9, 54.1, 46.0, 35.5, 34.1, 12.5, 11.4; HRMS (ES^+): calcd for $[C_{36}H_{30}Cl_2N_2O_4]H^+$: 625.1661; found: 625.1665.

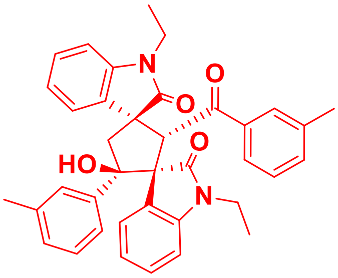

3g: White solid, Yield: 467.4 mg, 80% ; R_f : 0.3 (15 % ethyl acetate in petroleum ether); M.P. = 223-225 °C ; ^{1}H NMR (300 MHz, $CDCl_3$): δ = 8.16 – 8.13 (m, 1H), 7.80 – 7.77 (m, 1H), 7.21 – 7.15 (m, 1H), 7.03 – 6.93 (m, 5H), 6.84 – 6.77 (m, 6H), 6.56 (d, *J* = 7.8 Hz, 1H), 6.34 – 6.31 (m, 1H), 5.16 (s, 1H), 4.29 (d, *J* = 13.8 Hz, 1H), 3.78 – 3.69 (m, 2H), 3.46 (sextet, *J* = 6.9 Hz, 1H), 3.23 (sextet, *J* = 6.9 Hz, 1H), 2.35 (d, *J* = 13.8 Hz, 1H), 2.14 – 2.12 (m, 6H), 1.26 (t, *J* = 7.2 Hz, 3H), 0.54 (t, *J* = 7.2 Hz, 3H); ^{13}C NMR (75 MHz, $CDCl_3$): δ = 196.0, 183.3, 176.2, 143.5, 142.9, 141.6, 137.1, 135.0, 134.7, 130.8, 129.4, 128.6, 128.5, 128.2, 127.9, 127.2, 126.9, 125.7, 125.6, 124.0, 121.5, 107.6, 107.4, 84.5, 66.7, 63.8, 54.3, 46.2, 35.5, 34.1, 21.5, 20.9, 12.5, 11.4; HRMS (ES^+): calcd for $[C_{38}H_{36}N_2O_4]H^+$: 585.2753; found: 585.2760.

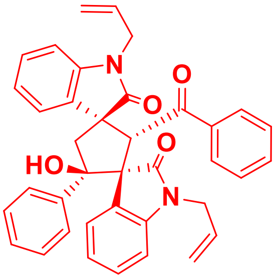

3h: White solid, Yield: 458.4 mg, 79% ; R_f : 0.3 (15 % ethyl acetate in petroleum ether); M.P. = 215-217 °C ; ^{1}H NMR (400 MHz, $CDCl_3$): δ = 8.24 – 8.22 (m, 1H), 7.90 (d, J = 7.2 Hz, 1H), 7.29 – 7.27 (m, 1H), 7.24 – 7.22 (m, 1H), 7.15 – 7.13 (m, 2H), 7.12 – 7.11 (m, 2H), 7.10 – 7.09 (m, 2H), 7.08 – 7.07 (m, 4H), 7.05 (s, 1H), 7.03 – 7.02 (m, 1H), 6.88 (s, 1H), 6.60 (d, *J* = 7.6 Hz, 1H), 6.36 (d, *J* = 7.6 Hz, 1H), 5.73 – 5.71 (m, 1H), 5.29 (s, 1H), 5.24 – 5.20 (m, 2H), 5.19 – 5.16 (m, 1H), 4.86 – 4.83 (m, 1H), 4.56 – 4.43 (m, 3H), 4.42 – 4,38 (m, 2H), 3.95 – 3.87 (m, 3H), 2.48 (d, *J* = 14 Hz, 1H); ^{13}C NMR (100 MHz, $CDCl_3$): δ = 196.4, 183.3, 176.3, 143.6, 141.6, 137.9, 137.2, 132.2, 131.1, 130.5, 130.2, 128.6, 128.3, 127.9, 127.6, 127.0, 126.4, 125.8, 124.2, 121.7, 118.5, 116.5, 108.4, 108.3, 84.5, 66.7, 64.4, 54.2, 46.2, 43.1, 42.0, 29.7; HRMS (ES^+): calcd for $[C_{38}H_{32}N_2O_4]H^+$: 581.2440; found: 581.2452.

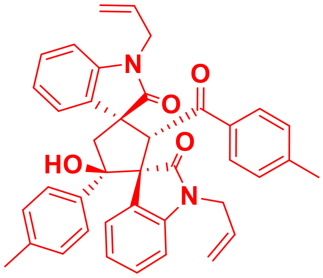

3i: White solid, Yield: 474.4 mg, 78% ; R_f : 0.3 (15 % ethyl acetate in petroleum ether); M.P. = 229-231 °C ; ^{1}H NMR (400 MHz, $CDCl_3$): δ = 8.23 – 8.21 (m, 1H), 7.88 (d, J = 7.6 Hz, 1H), 7.22 (t, *J* = 7.6 Hz, 1H), 7.09 – 6.99 (m, 5H), 6.94 (d, *J* = 8,4 Hz, 2H), 6.90 – 6.87 (m, 4H), 6.78 (s, 2H), 6.59 (d, *J* = 7.6 Hz, 1H), 6.39 (d, *J* = 6.8 Hz, 1H), 5.72 (sextet, *J* = 6 Hz, 1H), 5.29 – 5.18 (m, 4H), 4.83 (d, *J* = 10.4 Hz, 1H), 4.51 – 4.37 (m, 4H), 4.00 (q, *J* = 10 Hz, 1H), 3.88 (q, *J* = 11.2 Hz, 1H), 2.45 (d, *J* = 14 Hz, 1H), 2,24 – 2.22 (m, 6H); ^{13}C NMR (100 MHz, $CDCl_3$): δ = 196.1, 183.6, 176.6, 143.9, 143.0, 141.8, 137.3, 135.3, 134.9, 131.5, 130.9, 130.7, 128.7, 128.6, 128.3, 128.2, 127.4, 126.9, 126.0, 125.8, 124.3, 121.8, 118.5, 116.3, 108.5, 108.4, 84.7, 67.0, 64.5, 54.5, 46.7, 43.3, 42.2, 21.6, 21.0 ; HRMS (ES^+): calcd for $[C_{40}H_{36}N_2O_4]H^+$: 609. 2753; found: 609.2760.

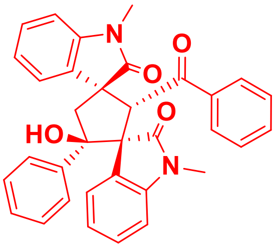

3j: White solid, Yield: 417.2 mg, 79% ; R_f : 0.3 (15 % ethyl acetate in petroleum ether); M.P. = 222-224 °C ; ^{1}H NMR (300 MHz, $CDCl_3$): δ = 8.32 – 8.29 (m, 1H), 7.89 (d, *J* = 6.9 Hz, 1H), 7.32 – 7.27 (m, 2H), 7.16 – 7.04 (m, 12H), 6.92 (s, 1H), 6.64 (d, *J* = 7.8 Hz, 1H), 6.31 – 6.29 (m, 1H), 5.26 (s, 1H), 4.39 (d, *J* = 13.8 Hz, 1H), 3.11 (s, 3H), 2.92 (s, 3H), 2.48 (d, *J* = 14.1 Hz, 1H); ^{13}C NMR (75 MHz, $CDCl_3$): δ = 196.4, 183.5, 176.5, 144.4, 142.4, 137.7, 137.1, 132.1, 130.3, 128.6, 128.4, 127.6, 127.5, 127.1, 126.9, 126.8, 126.5, 125.6, 125.5, 124.2, 121.7, 107.5, 107.4, 84.5, 66.8, 64.4, 54.3, 45.5, 26.6, 25.8 ; HRMS (ES^+): calcd for $[C_{34}H_{28}N_2O_4]H^+$: 529.2127; found: 529.2131.

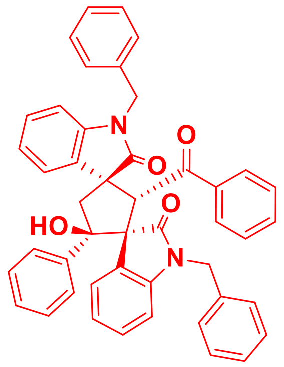

3k [*2*]: White solid, Yield: 496.6 mg, 73% ; R_f : 0.3 (15 % ethyl acetate in petroleum ether); M.P. = 223-225 °C ; ^{1}H NMR (300 MHz, $CDCl_3$): δ = 8.22 – 8.20 (m, 1H), 7.98 – 7.96 (m, 1H), 7.37 – 7.33 (m, 2H), 7.31 – 7.30 (m, 2H), 7.29 – 7.26 (m, 3H), 7.16 – 7.15 (m, 5H), 7.13 – 7.10 (m, 4H), 7.09 – 7.08 (m, 2H), 7.03 – 7.02 (m, 2H), 7.01 – 7.00 (m, 2H), 6.98 – 6.92 (m, 1H), 6.51 (d, J = 6.8 Hz, 2H), 6.40 (d, J = 8 Hz, 1H), 6.30 (d, J = 7.6 Hz, 1H), 5.30 (s, 1H), 5.26 – 5.17 (m, 2H), 4.53 – 4.44 (m, 2H), 4.26 (d, J = 15.6 Hz, 1H), 2.57 (d, J = 14 Hz, 1H); ^{13}C NMR (75 MHz, $CDCl_3$): δ = 196.4, 183.8, 176.8, 143.7, 141.5, 138.2, 137.0, 135.6, 134.8, 132.3, 130.3, 128.9, 128.5, 128.3, 127.8, 127.6, 127.5, 127.2, 127.0, 126.9, 126.8, 126.6, 126.3, 126.0, 125.7, 124.2, 121.9, 108.7, 108.5, 84.5, 66.5, 65.2, 54.2, 46.6, 44.5, 43.7 ; HRMS (ES^+): calcd for $[C_{34}H_{28}N_2O_4]H^+$: 681.2753; found: 681.2759.

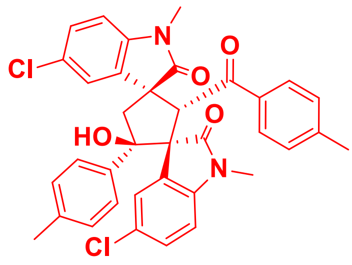

3l: White solid, Yield: 468 mg, 75% ; R_f : 0.3 (15 % ethyl acetate in petroleum ether); M.P. = 225-227 °C ; ^{1}H NMR (300 MHz, $CDCl_3$): δ = 8.30 – 8.27 (m, 1H), 7.87 (d, J = 7.2 Hz, 1H), 7.31 – 7.25 (m, 1H), 7.13 – 7.06 (m, 5H), 7.05 – 7.02 (m, 2H), 6.98 – 6.86 (m, 5H), 6.64 (d, J = 7.8 Hz, 1H), 6.34 – 6.31 (m, 1H), 5.22 (s, 2H), 4.36 (d, J = 13.8 Hz, 1H), 3.13 (s, 1H), 2.95 (s, 1H), 2.45 (d, J = 14.1 Hz, 1H), 2.26 – 2.24 (m, 1H); ^{13}C NMR (75 MHz, $CDCl_3$): δ = 196.0, 183.6, 176.7, 144.5, 143.0, 142.4, 137.0, 135.0, 134.6, 130.4, 129.4, 128.6, 128.3, 128.2, 127.9, 127.1, 127.0, 126.8, 125.5, 124.2, 121.7, 107.4, 107.3, 84.5, 66.8, 64.5, 54.4, 45.8, 26.7, 25.9, 21.5, 21.0; HRMS (ES^+): calcd for $[C_{36}H_{30}Cl_2N_2O_4]H^+$: 625.1661; found: 625.1665.

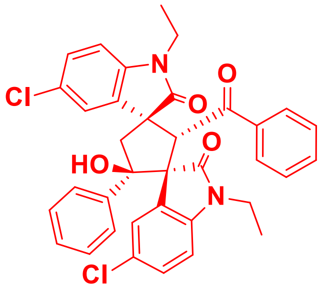

3m: White solid, Yield: 474.4 mg, 76% ; R_f : 0.3 (15 % ethyl acetate in petroleum ether); M.P. = 233 - 234 °C ; ^{1}H NMR (300 MHz, $CDCl_3$): δ = 8.26 (d, *J* = 2.4 Hz, 1H), 7.88 (d, *J* = 2.1 Hz, 1H), 7.31 – 7.21 (m, 2H), 7.19 – 7.02 (m, 10H), 6.84 (s, 1H), 6.60 – 6.51 (m, 1H), 6.32 (d, *J* = 8.1 Hz, 1H), 5.19 (s, 1H), 4.36 (d, *J* = 14.1 Hz, 1H), 3.86 – 3.79 (m, 2H), 3.52 – 3.27 (m, 2H), 2.46 (d, *J* = 13.8 Hz, 1H), 1.24 (t, *J* = 7.2 Hz, 3H), 0.62 (t, *J* = 7.2 Hz, 3H); ^{13}C NMR (75 MHz, $CDCl_3$): δ = 196.1, 182.7, 175.6, 142.2, 142.0, 140.7, 140.2, 137.4, 137.1, 137.0, 136.6, 132.4, 132.2, 129.5, 129.2, 128.6, 128.5, 128.4, 128.1, 128.0, 127.8, 127.6, 127.5, 127.4, 127.3, 127.2, 127.1, 126.9, 126.8, 126.7, 126.3, 126.2, 126.1, 125.7, 108.7, 108.4, 84.5, 66.9, 64.1, 54.2, 45.8, 35.5, 34.3, 12.5, 11.5; HRMS (ES$^+$): calcd for $[C_{36}H_{30}Cl_2N_2O_4]H^+$: 625.1661; found: 625.1670.

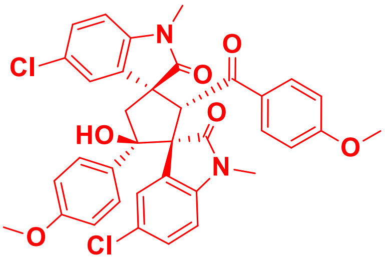

3n: White solid, Yield: 506 mg, 77% ; R_f : 0.3 (15 % ethyl acetate in petroleum ether); M.P. = 219-220 °C ; ^{1}H NMR (300 MHz, $CDCl_3$): δ = 8.30 – 8.27 (m, 1H), 7.67 (d, *J* = 8.1 Hz, 1H), 7.31 – 7.24 (m, 1H), 7.13 – 7.07 (m, 5H), 7.05 – 7.02 (m, 2H), 6.98 – 6.86 (m, 5H), 6.64 (d, *J* = 7.8 Hz, 1H), 6.33 – 6.31 (m, 1H), 5.22 (s, 1H), 4.36 (d, *J* = 13.8 Hz, 1H), 3.84-3.72 (m, 6H), 3.13 (s, 3H), 2.95 (s, 3H), 2.44 (d, *J* = 14.1 Hz, 1H); ^{13}C NMR (75 MHz, $CDCl_3$): δ = 195.9, 183.6, 176.7, 144.4, 143.0, 142.4, 137.0, 134.9, 134.5, 130.4, 129.4, 128.6, 128.4, 128.3, 127.9, 127.1, 126.9, 126.7, 125.5, 124.1, 121.6, 107.4, 84.5, 66.8, 64.5, 56.5, 55.5, 54.4, 45.8, 26.7, 25.9; HRMS (ES$^+$): calcd for $[C_{36}H_{30}Cl_2N_2O_6]H^+$: 657.1559; found: 657.1565.

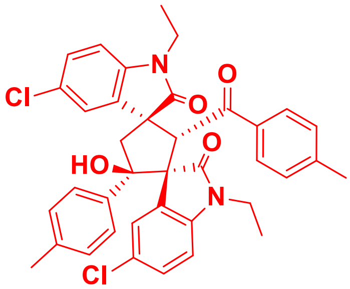

3o: White solid, Yield: 502 mg, 77% ; R_f : 0.3 (15 % ethyl acetate in petroleum ether); M.P. = 228 - 230 °C ; ^{1}H NMR (300 MHz, $CDCl_3$): δ = 8.25 (d, *J* = 2.1 Hz, 1H), 7.85 (d, *J* = 2.1 Hz, 1H), 7.27 – 7.24 (m, 1H), 7.11 (d, *J* = 8.4 Hz, 2H), 7.06 – 7.02 (m, 1H), 6.97 – 6.92 (m, 6H), 6.77 (s, 1H), 6.57 (d, *J* = 8.4 Hz, 1H), 6.34 (d, *J* = 8.4 Hz, 1H), 5.15 (s, 1H), 4.32 (d, *J* = 14.1 Hz, 1H), 3.87 – 3.79 (m, 2H), 3.55 – 3.52 (m, 2H), 3.32 – 3.27 (m, 1H), 2.43 (d, *J* = 13.8 Hz, 1H), 2.26 – 2.23 (m, 6H), 1.24 (t, *J* = 7.2 Hz, 3H), 0.63 (t, *J* = 7.2 Hz, 3H); ^{13}C NMR (75 MHz, $CDCl_3$): δ = 195.6, 182.8, 175.5, 143.2, 142.2, 142.1, 140.7, 140.2, 137.4, 137.2, 134.5, 134.4, 133.7, 132.3, 129.5, 129.1, 128.8, 128.7, 128.5, 128.4, 128.0, 127.9, 127.4, 127.2, 126.8, 126.7, 126.3, 126.2, 126.1, 125.6, 108.6, 108.3, 84.4, 66.9, 64.0, 54.3, 46.0, 35.7, 34.3, 21.5, 20.9, 12.5, 11.4; HRMS (ES^+): calcd for $[C_{38}H_{34}Cl_2N_2O_4]H^+$: 653.1974; found: 653.1982.

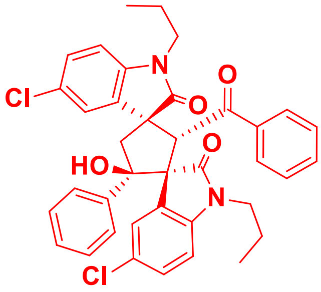

3p: White solid, Yield: 508.6 mg, 78% ; R_f : 0.3 (15 % ethyl acetate in petroleum ether); M.P. = 213 - 215 °C ; ^{1}H NMR (300 MHz, $CDCl_3$): δ = 8.25 (d, *J* = 1.5 Hz, 1H), 7.88 (d, *J* = 1.8 Hz, 1H), 7.31 – 7.24 (m, 2H), 7.21 – 7.11 (m, 9H), 7.04 – 7.00 (m, 1H), 6.88 (s, 1H), 6.60 – 6.56 (m, 1H), 6.29 (d, *J* = 8.1 Hz, 1H), 5.18 (s, 1H), 4.37 (d, *J* = 15 Hz, 1H), 3.78 – 3.74 (m, 2H), 3.28 – 3.16 (m, 2H), 2.46 (d, *J* = 13.8 Hz, 1H), 1.70 – 1.61 (m, 4H), 0.98 (t, *J* = 7.5 Hz, 3H), 0.66 (t, *J* = 7.2 Hz, 3H); ^{13}C NMR (75 MHz, $CDCl_3$): δ = 196.1, 183.1, 175.9, 142.9, 140.5, 137.5, 137.1, 132.4, 132.1, 129.5, 128.7, 128.6, 128.4, 128.3, 128.0, 127.9, 127.7, 127.5, 127.3, 127.2, 127.1, 126.8, 126.7, 126.3, 126.0, 125.7, 108.9, 108.6, 84.5, 66.7, 64.6, 54.2, 46.1, 42.5, 41.7, 20.6, 20.2, 11.5, 11.3; HRMS (ES^+): calcd for $[C_{38}H_{34}Cl_2N_2O_4]H^+$: 653.1974; found: 653.1985.

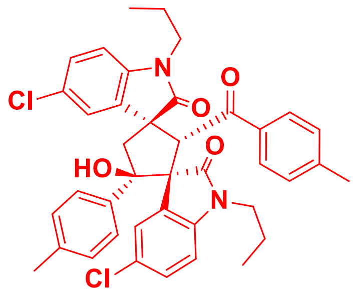

3q: White solid, Yield: 503.2 mg, 74% ; R_f : 0.3 (15 % ethyl acetate in petroleum ether); M.P. = 217 - 219 °C ; ^{1}H NMR (300 MHz, $CDCl_3$): δ = 8.23 (d, J = 2.1 Hz, 1H), 7.86 (d, J = 2.1 Hz, 1H), 7.26 – 7.22 (m, 1H), 7.11 – 7.08 (m, 2H), 7.04 – 7.01 (m, 1H), 6.99 – 6.91 (m, 6H), 6.81 (s, 1H), 6.58 (d, J = 8.4 Hz, 1H), 6.32 (d, J = 8.4 Hz, 1H), 5.15 (s. 1H), 4.34 (d, J = 13.8 Hz, 1H), 3.82 – 3.74 (m, 2H), 3.31 – 3.18 (m, 2H), 2.42 (d, J = 14.1 Hz, 1H), 2.27 – 2.23 (m, 6H), 1.72 – 1.62 (m, 4H), 0.98 (t, J = 7.2 Hz, 3H), 0.62 (t, J = 7.2 Hz, 3H); ^{13}C NMR (75 MHz, $CDCl_3$): δ = 195.6, 183.2, 176.1, 143.2, 142.9, 140.5, 137.5, 134.6, 134.5, 132.2, 129.4, 128.7, 128.5, 128.4, 128.3, 128.1, 127.4, 127.2, 126.7, 126.0, 125.6, 108.8, 108.6, 84.4, 66.8, 64.4, 54.3, 46.2, 42.5, 41.6, 21.5, 20.9, 20.7, 20.3, 11.5, 11.2; HRMS (ES$^+$): calcd for $[C_{40}H_{32}Cl_2N_2O_4]H^+$: 681.2287; found: 681.2294.

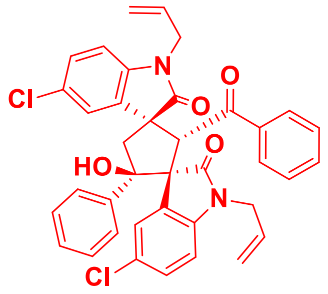

3r: White solid, Yield: 466.6 mg, 72% ; R_f : 0.3 (15 % ethyl acetate in petroleum ether); M.P. = 227 - 229 °C ; ^{1}H NMR (300 MHz, $CDCl_3$): δ = 8.28 (s, 1H), 7.92 (dd, J_1 = 2.1 Hz, J_2 = 13.5 Hz, 1H), 7.33 – 7.32 (m, 1H), 7.25 – 7.13 (m, 9H), 7.03 – 7.00 (m, 1H), 6.78 – 6.39 (m, 1H), 6.52 (dd, J_1 = 8.4 Hz, J_2 = 13.5 Hz, 1H), 6.30 (dd, J_1 = 1.8 Hz, J_2 = 8.4 Hz, 1H), 5.80 – 5.67 (m, 1H), 5.27 – 5.20 (m. 4H), 4.88 (t, J = 9.6 Hz, 1H), 4.61 – 4.35 (m, 4H), 3.95 – 3.79 (m, 2H), 3.40 – 3.09 (m, 1H), 2.50 – 2.19 (m, 1H); ^{13}C NMR (75 MHz, $CDCl_3$): δ = 196.1, 179.0, 176.3, 142.4, 142.2, 140.9, 140.2, 137.4, 137.1, 137.0, 136.8, 132.5, 130.8, 130.7, 130.3, 130.1, 129.7, 129.3, 128.6, 128.5, 128.1, 127.9, 127.6, 127.5, 127.2, 127.1, 126.7, 126.2, 125.8, 118.9, 116.9, 109.4, 109.2, 87.7, 84.4, 68.1, 67.0, 65.0, 64.7, 62.0, 59.0, 54.2, 43.3, 43.2, 42.3 ; HRMS (ES$^+$): calcd for $[C_{38}H_{30}Cl_2N_2O_4]H^+$: 649.1661; found: 649.1667.

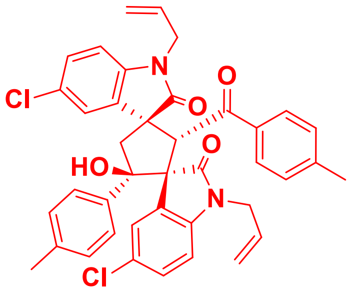

3s: White solid, Yield: 459.8 mg, 68% ; R_f : 0.3 (15 % ethyl acetate in petroleum ether); M.P. = 220 - 222 °C ; ^1H NMR (300 MHz, $CDCl_3$): δ = 8.26 – 8.25 (m, 1H), 7.92 – 7.87 (m, 1H), 7.57 – 7.44 (m, 1H), 7.24 – 7.09 (m, 4H), 7.04 – 7.01 (m, 2H), 7.00 – 6.92 (m, 3H), 6.55 – 6.48 (m, 1H), 6.33 (d, *J* = 9 Hz, 1H), 6.06 – 5.92 (m, 1H), 5.80 – 5.69 (m. 1H), 5.32 – 5.19 (m, 4H), 4.85 (t, *J* = 8.4 Hz, 1H), 4.64 – 4.46 (m, 2H), 4.39 – 4.32 (m, 1H), 4.01 – 3.78 (m, 2H), 3.40 – 3.10 (m, 1H), 2.47 – 2.41 (m, 1H), 2.25 – 2.19 (m, 6H); ^{13}C NMR (75 MHz, $CDCl_3$): δ = 195.6, 182.9, 176.0, 143.3, 142.3, 140.2, 137.5, 134.5, 134.4, 132.7, 132.1, 130.9, 130.7, 130.3, 130.2, 129.6, 129.2 128.7, 128.6, 128.5, 128.3, 128.2, 128.1, 127.3, 127.2, 127.0, 126.7, 126.6, 126.2, 125.7, 118.7, 118.6, 116.4, 109.3, 109.1, 87.8, 84.4, 67.0, 64.5, 61.8, 59.0, 54.3, 46.2, 43.3 42.2, 30.9, 21.5, 20.8 ; HRMS (ES^+): calcd for $[C_{40}H_{34}Cl_2N_2O_4]H^+$: 677.1974; found: 677.1979.

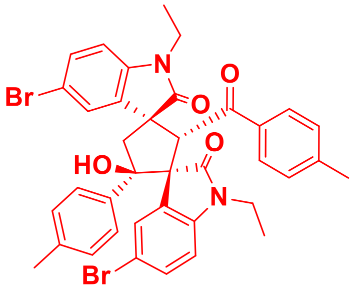

3t: White solid, Yield: 554.8 mg, 75% ; R_f : 0.3 (15 % ethyl acetate in petroleum ether); M.P. = 237 - 239 °C ; ^1H NMR (300 MHz, $CDCl_3$): δ = 8.37 – 8.36 (m, 1H), 8.02 – 7.98 (m, 1H), 7.42 – 7.38 (m, 1H), 7.21 – 7.18 (m, 1H), 7.15 – 7.05 (m, 3H), 7.02 – 6.91 (m, 5H), 6.53 (d, *J* = 8.1 Hz, 1H), 6.40 – 6.28 (m, 1H), 5.19 – 5.14 (m, 1H), 4.32 (d. *J* = 13.8 Hz, 1H), 3.89 – 3.78 (m, 2H), 3.56 – 3.51 (m, 1H), 3.33 – 3.27 (m, 1H), 2.42 (d, *J* = 13.8 Hz, 1H), 2.26 – 2.19 (m, 6H), 1.18 – 1.21 (m, 4H), 0.68 – 0.60 (m, 3H); ^{13}C NMR (75 MHz, $CDCl_3$): δ = 195.6, 182.6, 175.5, 143.1, 142.6, 140.6, 137.4, 134.5, 132.6, 131.4, 131.3, 130.1, 128.9, 128.7, 128.6, 128.0, 127.9, 127.3, 127.2, 126.1, 125.6, 116.7, 114.0, 109.0, 108.8, 84.4, 66.8, 64.0, 54.2, 45.9, 35.6, 34.2, 21.4, 20.9, 12.4, 11.2; HRMS (ES^+): calcd for $[C_{38}H_{34}Br_2N_2O_4]H^+$: 743.0943; found: 743.0949.

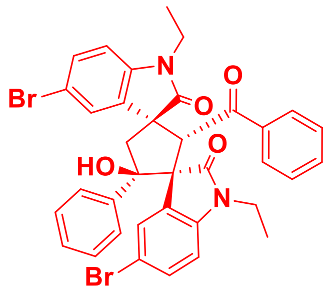

3u: White solid, Yield: 540.9 mg, 76% ; R_f : 0.3 (15 % ethyl acetate in petroleum ether); M.P. = 232 - 234 °C ; ^{1}H NMR (300 MHz, $CDCl_3$): δ = 8.38 (s, 1H), 8.02 – 8.0 (m, 1H), 7.43 – 7.40 (m, 1H), 7.31 – 7.28 (m, 1H), 7.21 – 6.99 (m, 10H), 6.83 (s, 1H), 6.54 (d, *J* = 8.4 Hz, 1H), 6.27 (d, *J* = 8 Hz, 1H), 5.21 – 5.18 (m, 1H), 4.35 (d. *J* = 13.8 Hz, 1H), 3.89 – 3.79 (m, 2H), 3.53 – 3.49 (m, 1H), 3.31 – 3.26 (m, 1H), 2.45 (d, *J* = 13.8 Hz, 1H), 1.24 (t, *J* = 7.2 Hz, 3H), 0.60 (t, *J* = 7.2 Hz, 3H); ^{13}C NMR (75 MHz, $CDCl_3$): δ = 196.1, 182.5, 175.4, 142.6, 140.6, 137.3, 137.1, 132.5, 132.3, 131.5, 131.4, 130.0, 128.7, 128.1, 128.0, 127.9, 127.8, 127.4, 127.3, 127.2, 127.1, 127.0, 125.7, 116.8, 114.1, 109.2, 108.9, 84.4, 66.8, 64.1, 54.1, 45.7, 35.7, 34.3, 14.4, 11.4; HRMS (ES^+): calcd for $[C_{36}H_{30}Br_2N_2O_4]H^+$: 715.0630; found: 715.0638.

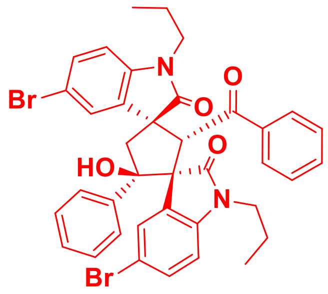

3v: White solid, Yield: 577.5 mg, 78% ; R_f : 0.3 (15 % ethyl acetate in petroleum ether); M.P. = 225 - 227 °C ; ^{1}H NMR (300 MHz, $CDCl_3$): δ = 8.36 (d, *J* = 2.1 Hz, 1H), 8.04 (d, *J* = 1.8 Hz, 1H), 7.40 – 7.37 (m, 1H), 7.32 – 7.27 (m, 1H), 7.21 – 7.10 (m, 9H), 6.62 (s, 1H), 6.51 (d, *J* = 8.4 Hz, 1H), 6.26 (d, *J* = 8.4 Hz, 1H), 6.03 (s, 1H), 3.94 – 3.89 (m, 1H), 3.74 – 3.71 (m, 1H), 3.24 – 3.09 (m, 4H), 1.73 – 1.61 (m, 4H), 1.01 (t, *J* = 7.2 Hz, 3H), 0.68 (t, *J* = 7.2 Hz, 3H); ^{13}C NMR (75 MHz, $CDCl_3$): δ = 195.9, 179.1, 176.1, 143.2, 141.5, 137.0, 136.5, 132.6, 132.4, 131.5, 131.4, 129.4, 128.9, 128.5 128.4, 128.0, 127.7, 127.6, 127.4, 127.1, 126.2, 116.5, 114.3, 109.1, 89.0, 84.5, 64.5, 61.9, 60.3, 58.5, 42.4, 41.7, 20.7, 20.2, 11.3; HRMS (ES^+): calcd for $[C_{38}H_{34}Br_2N_2O_4]H^+$: 743.0943; found: 743.0949.

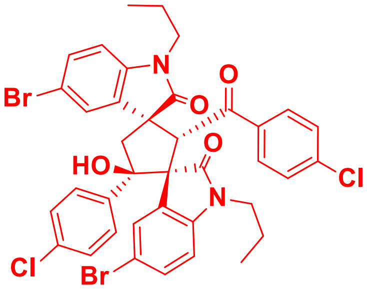

3w: White solid, Yield: 565.8 mg, 70% ; R_f : 0.2 (15 % ethyl acetate in petroleum ether); M.P. = 245 - 247 °C ; ^{1}H NMR (300 MHz, $CDCl_3$): δ = 8.21 (d, *J* = 1.8 Hz, 1H), 7.88 – 7.87 (m, 1H), 7.26 – 7.22 (m, 1H), 7.19 – 7.07 (m, 8H), 6.62 – 6.53 (m, 2H), 6.40 – 6.37 (m, 1H), 6.03 – 5.96 (m, 1H), 5.13 (s, 1H), 3.98 – 3.85 (m, 1H), 3.79 – 3.68 (m, 1H), 3.30 – 3.25 (m, 2H), 3.09 (s, 1H), 1.68 – 1.60 (m, 3H), 1.34 – 1.24 (m, 2H), 1.02 (t, *J* = 7.5 Hz, 3H), 0.72 (t, *J* = 6 Hz, 3H); ^{13}C NMR (75 MHz, $CDCl_3$): δ = 194.6, 179.0, 176.0, 142.6, 141.0, 138.9, 135.2, 133.9, 131.9, 129.4, 128.9, 128.8, 128.6, 128.5, 128.2, 127.8, 127.7, 127.6, 127.5, 127.4, 127.1, 126.7, 126.6, 126.2, 108.8, 89.0, 84.0, 64.4, 61.8, 60.4, 58.4, 42.5, 41.8, 20.4, 15.1, 11.2; HRMS (ES^+): calcd for $[C_{38}H_{32}Br_2Cl_2N_2O_4]H^+$: 811.0163; found: 811.0174.

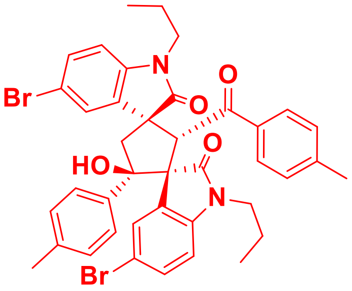

3x: White solid, Yield: 576.0 mg, 75% ; R_f : 0.3 (15 % ethyl acetate in petroleum ether); M.P. = 230 - 232 °C ; ^{1}H NMR (300 MHz, $CDCl_3$): δ = 8.35 – 8.34 (m, 1H), 7.80 – 7.77 (m, 2H), 7.57 – 7.54 (m, 1H), 7.50 – 7.40 (m, 2H), 7.38 – 7.34 (m, 1H), 7.12 – 7.07 (m, 2H), 7.06 – 6.91 (m, 3H), 6.55 – 6.46 (m, 1H), 6.26 (d, *J* = 10.8 Hz, 1H), 4.33 (d, J = 13.8 Hz,1H), 3.82 – 3.74 (m, 2H), 3.35 – 3.16 (m, 2H), 2.42 (d, *J* = 14.1 Hz, 1H), 2.27 – 2.23 (m, 6H), 1.69 – 1.62 (m, 3H), 1.28 – 1.16 (m, 1H), 1.01 – 0.96 (m, 3H), 0.67 – 0.60 (m, 3H); ^{13}C NMR (75 MHz, $CDCl_3$): δ = 195.6, 183.1, 175.9, 143.4, 143.1, 141.0, 137.4, 136.6, 134.6, 134.5, 132.7, 132.5, 131.3, 131.2, 130.0, 128.8, 128.6, 128.1, 127.9, 127.3, 127.1, 126.6, 126.2, 116.7, 114.0, 109.2, 109.0, 84.4, 66.7, 64.4, 54.2, 46.1, 42.4, 41.6, 21.5, 20.9, 20.6, 20.2, 11.5, 11.1; HRMS (ES^+): calcd for $[C_{40}H_{38}Br_2N_2O_4]H^+$: 771.1256; found: 771.1262.

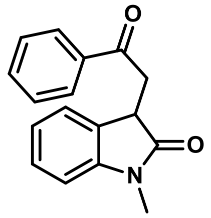

1-methyl-3-(2-oxo-2-phenylethyl)indolin-2-one [*3*]

4j: White solid, Yield: 106.0 mg, 80% ; R_f : 0.3 (20 % ethyl acetate in petroleum ether); M.P = 170-172 °C; ^{1}H NMR (300 MHz, $CDCl_3$): δ = 8.00 (d, *J* = 7.5 Hz, 2H), 7.60 (t, *J* = 7.2 Hz, 1H), 7.49 (t, *J* = 7.5 Hz, 2H), 7.46 – 7.26 (m, 2H), 7.01 (t, *J* = 7.5 Hz, 1H), 6.88 (d, *J* = 7.5 Hz, 1H), 4.11 (d, *J* = 8.1 Hz, 1H), 3.86 (d, *J* = 18 Hz, 1H), 3.46 – 3.38 (m, 1H), 3.30 (s, 3H); ^{13}C NMR (75 MHz, $CDCl_3$): δ = 183.4, 172.0, 150.4, 141.0, 132.1, 128.9, 128.5, 128.4, 126.9, 124.6, 113.0, 109.0, 55.1, 34.9, 24.2.

^{1}H NMR and ^{13}C NMR Spectra of compounds

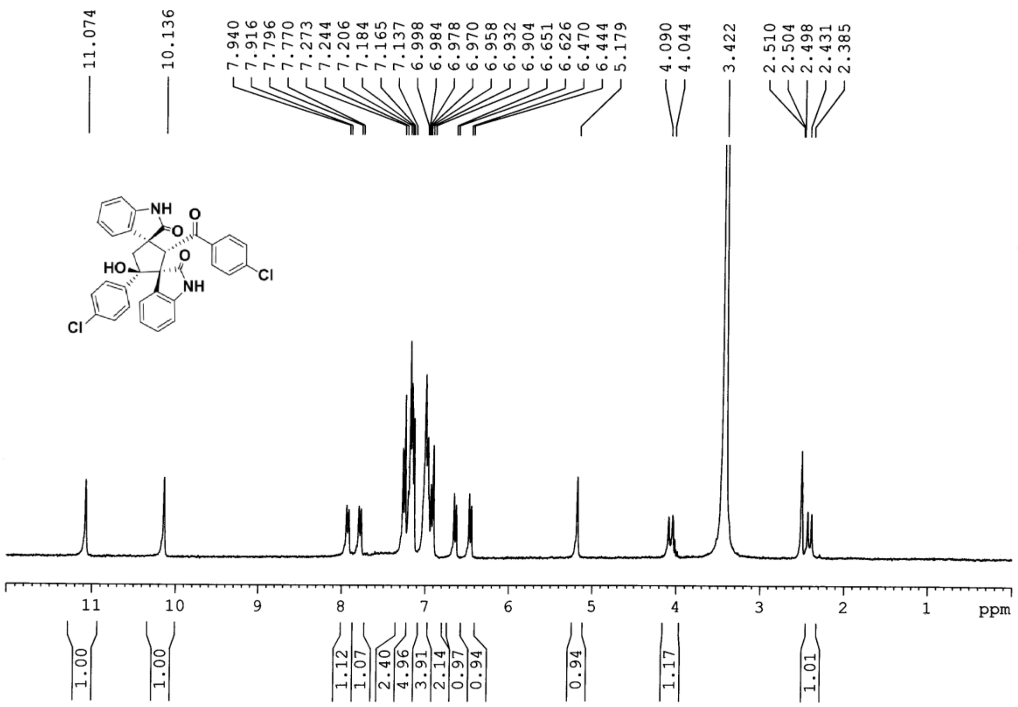

^{1}H NMR Spectrum of compound **3a**

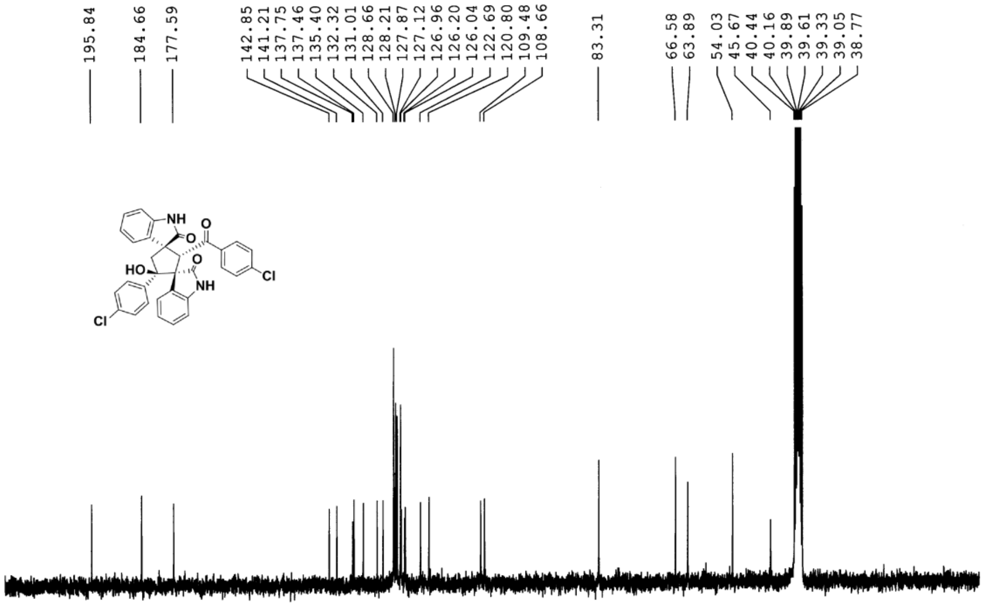

^{13}C NMR Spectrum of compound **3a**

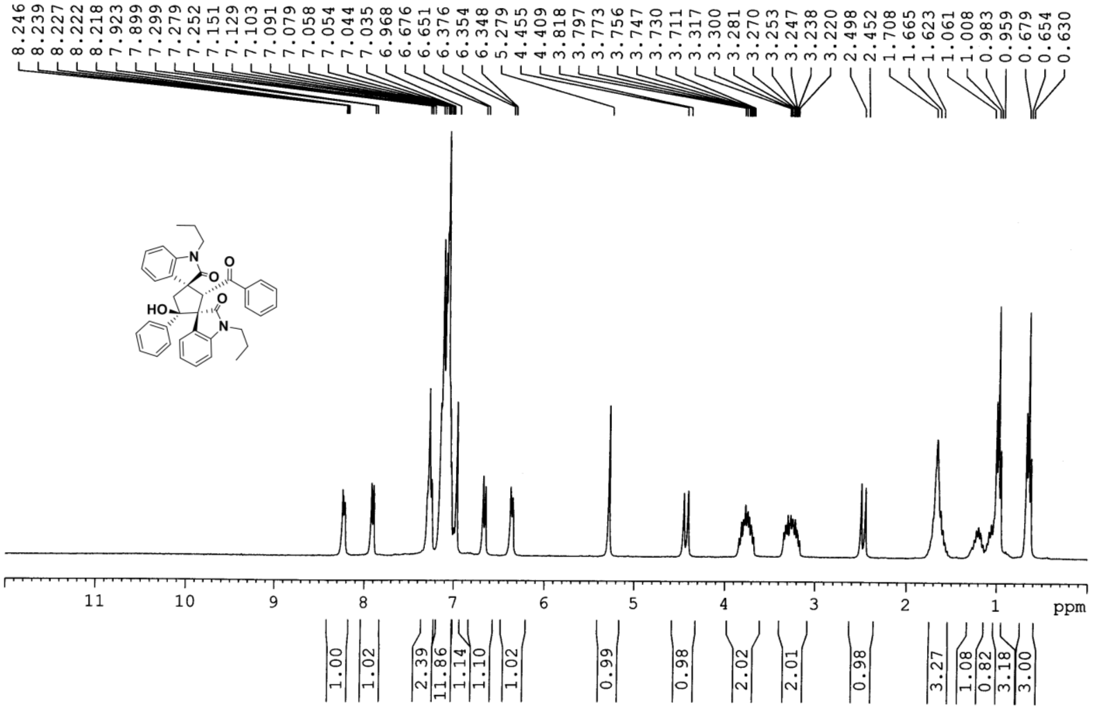

^{1}H NMR Spectrum of compound **3b**

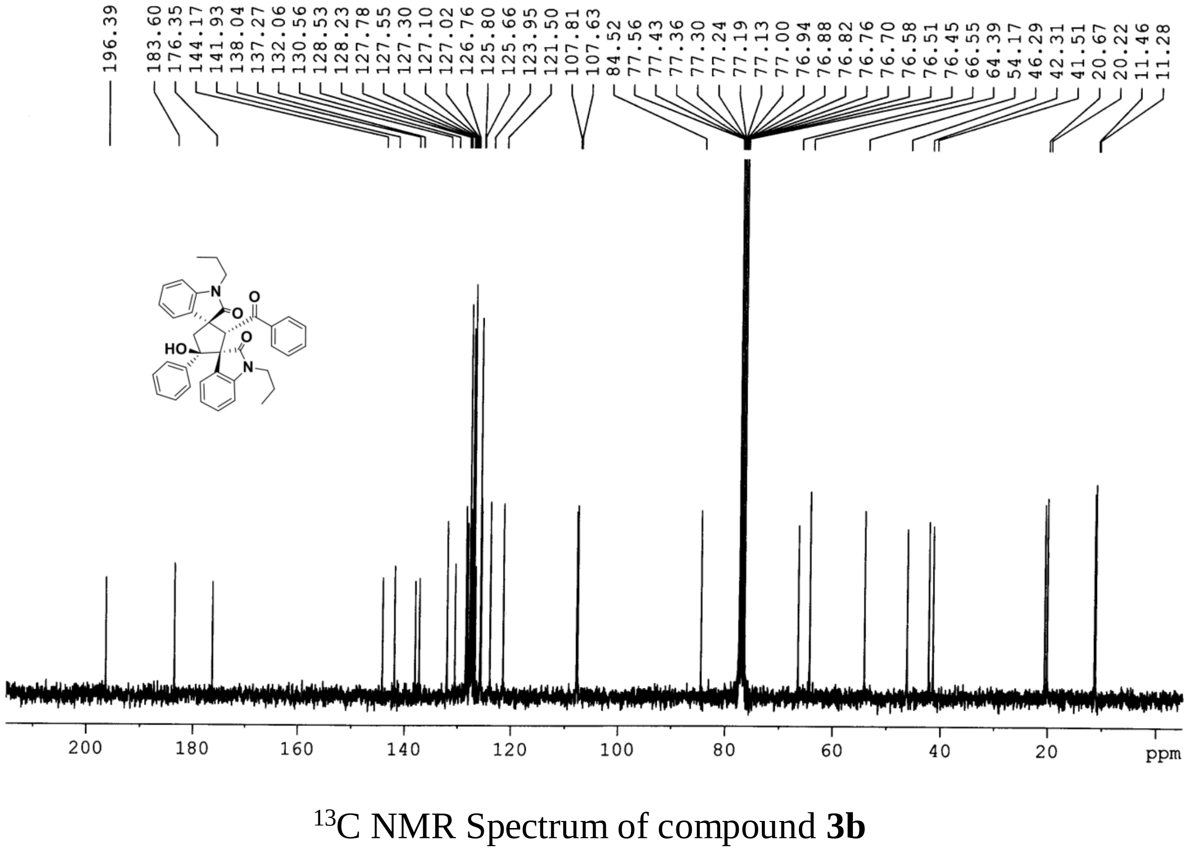

^{13}C NMR Spectrum of compound **3b**

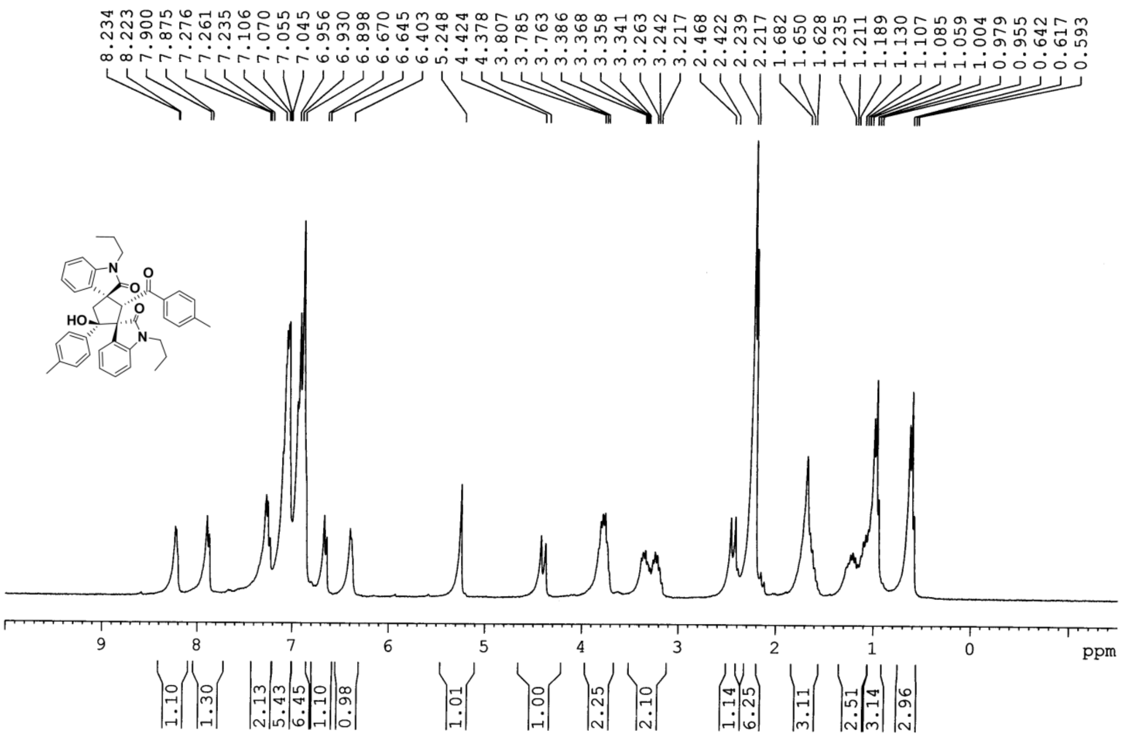

^{1}H NMR Spectrum of compound **3c**

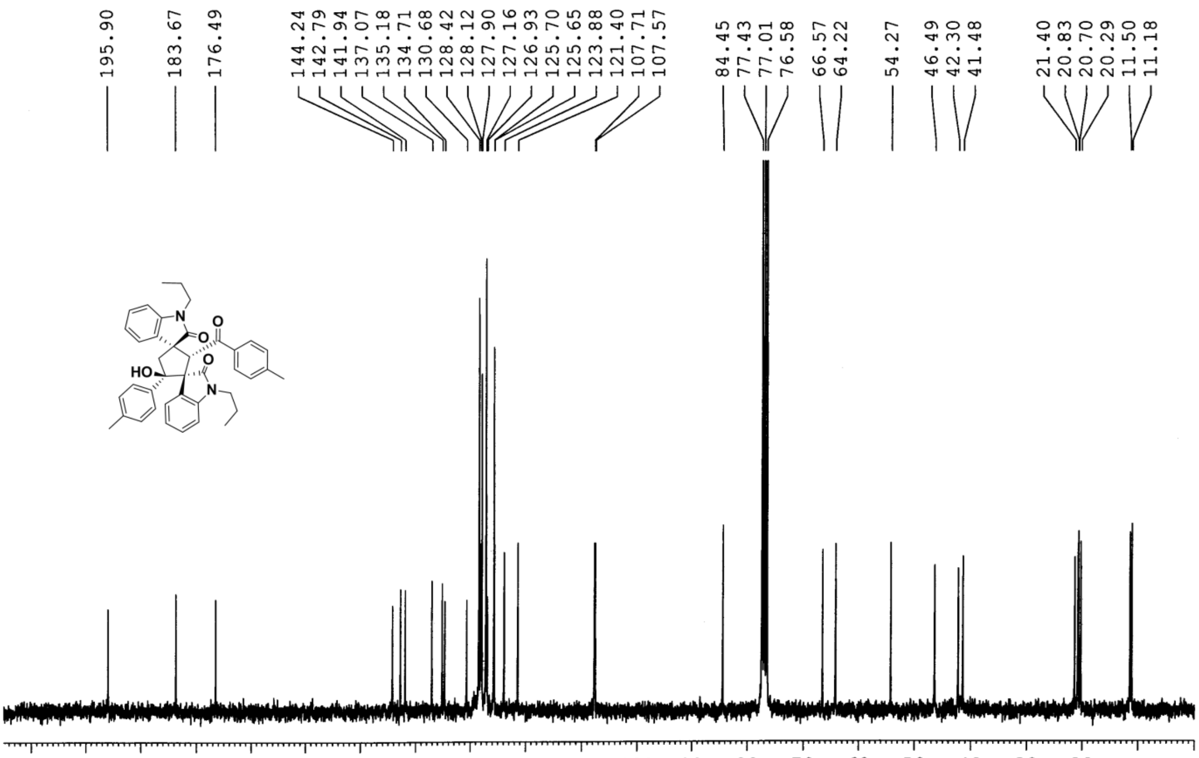

^{13}C NMR Spectrum of compound **3c**

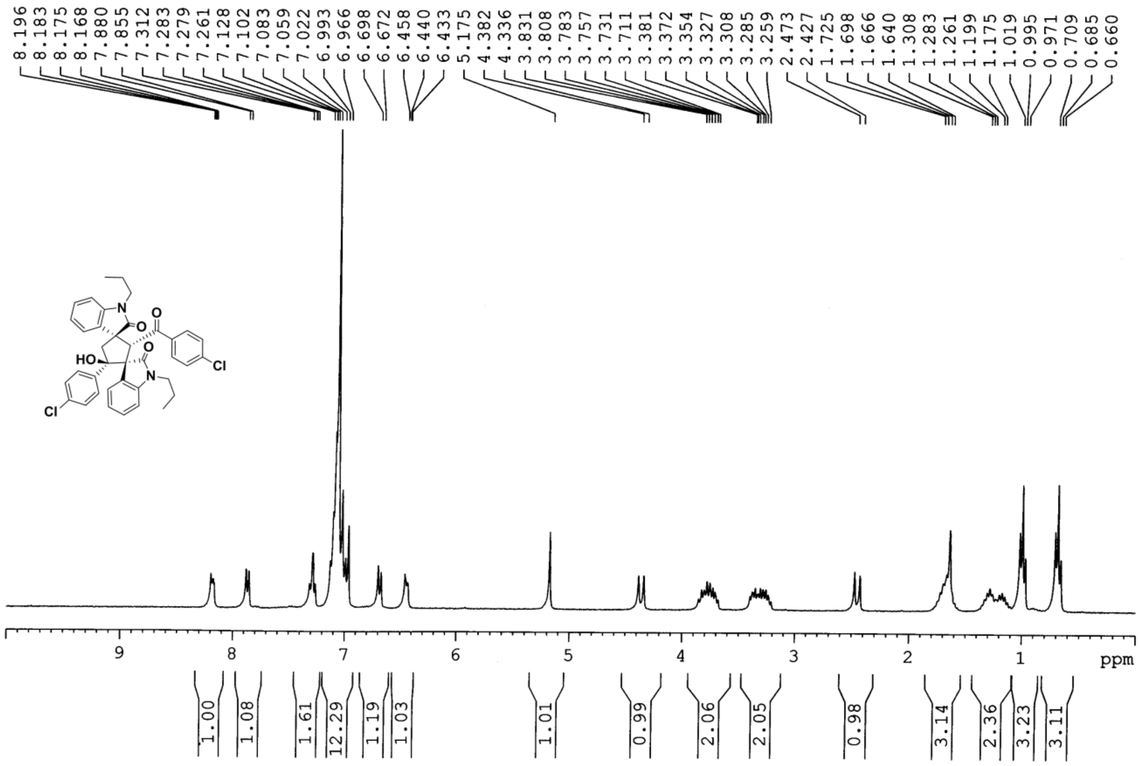

^{1}H NMR Spectrum of compound **3d**

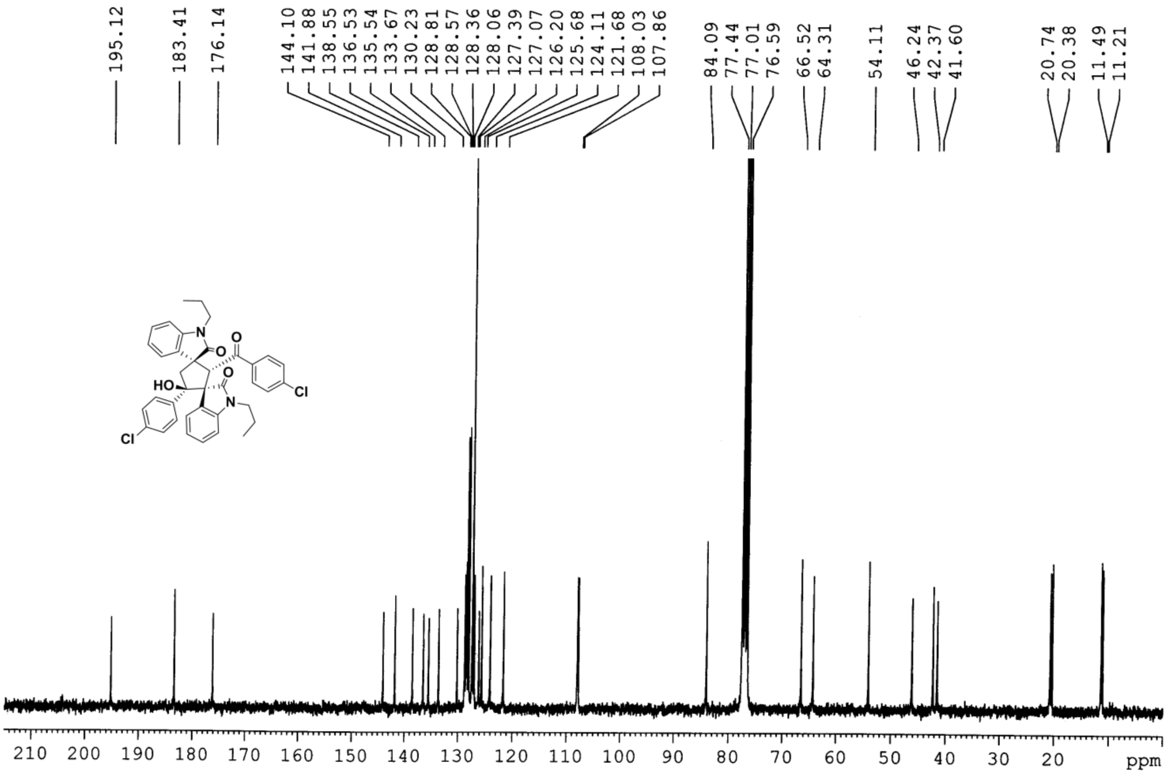

^{13}C NMR Spectrum of compound **3d**

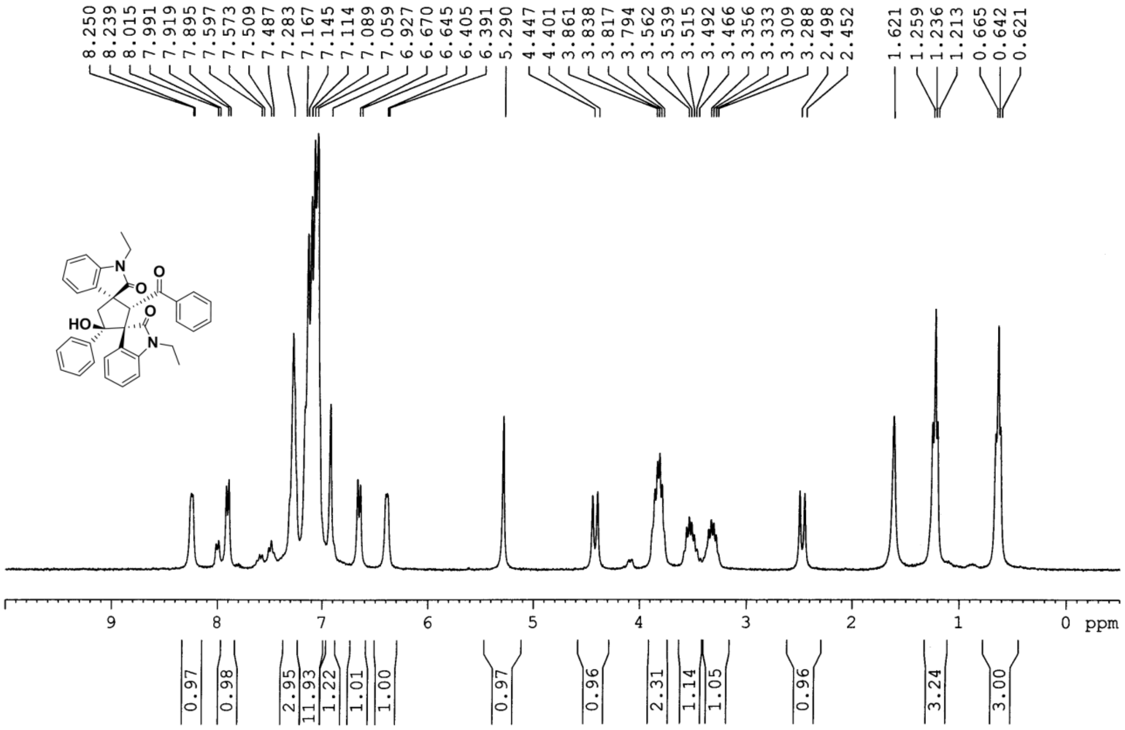

^{1}H NMR Spectrum of compound **3e**

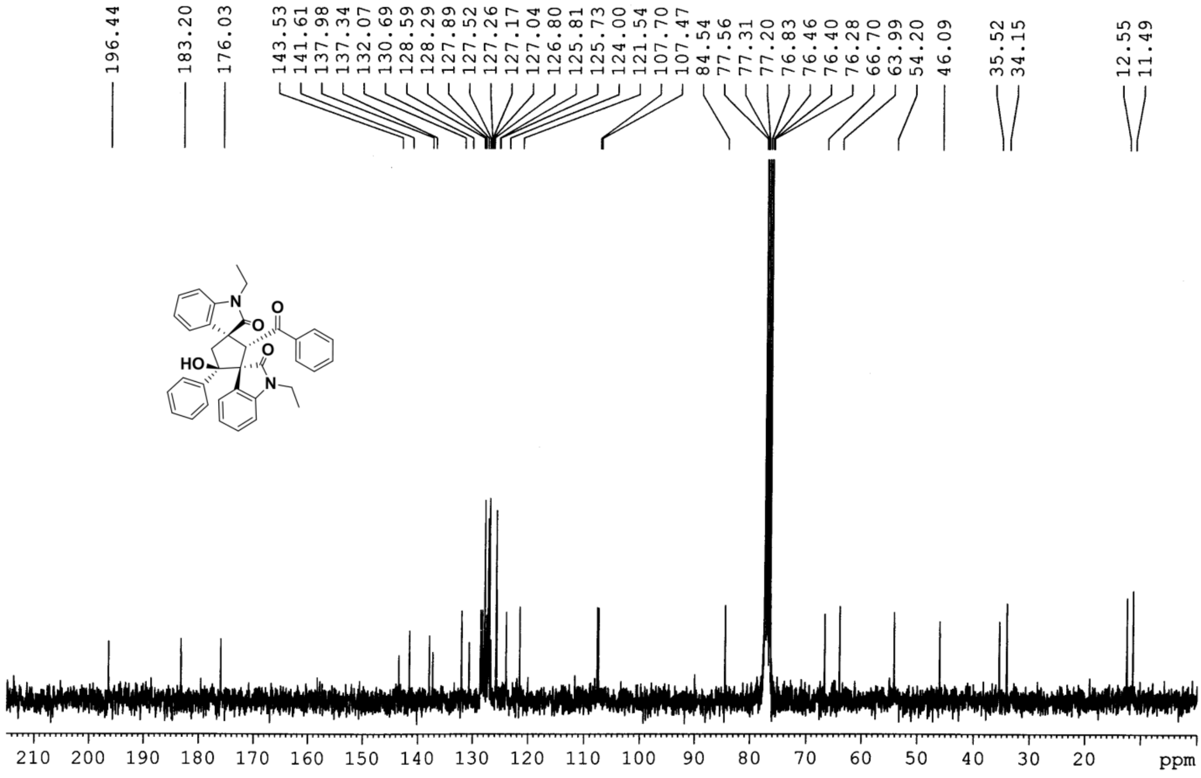

^{13}C NMR Spectrum of compound **3e**

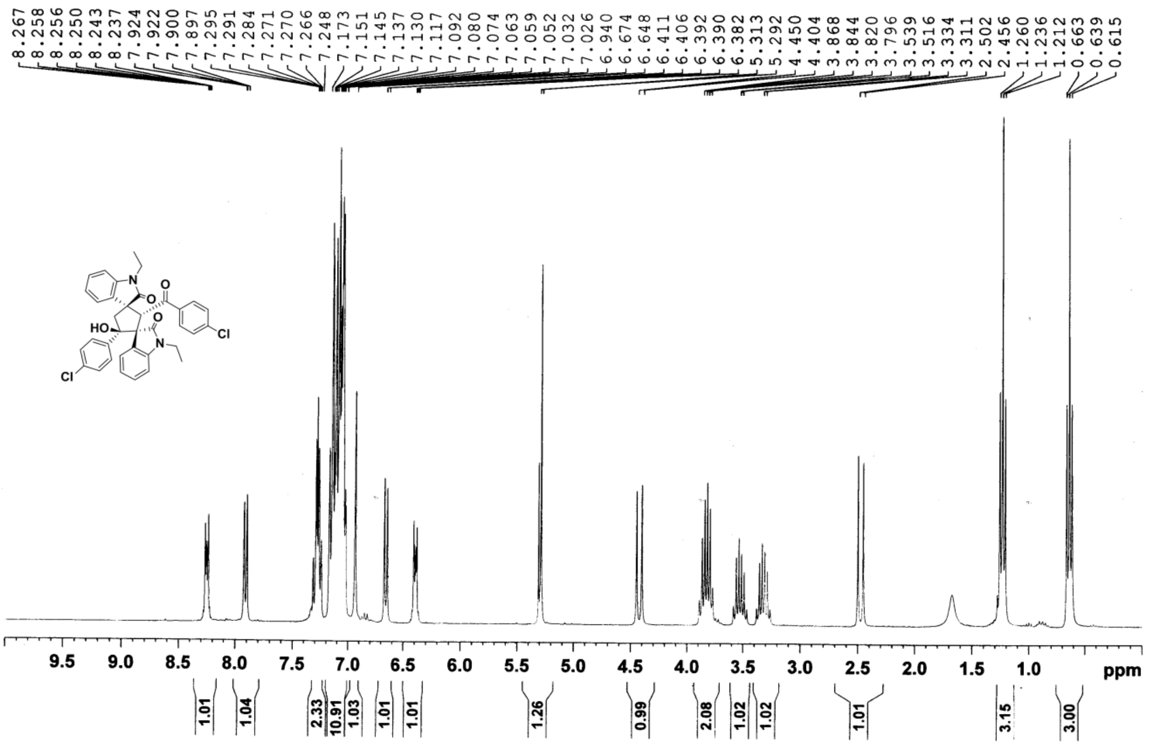

^{1}H NMR Spectrum of compound **3f**

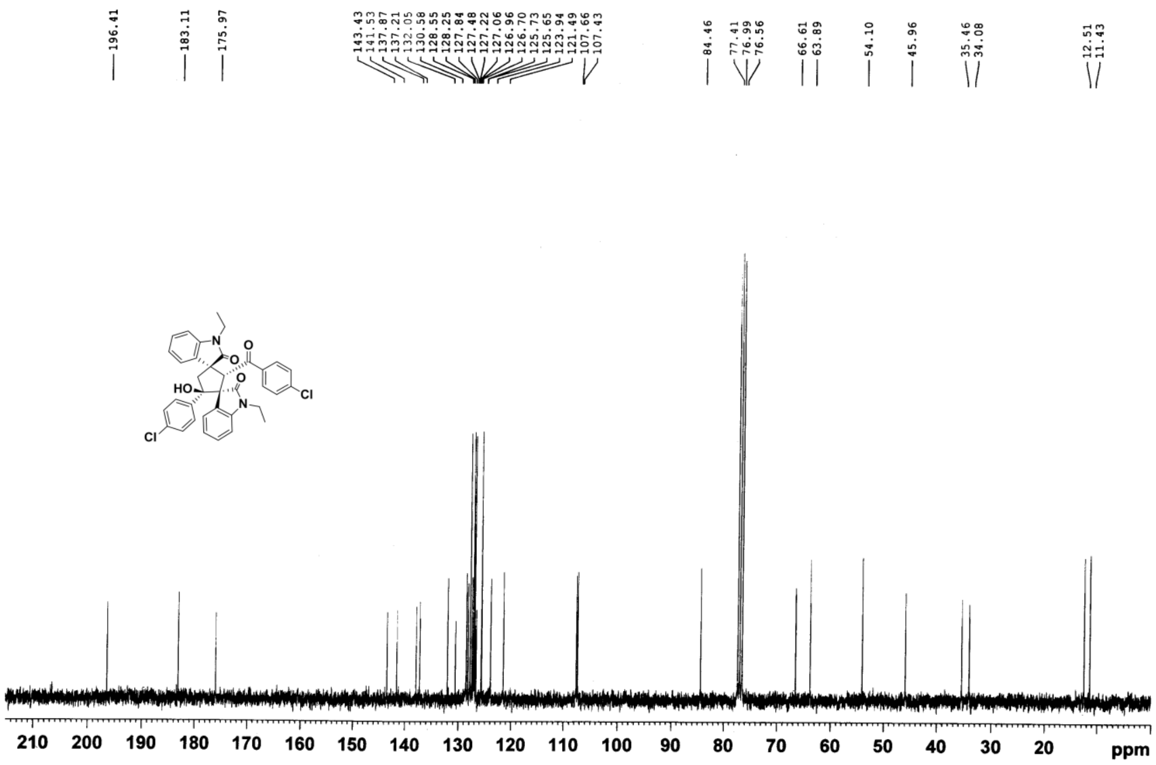

^{13}C NMR Spectrum of compound **3f**

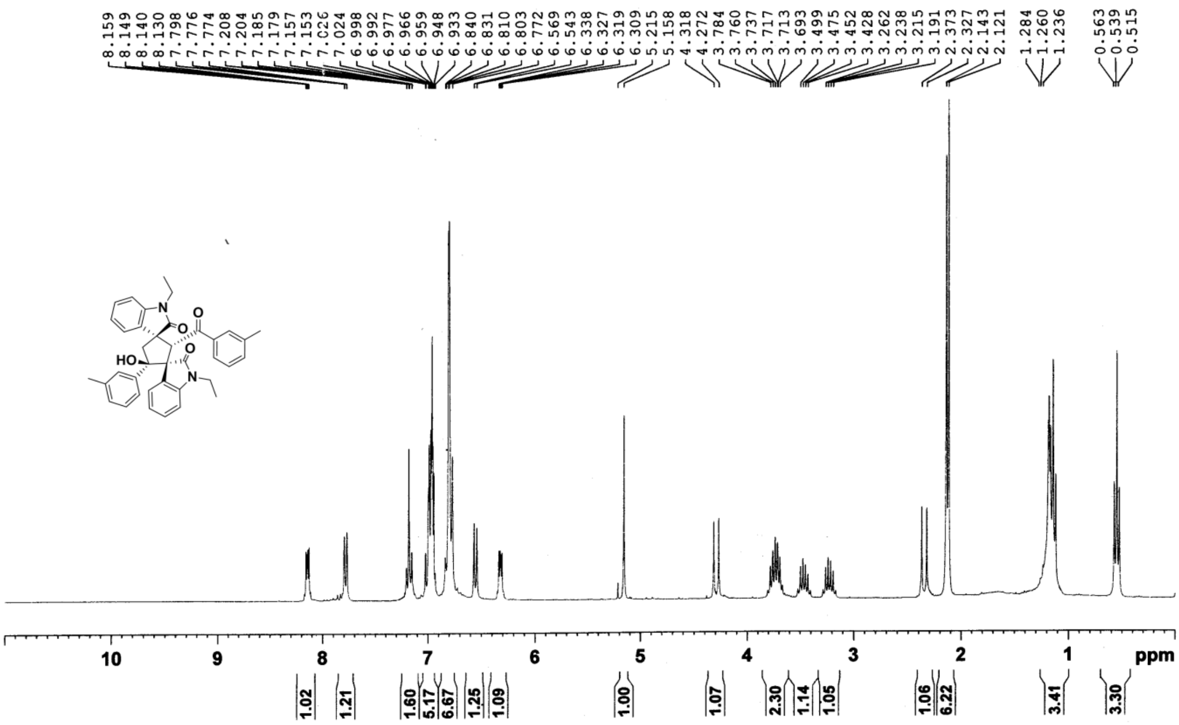

^{1}H NMR Spectrum of compound **3g**

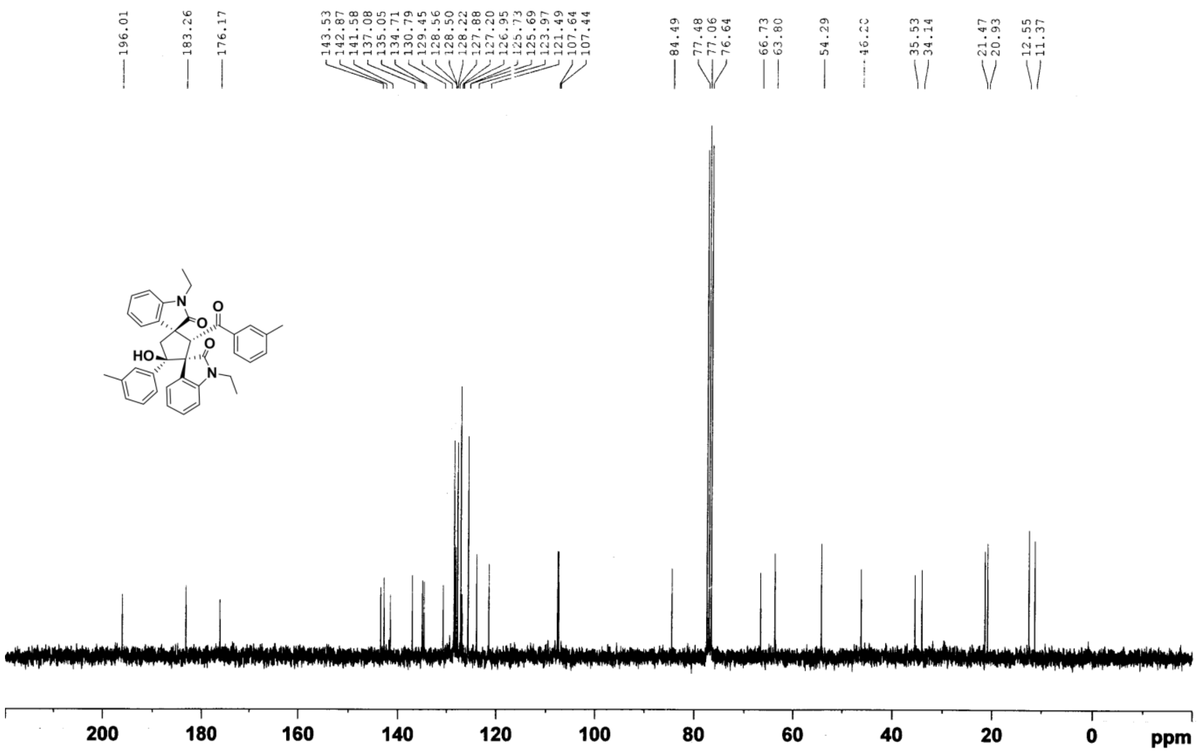

^{13}C NMR Spectrum of compound **3g**

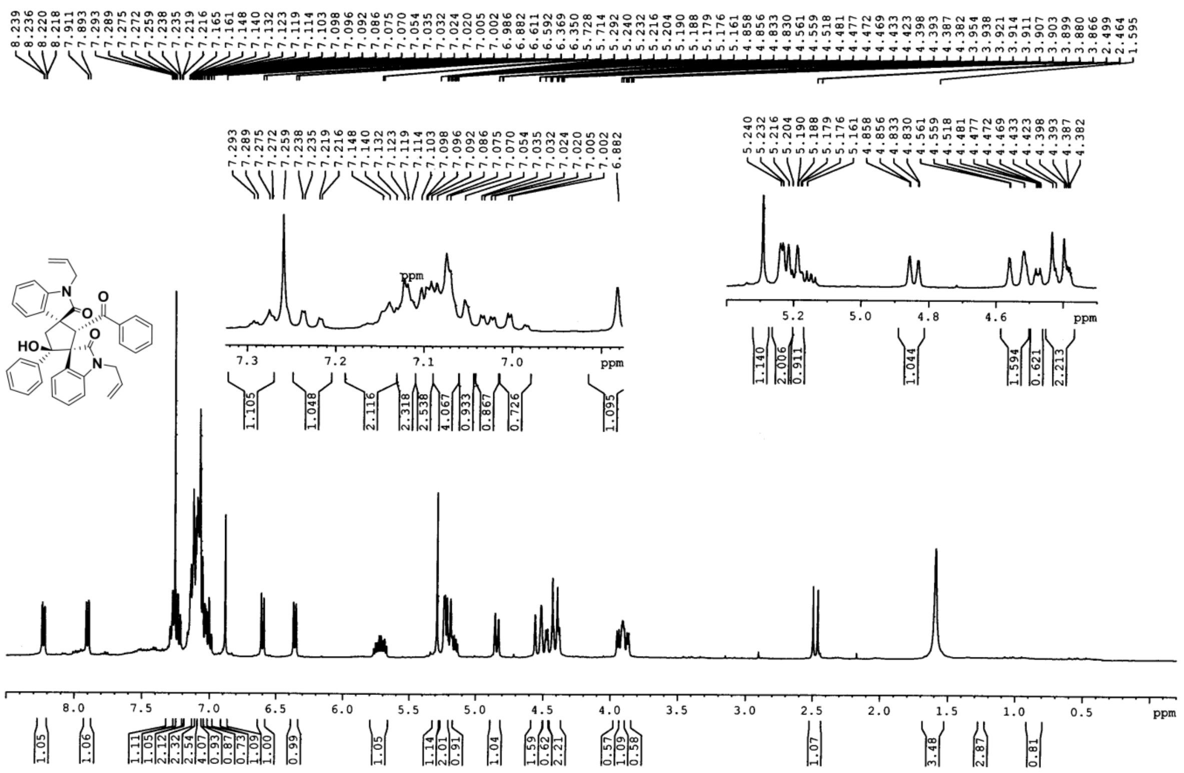

^{1}H NMR Spectrum of compound **3h**

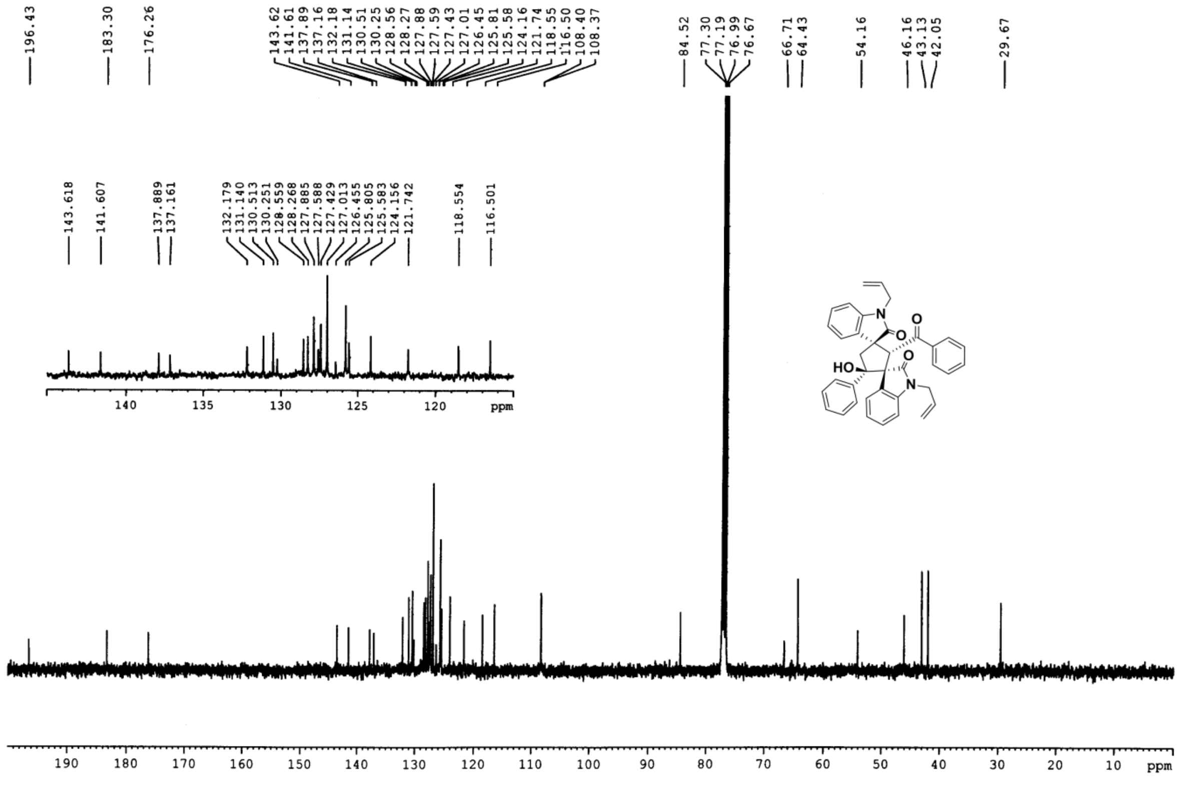

^{13}C NMR Spectrum of compound **3h**

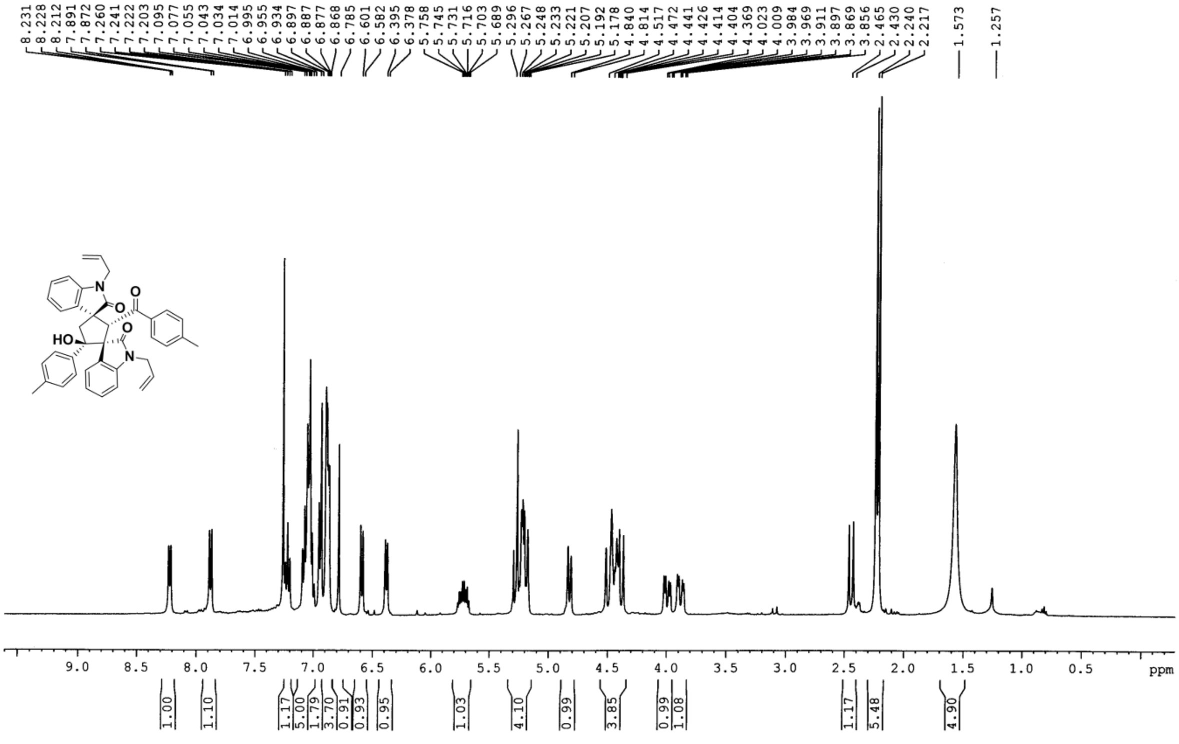

^{1}H NMR Spectrum of compound **3i**

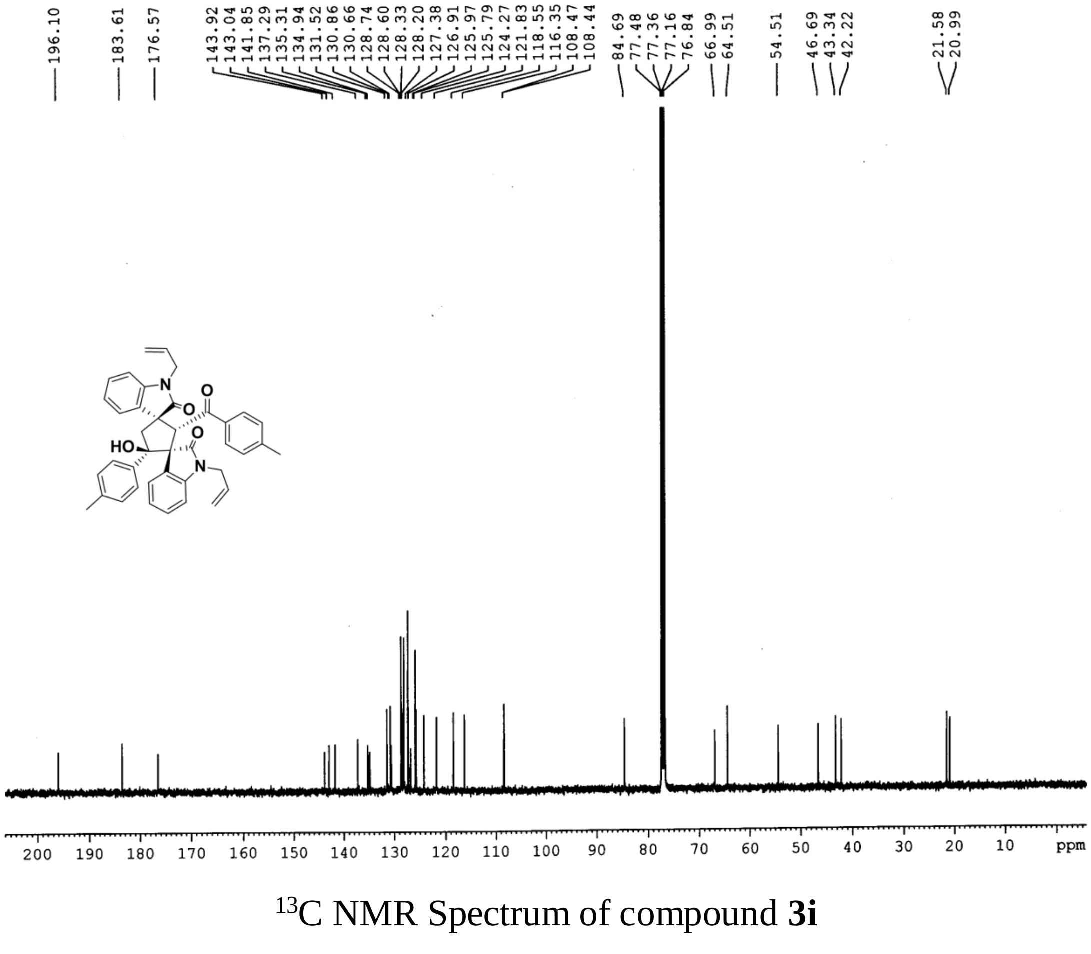

^{13}C NMR Spectrum of compound **3i**

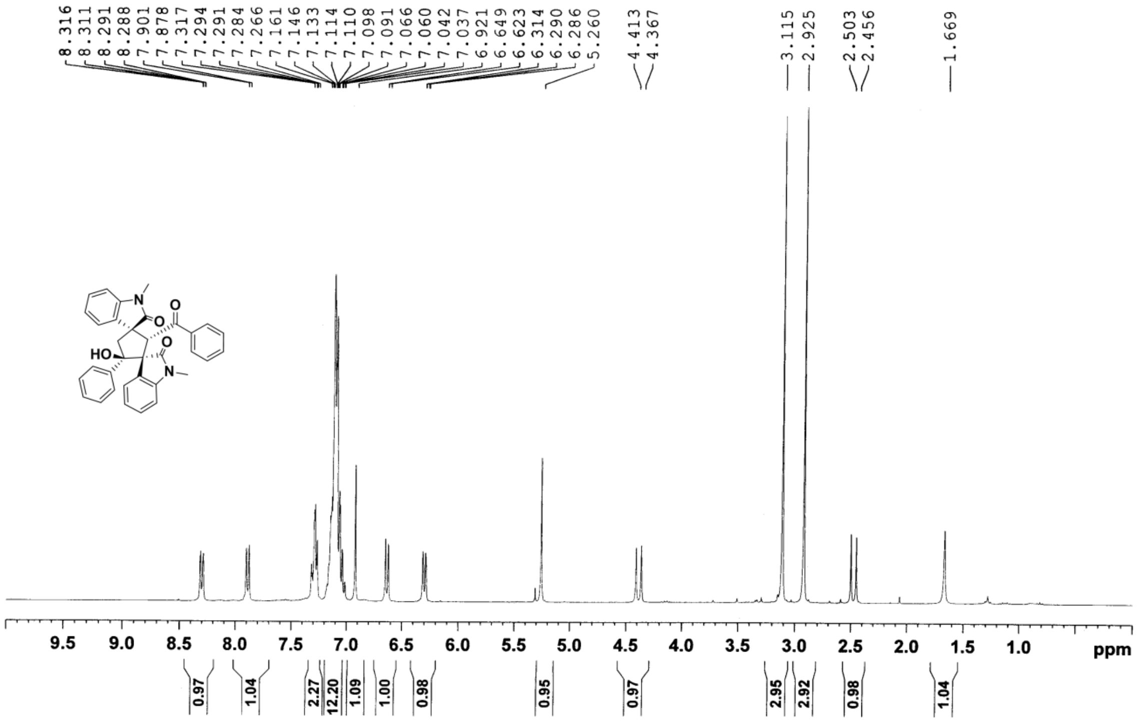

^{1}H NMR Spectrum of compound **3j**

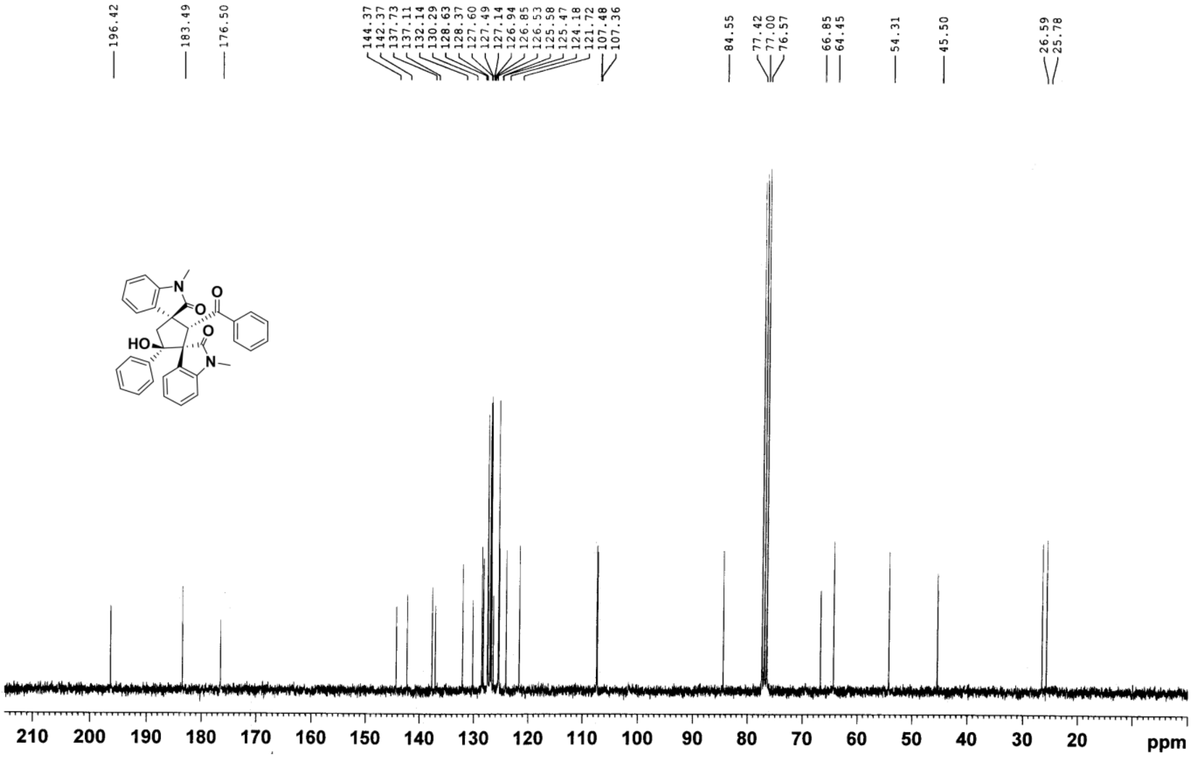

^{13}C NMR Spectrum of compound **3j**

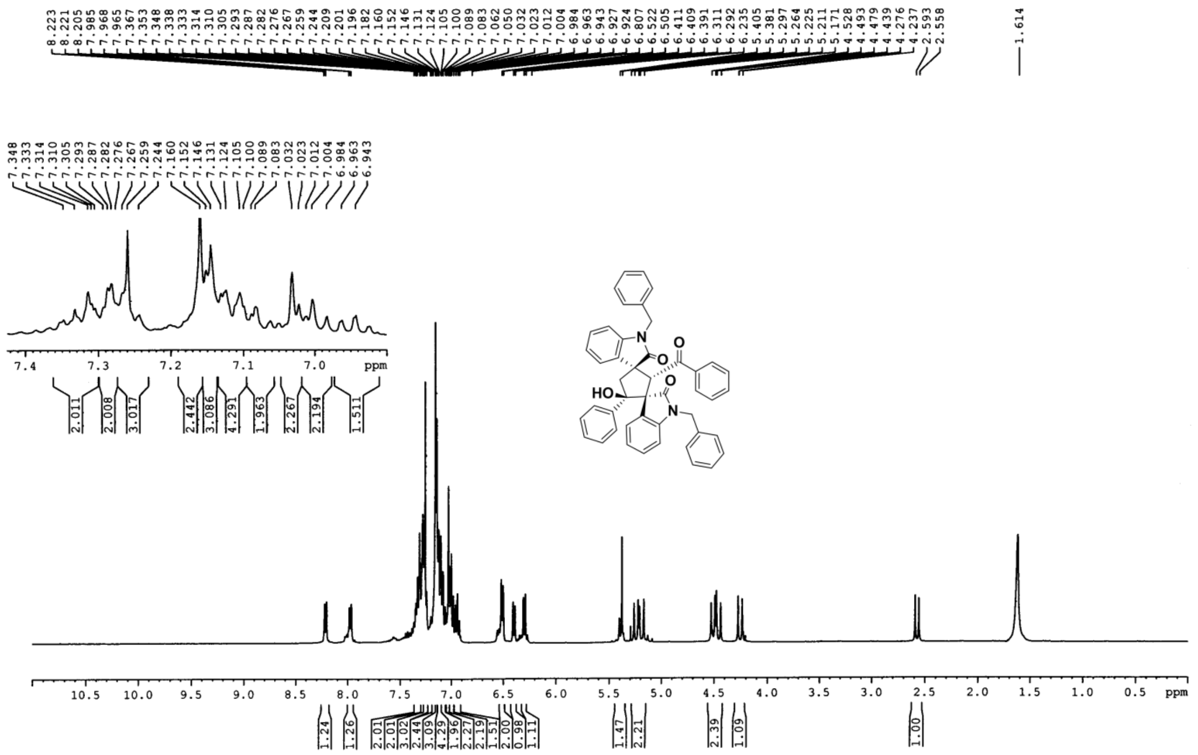

^{1}H NMR Spectrum of compound **3k**

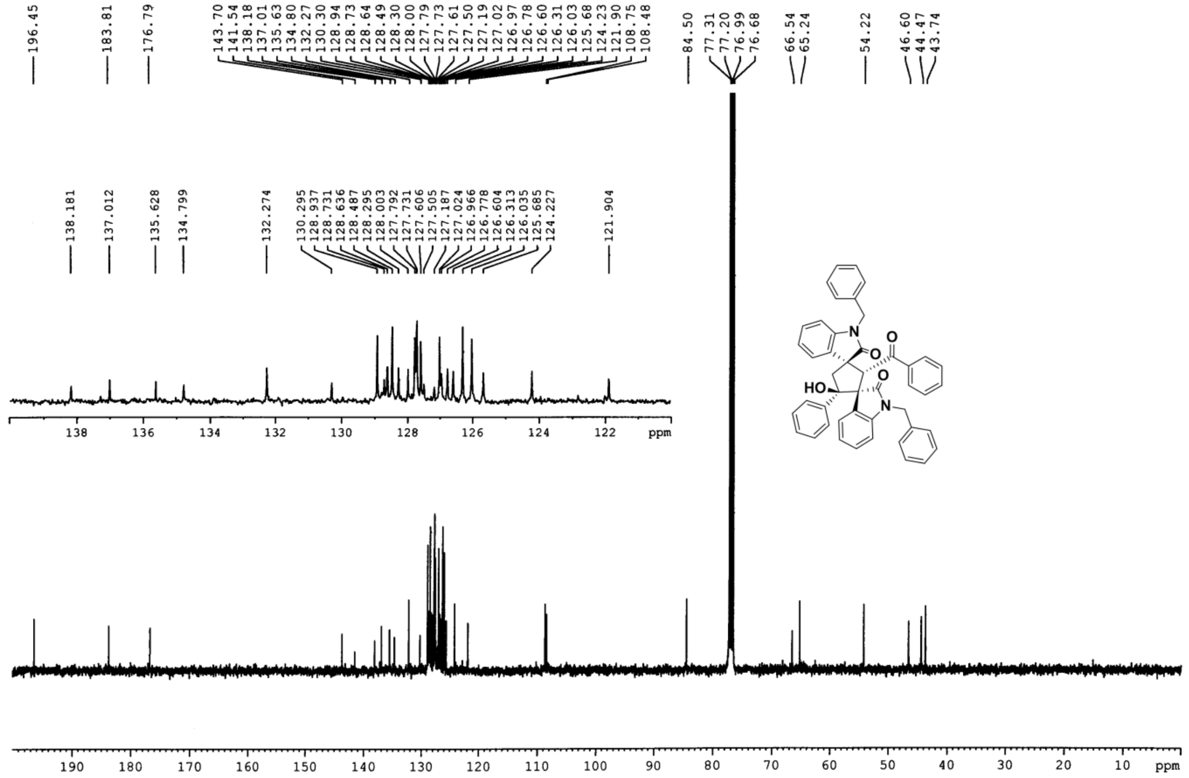

^{13}C NMR Spectrum of compound **3k**

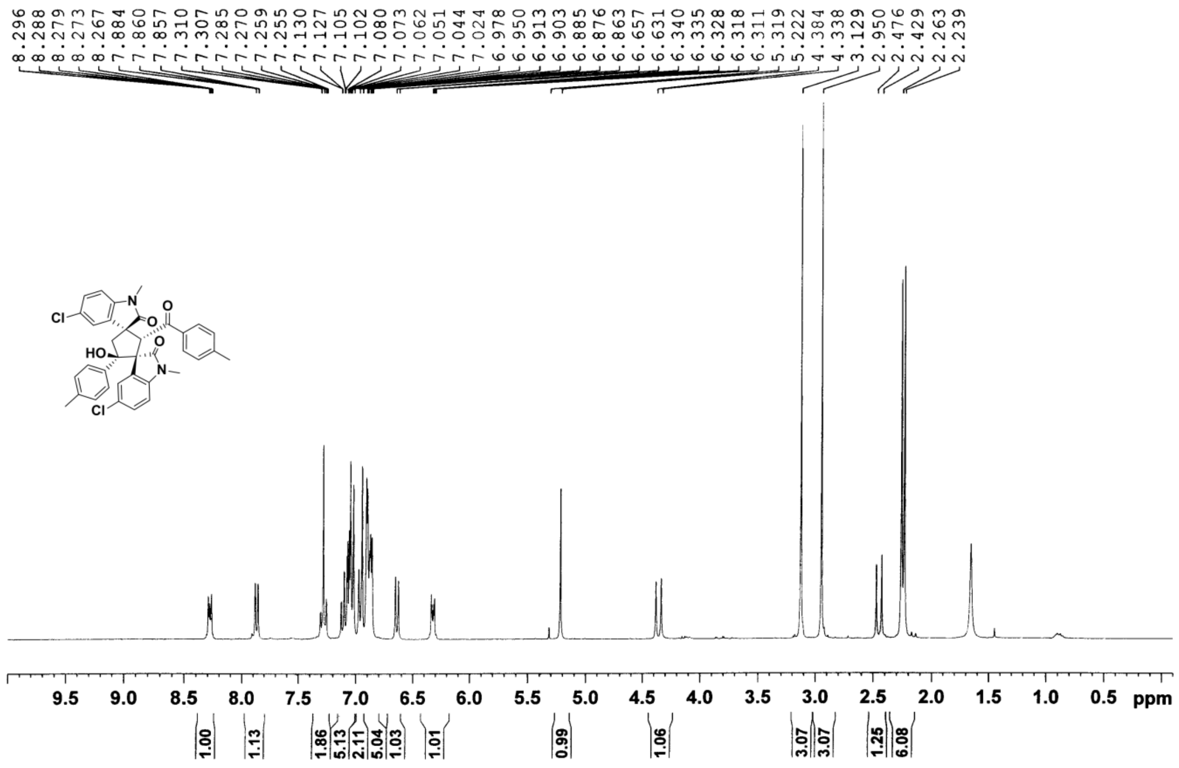

^{1}H NMR Spectrum of compound **3l**

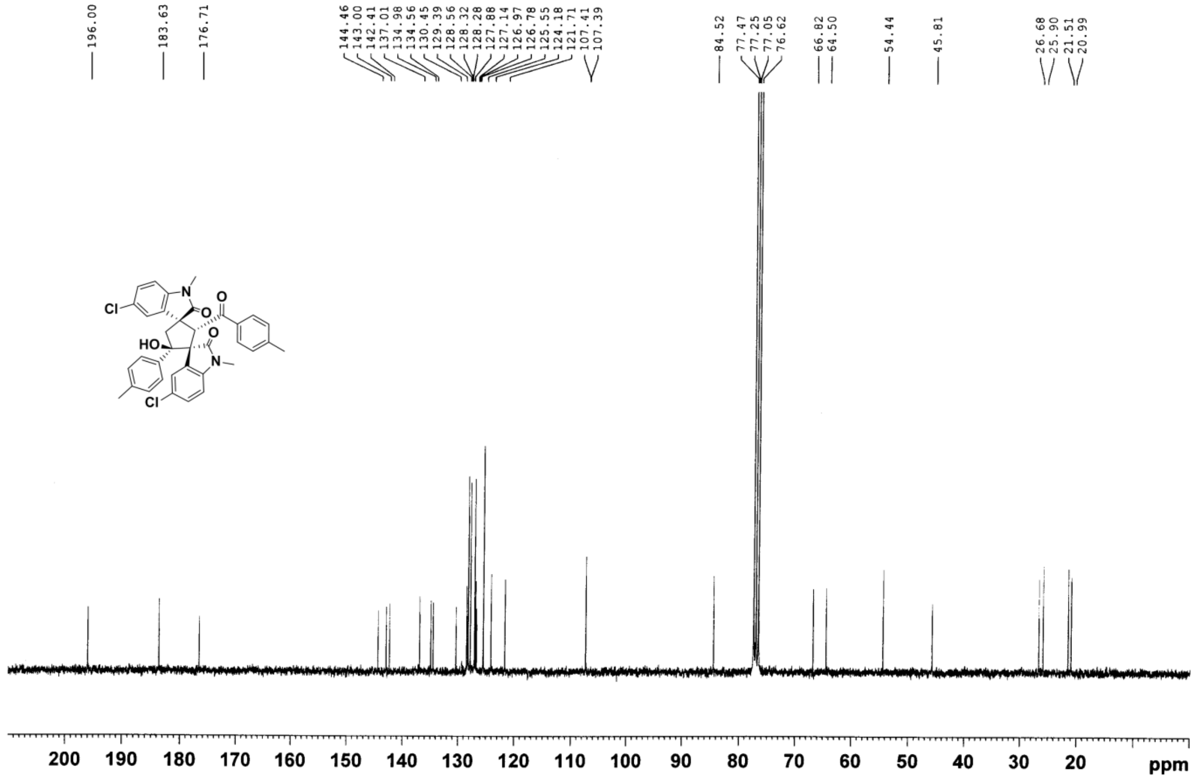

^{13}C NMR Spectrum of compound **3l**

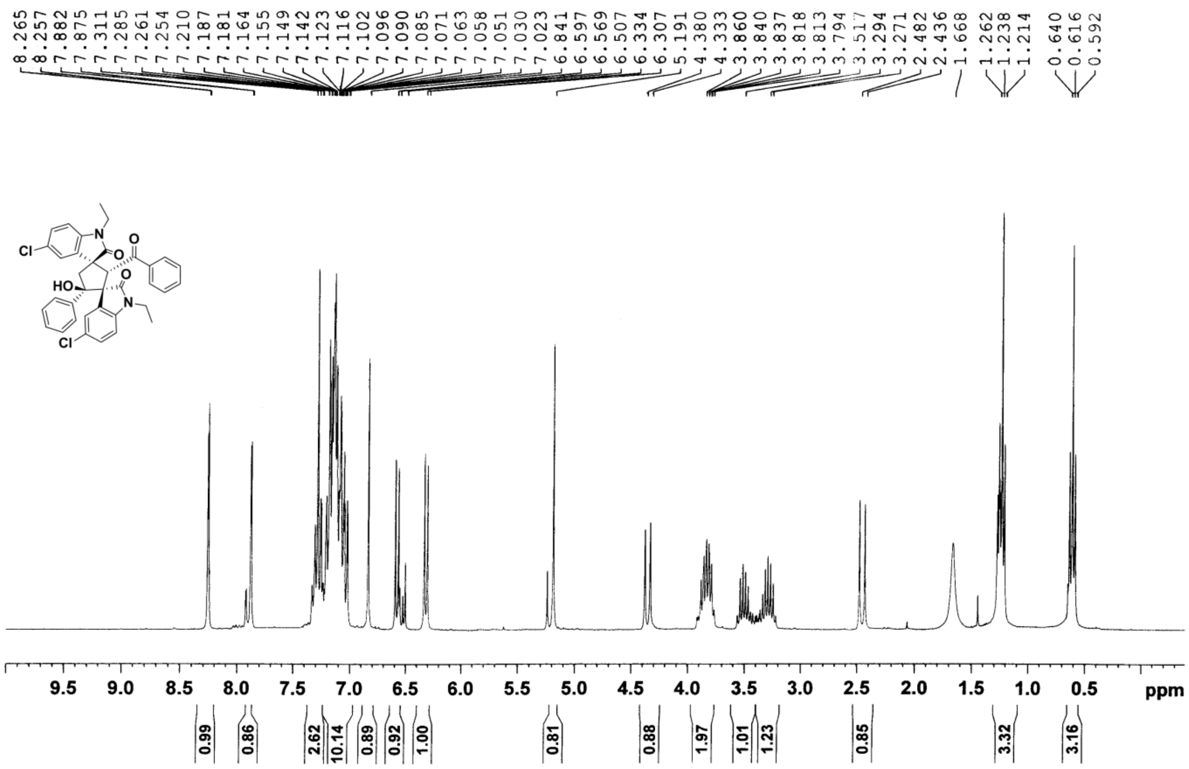

^{1}H NMR Spectrum of compound **3m**

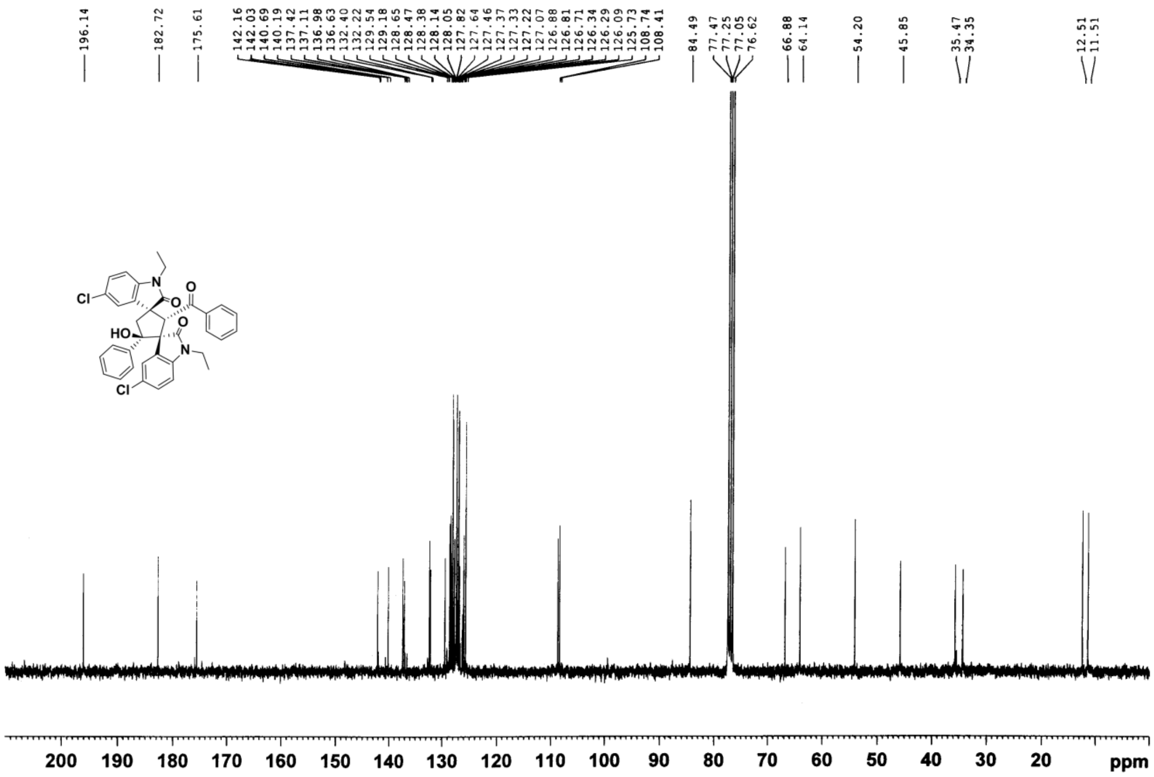

^{13}C NMR Spectrum of compound **3m**

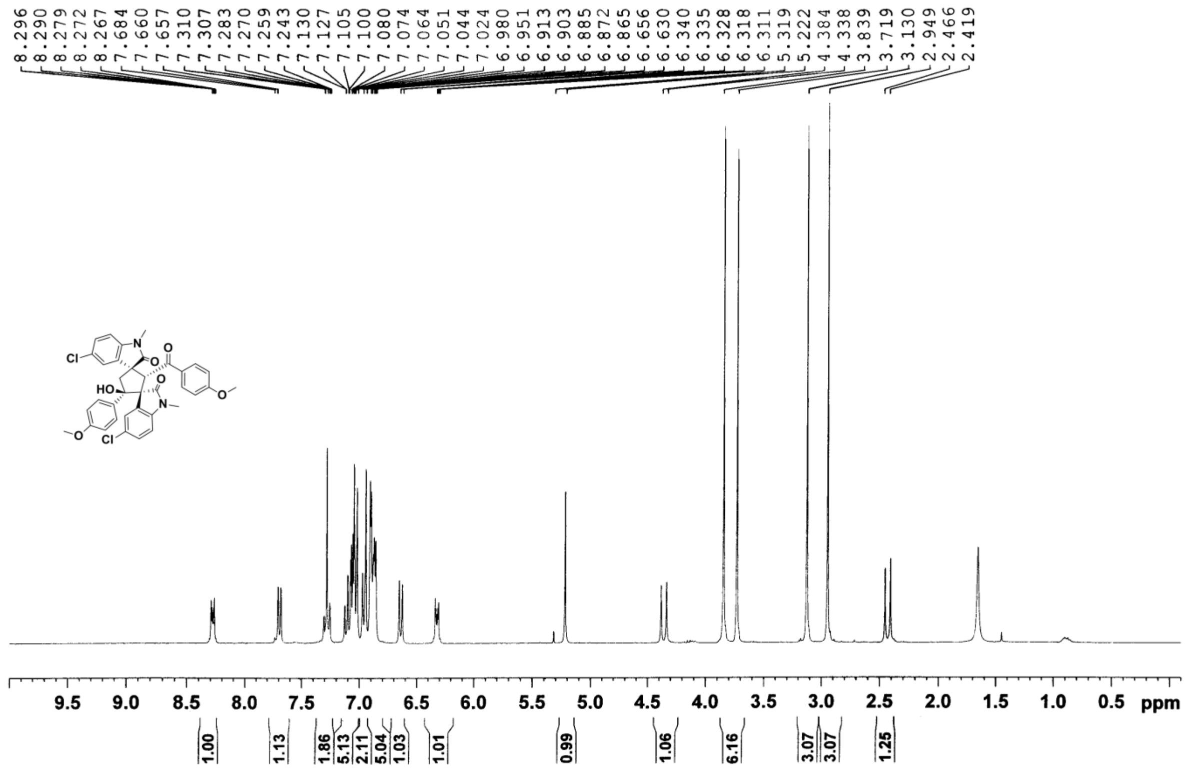

^{1}H NMR Spectrum of compound **3n**

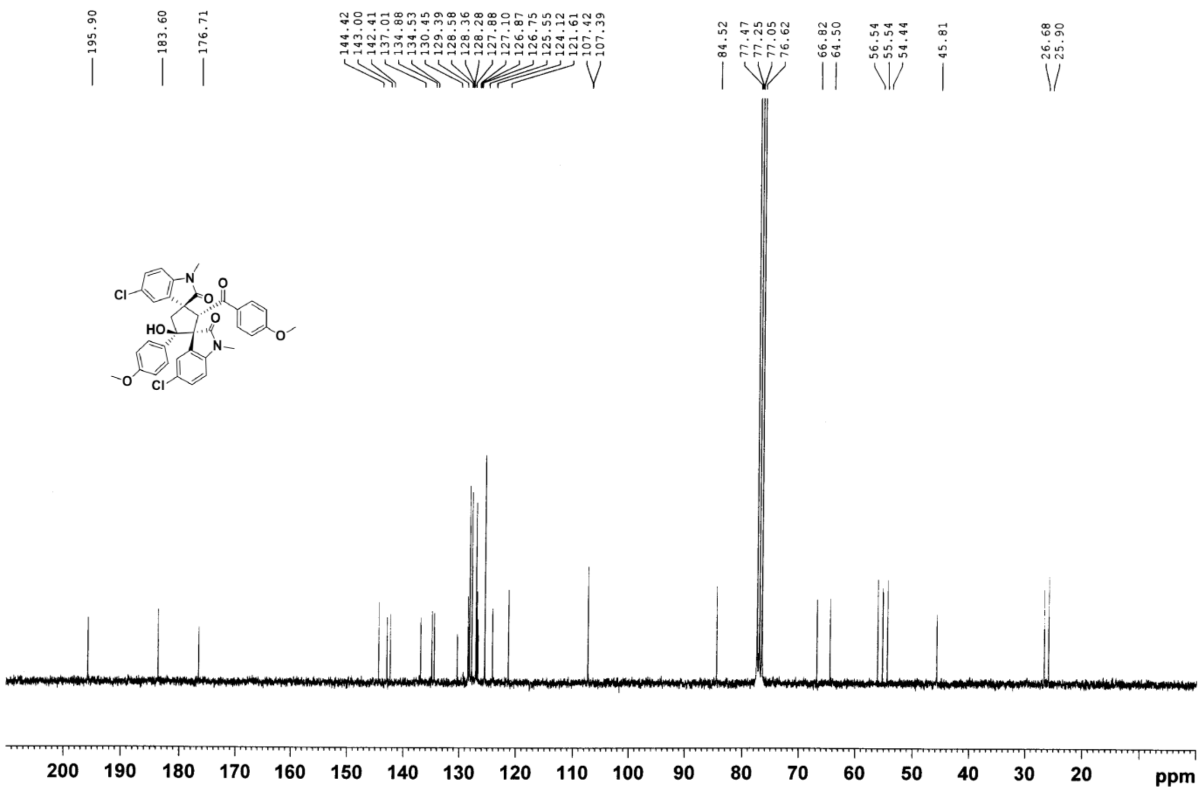

^{13}C NMR Spectrum of compound **3n**

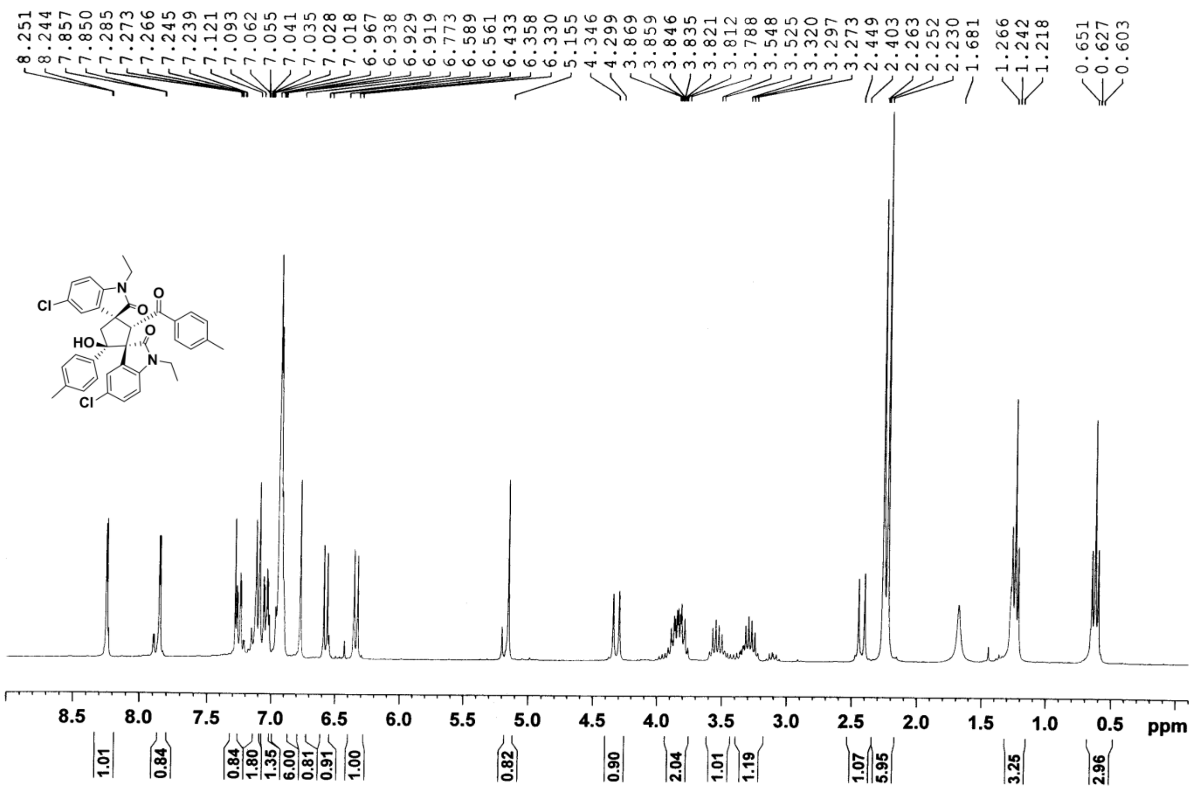

^{1}H NMR Spectrum of compound **3o**

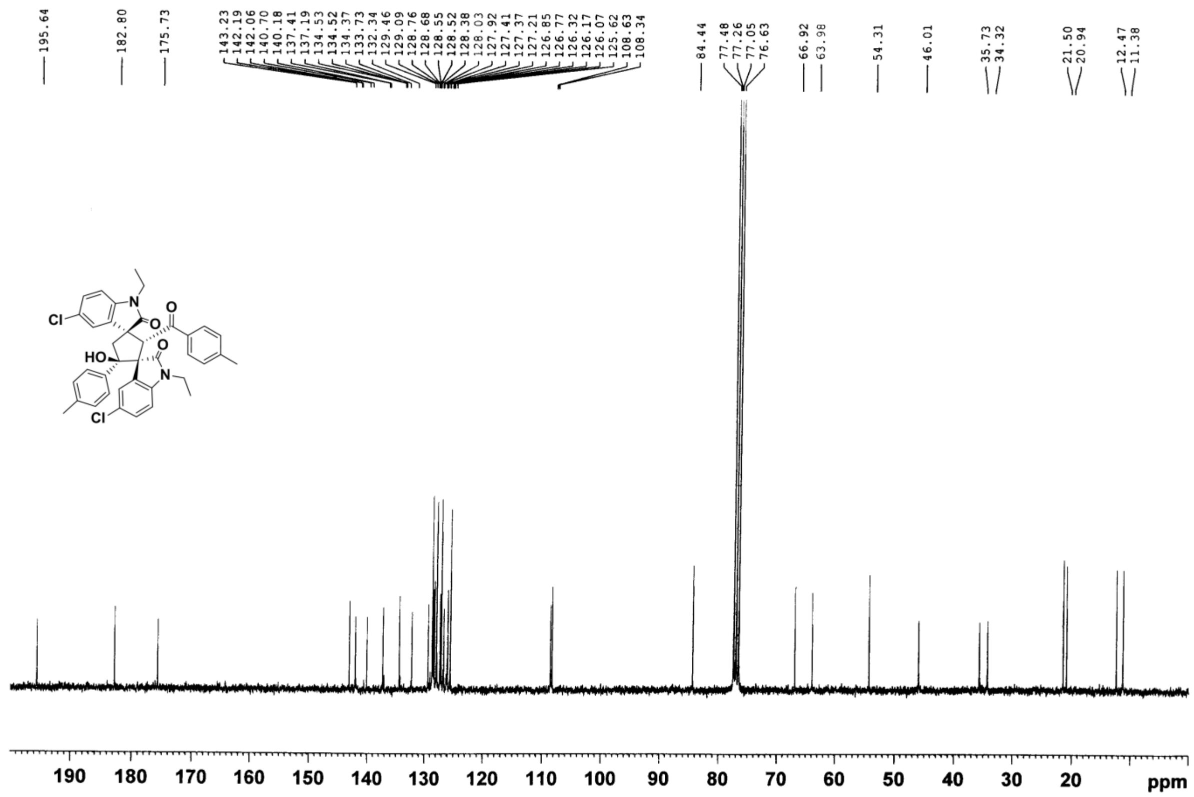

^{13}C NMR Spectrum of compound **3o**

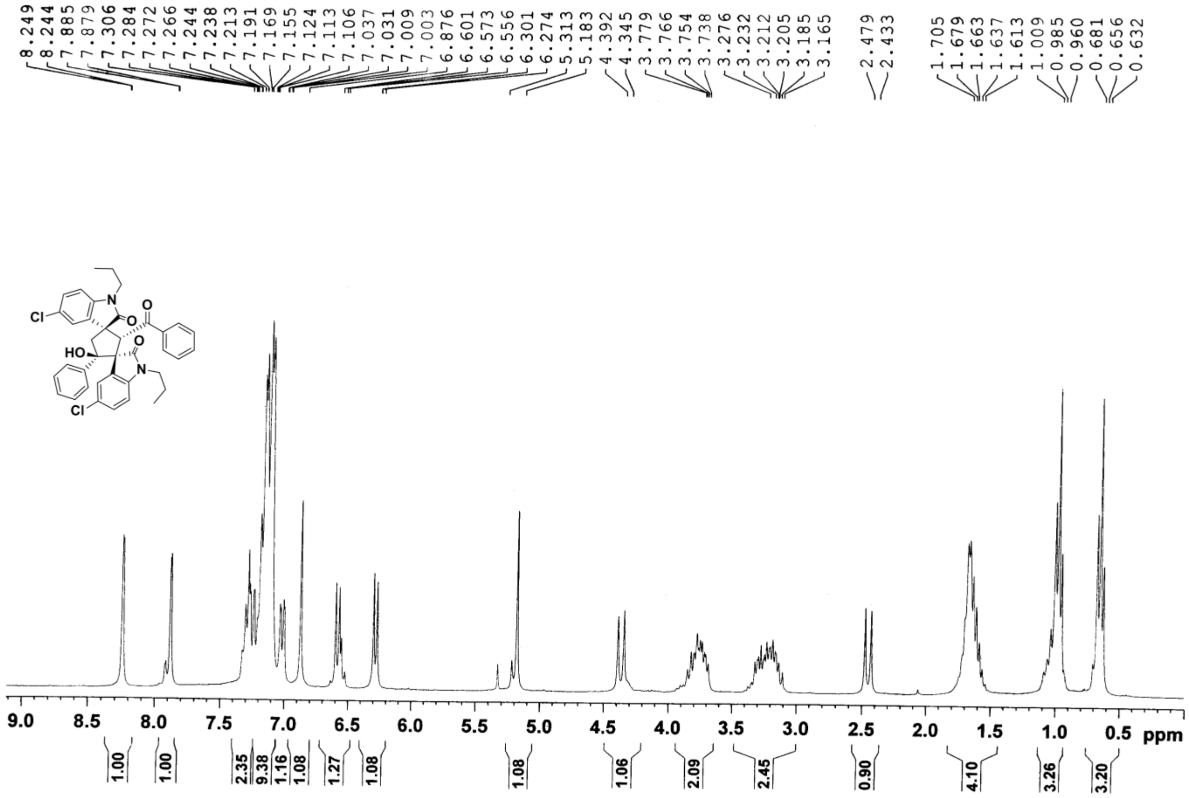

^{1}H NMR Spectrum of compound **3p**

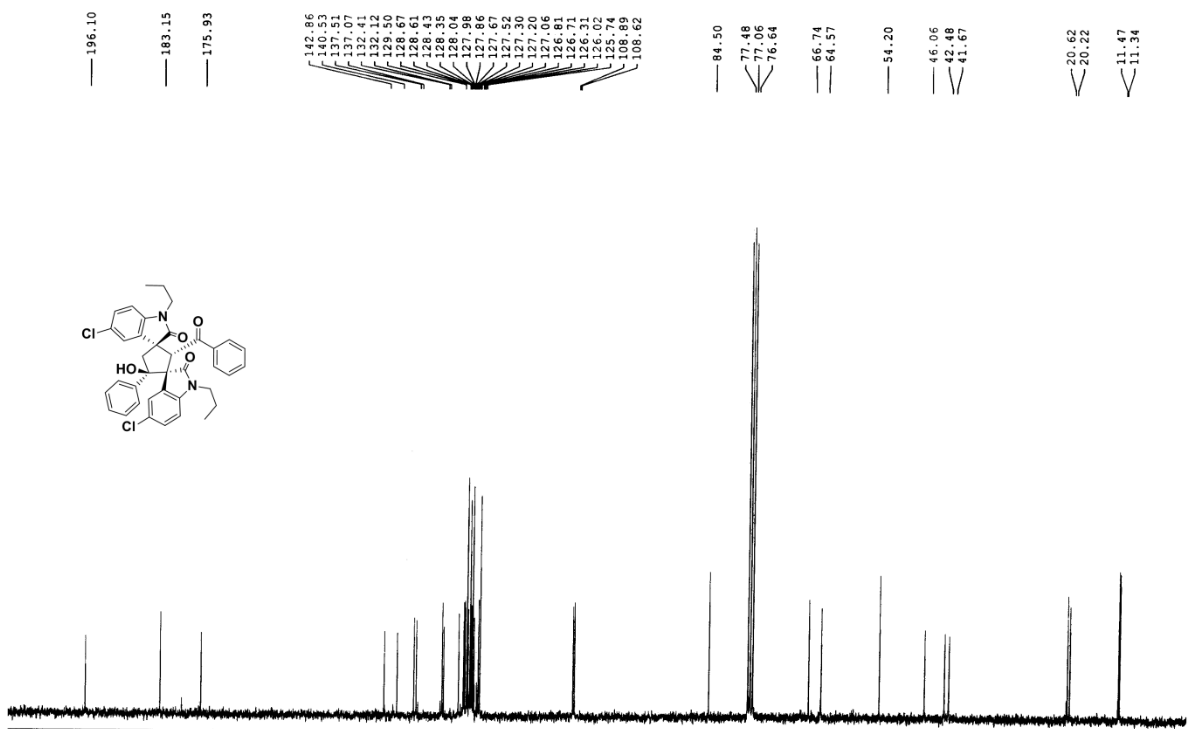

^{13}C NMR Spectrum of compound **3p**

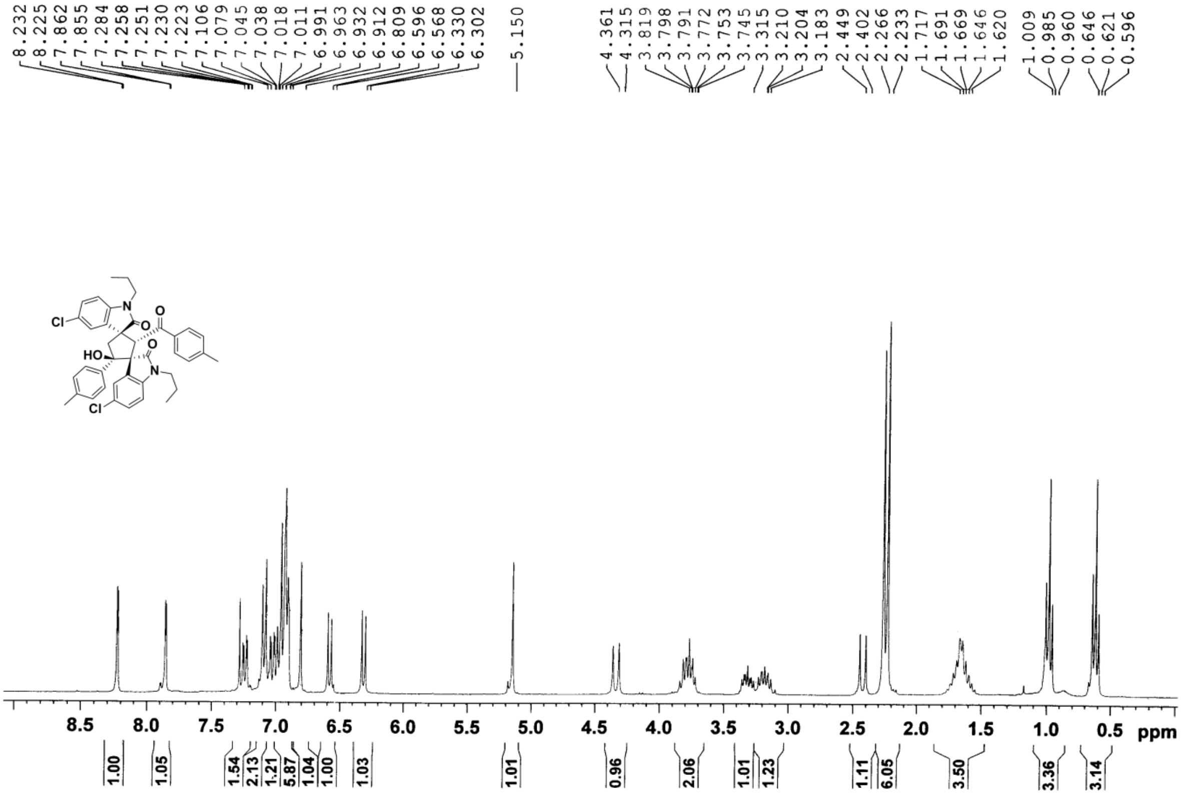

^{1}H NMR Spectrum of compound **3q**

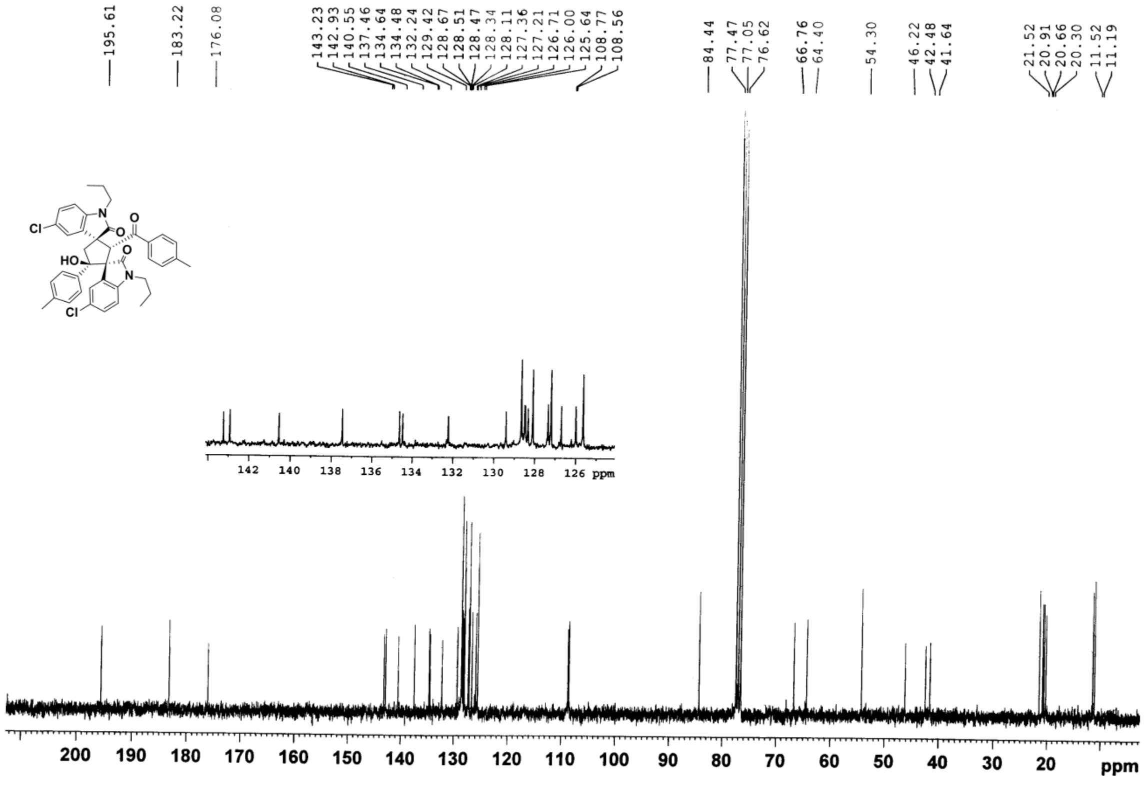

^{13}C NMR Spectrum of compound **3q**

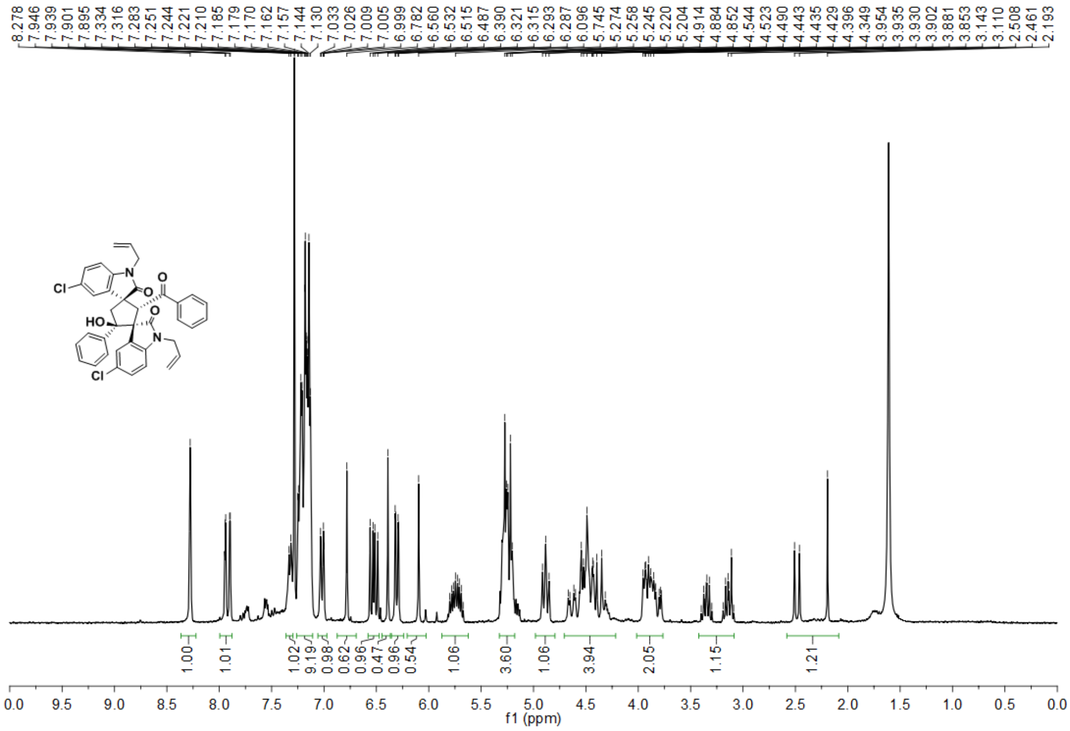

^{1}H NMR Spectrum of compound **3r**

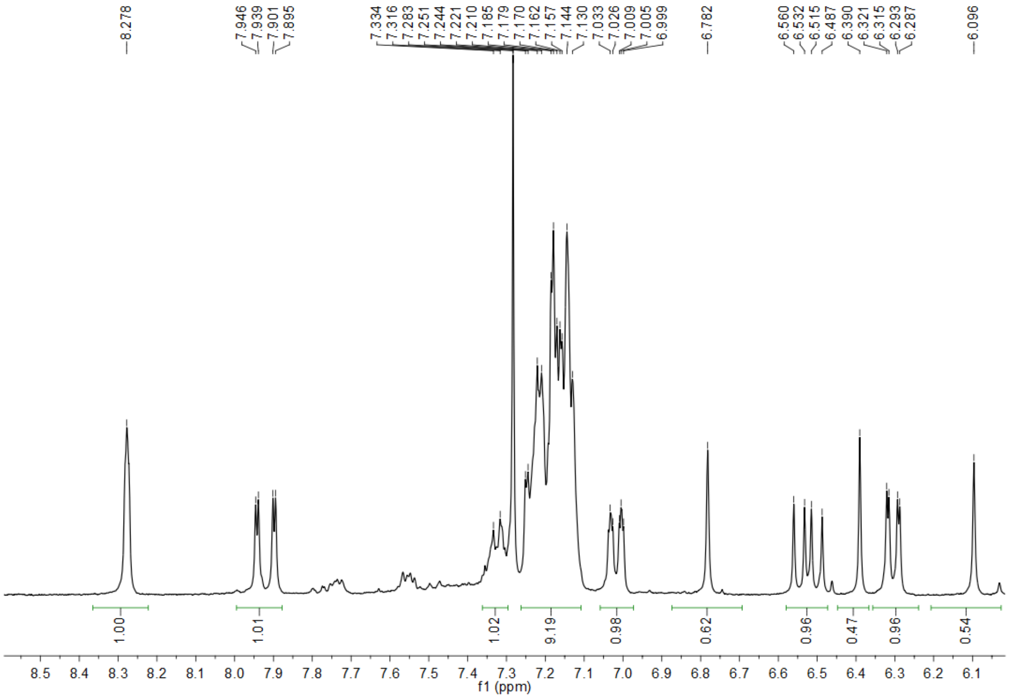

^{1}H NMR Spectrum of compound **3r (Spread)**

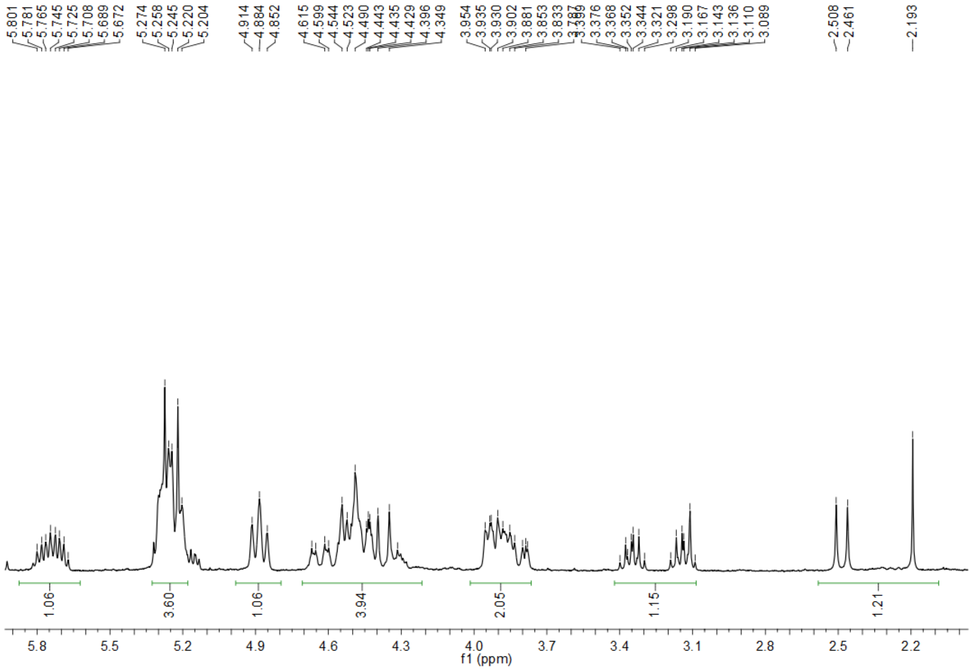

^{1}H NMR Spectrum of compound **3r (Spread)**

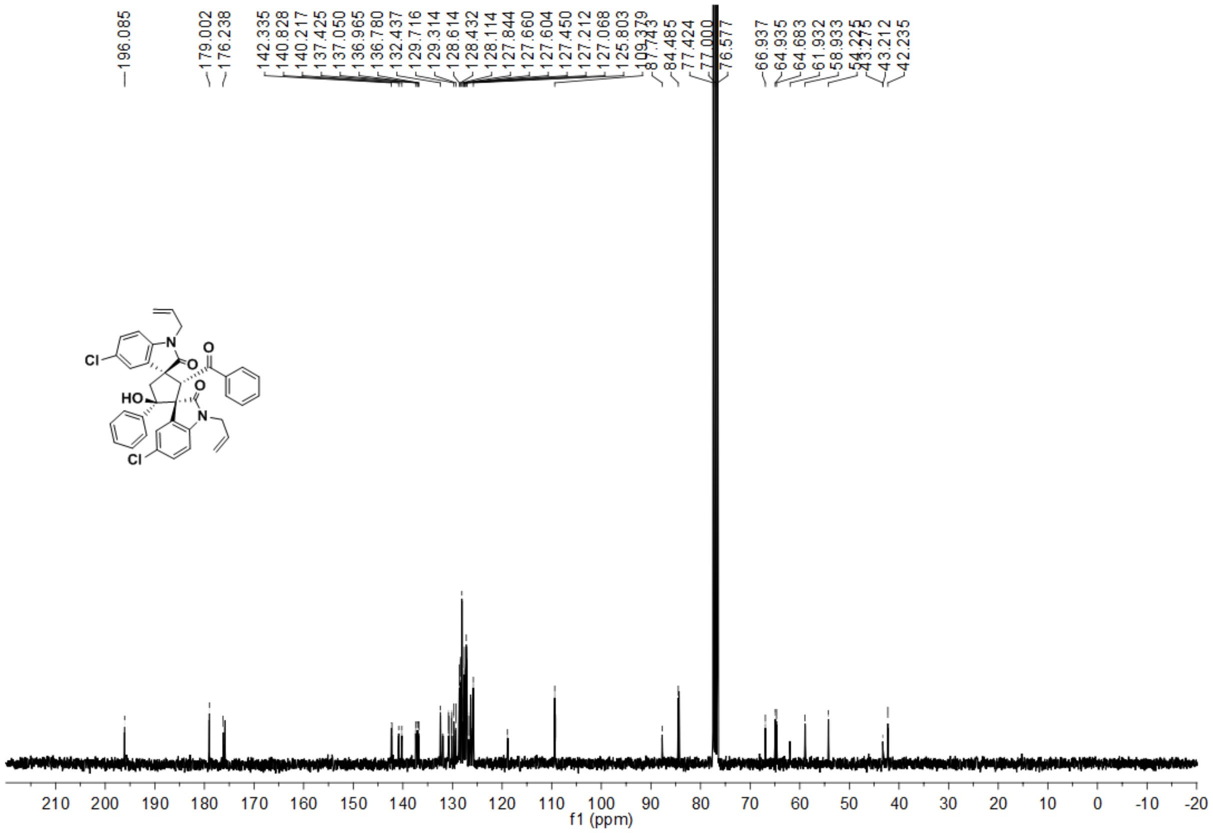

^{13}C NMR Spectrum of compound **3r**

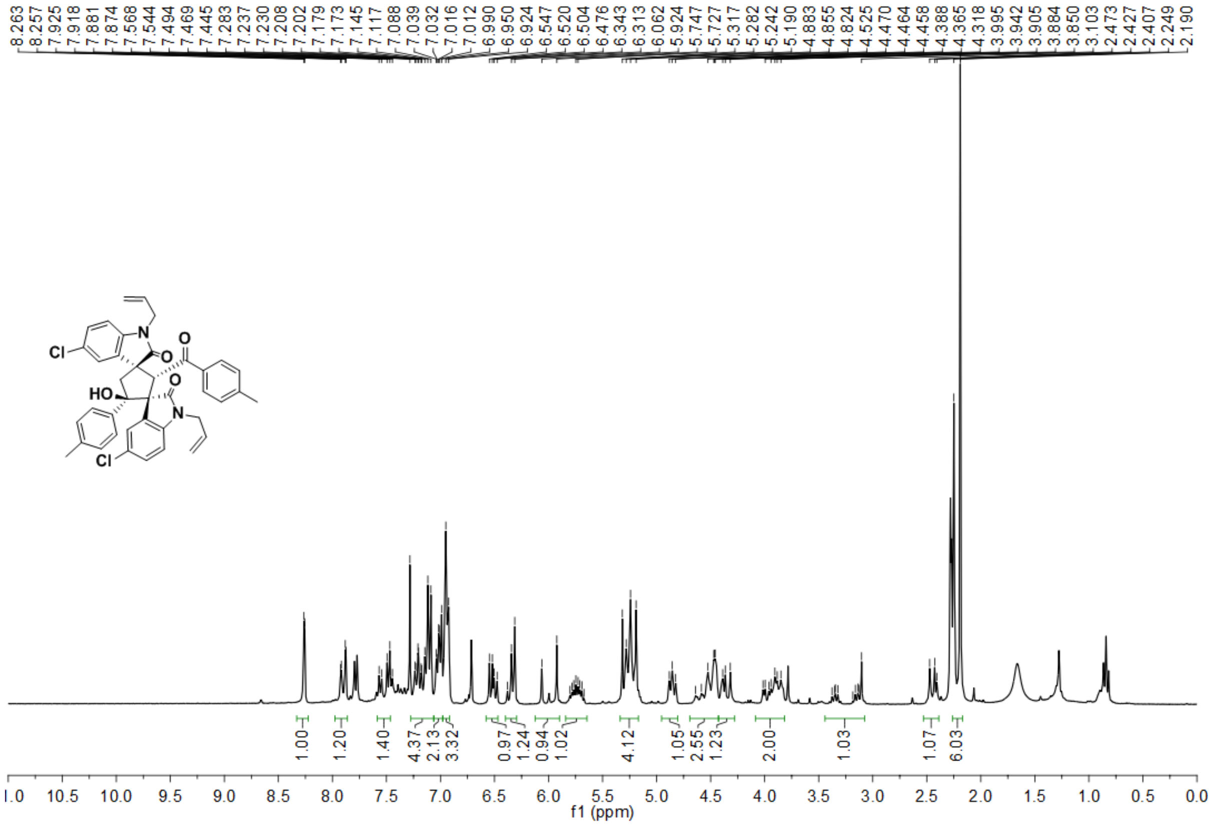

^{1}H NMR Spectrum of compound **3s**

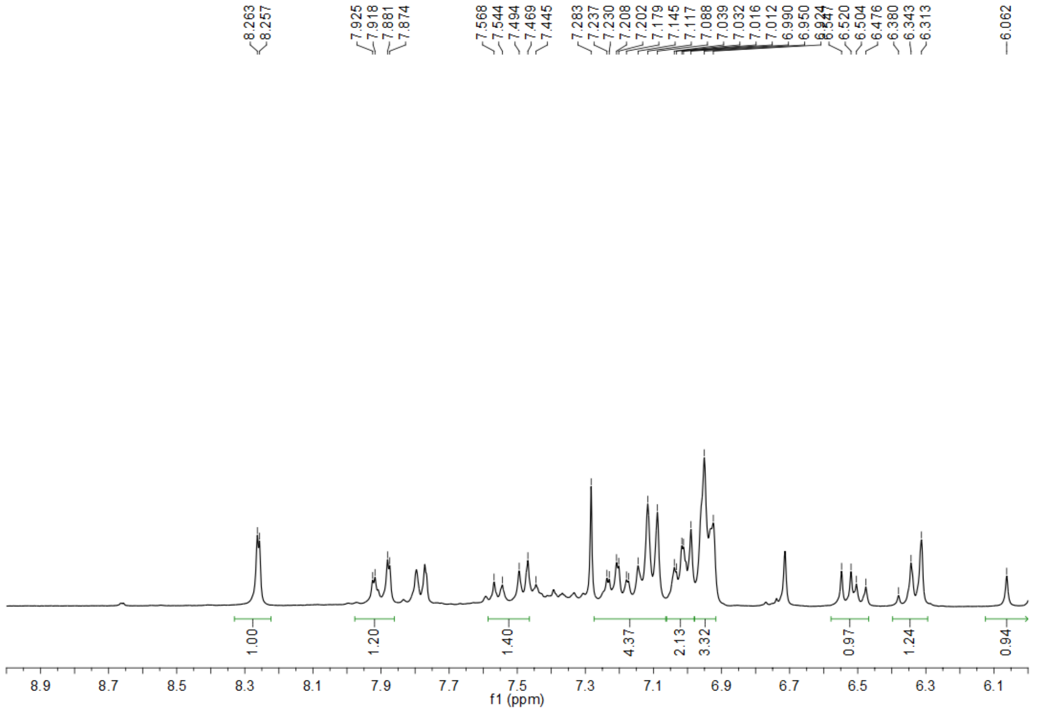

^{1}H NMR Spectrum of compound **3s (Spread)**

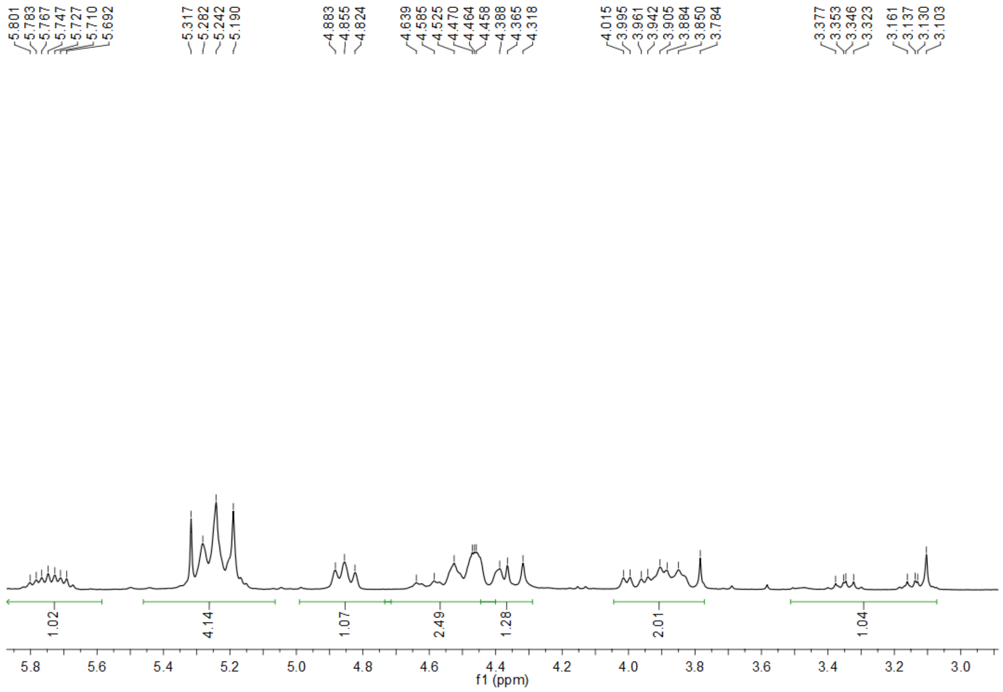

^{1}H NMR Spectrum of compound **3s (Spread)**

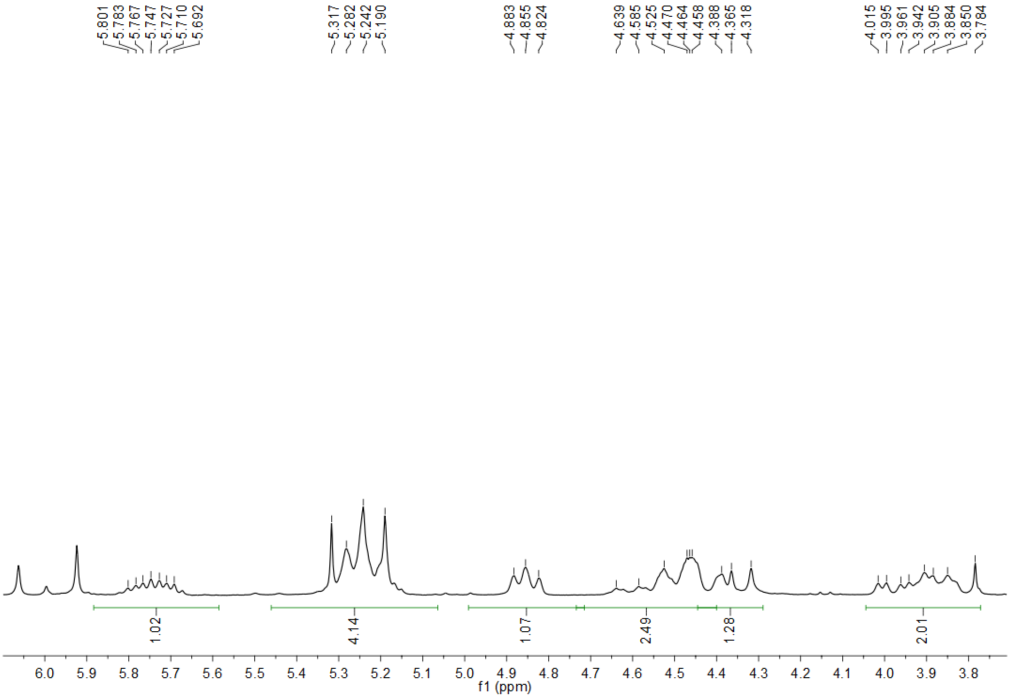

^{1}H NMR Spectrum of compound **3s (Spread)**

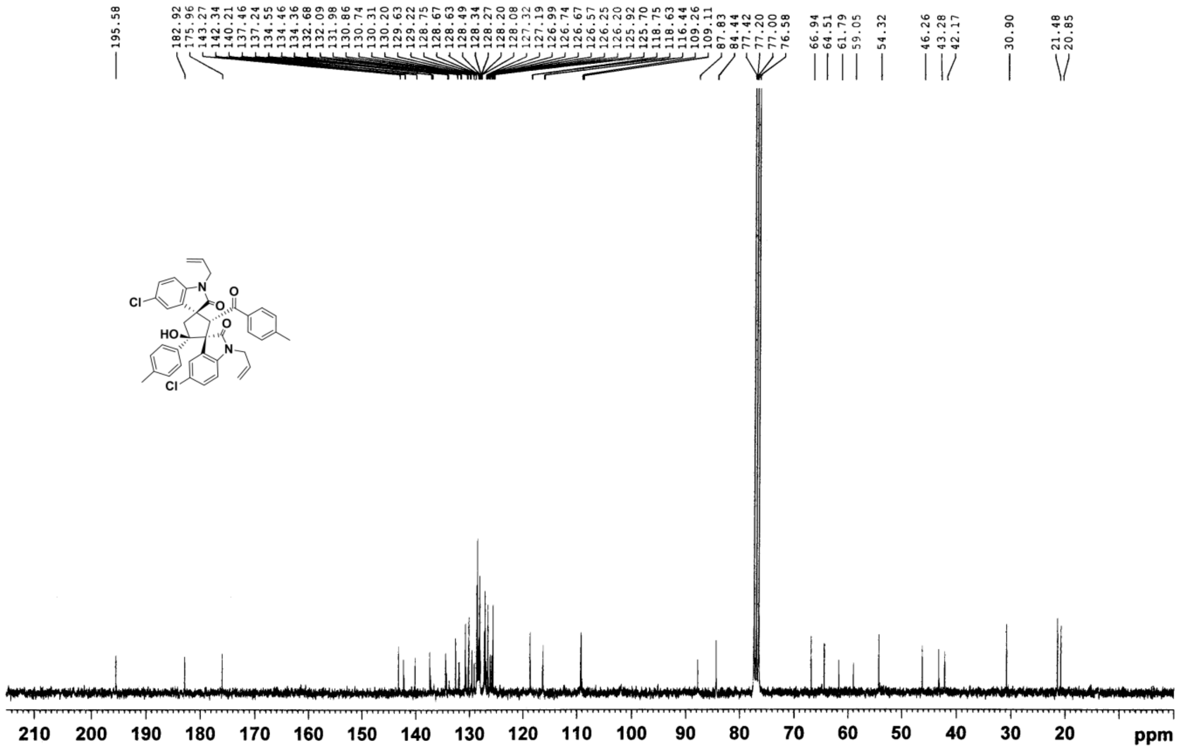

^{13}C NMR Spectrum of compound **3s**

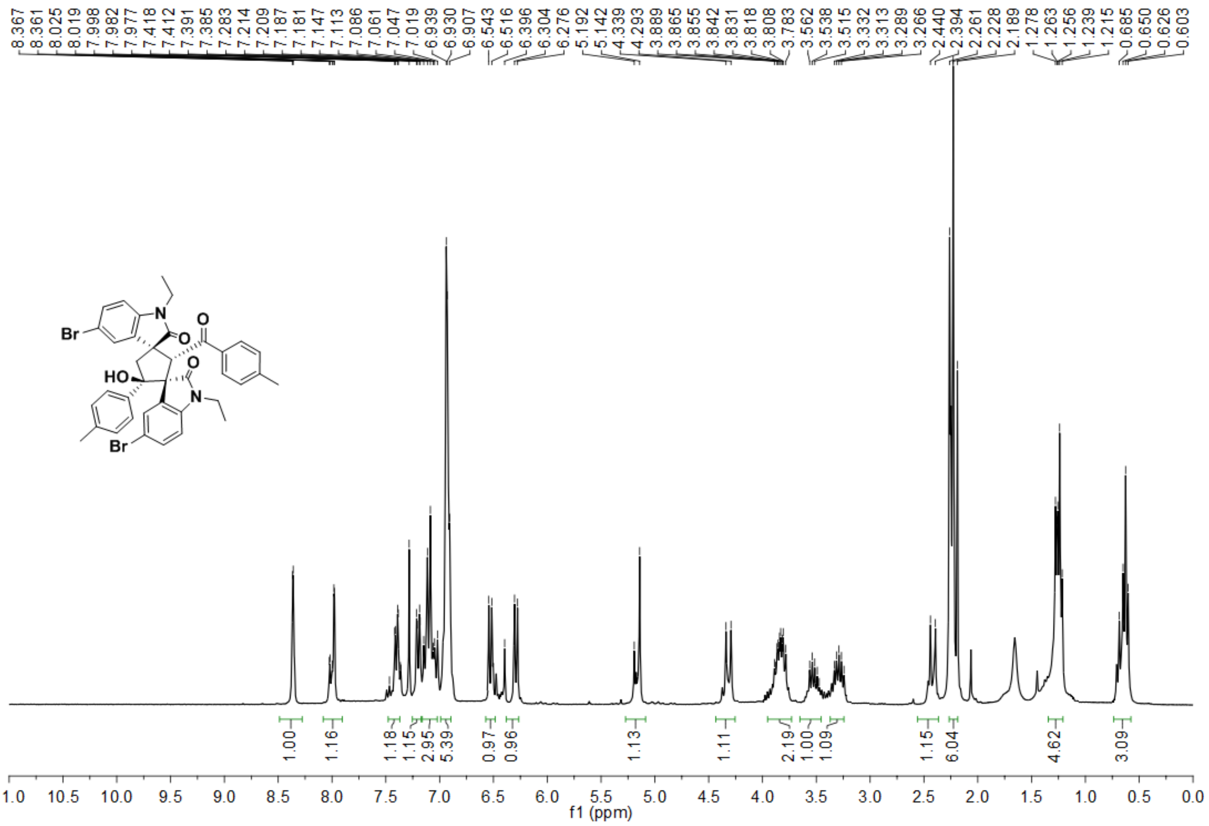

^{1}H NMR Spectrum of compound **3t**

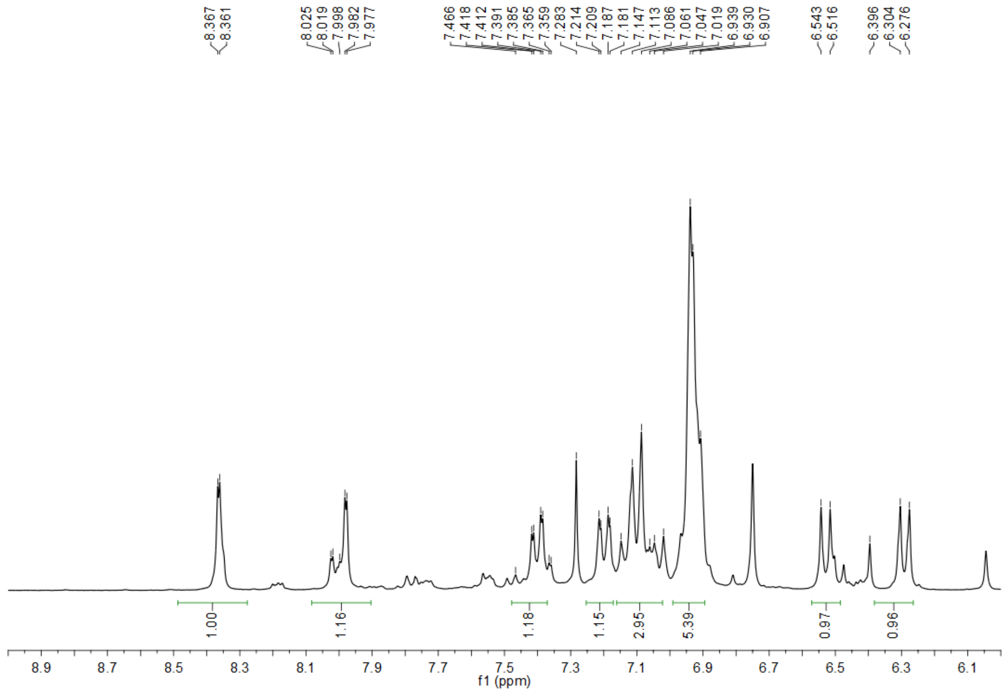

^{1}H NMR Spectrum of compound **3t (Spread)**

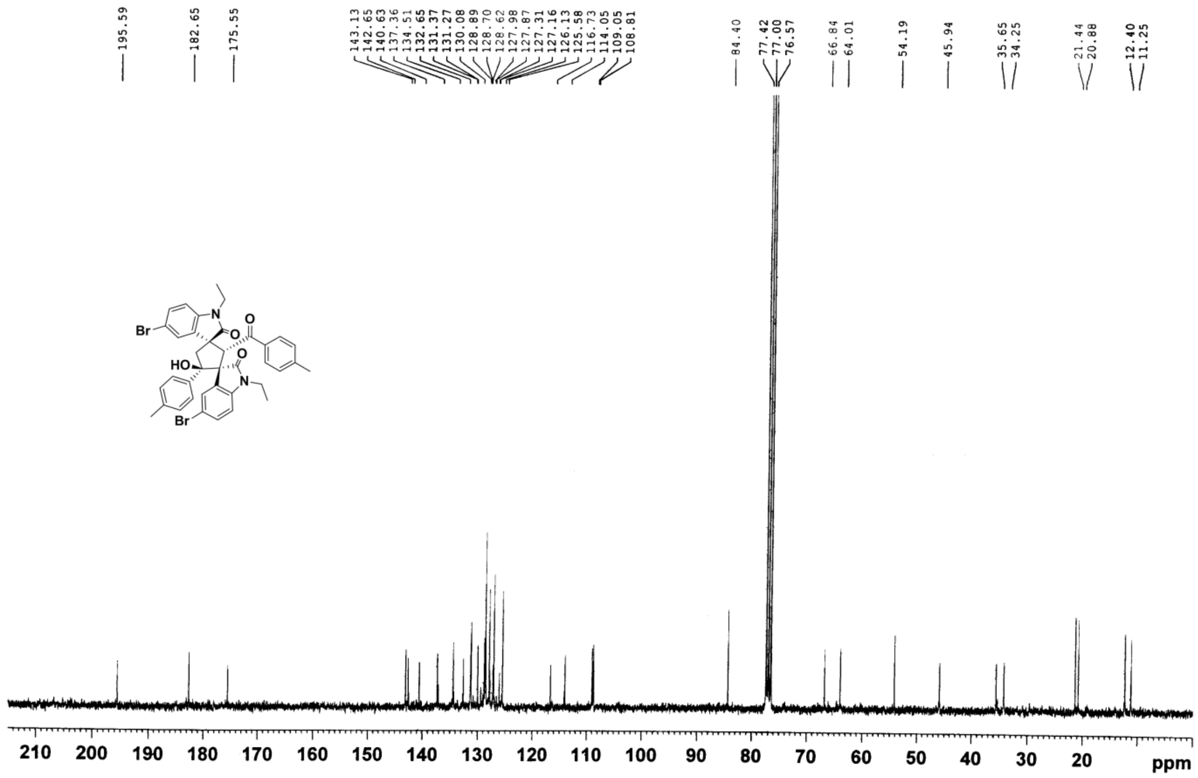

^{13}C NMR Spectrum of compound **3t**

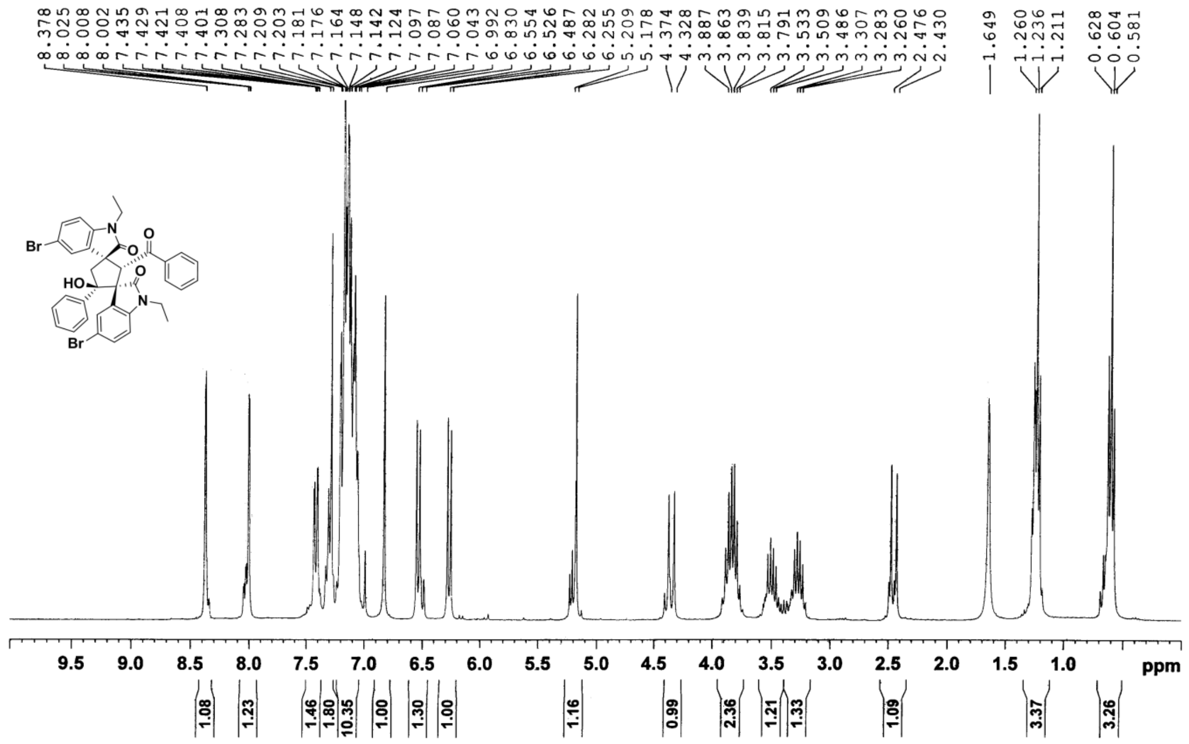

^{1}H NMR Spectrum of compound **3u**

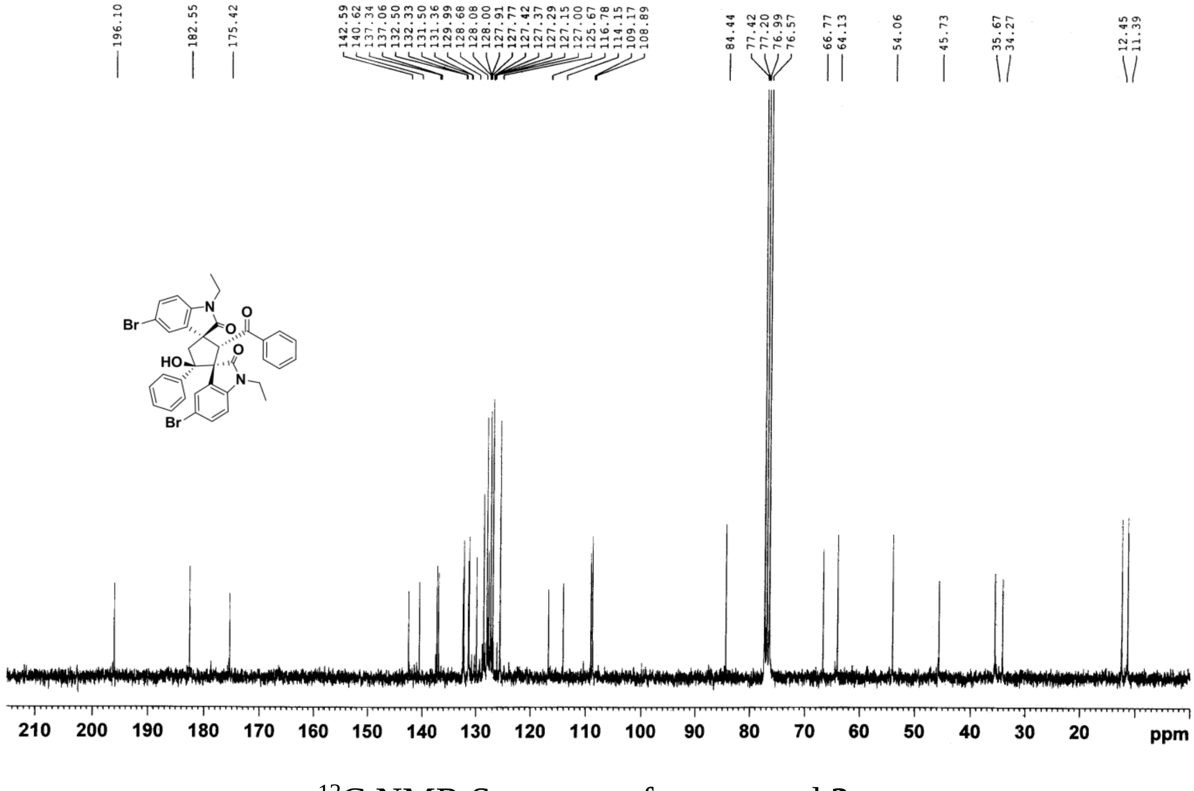

^{13}C NMR Spectrum of compound **3u**

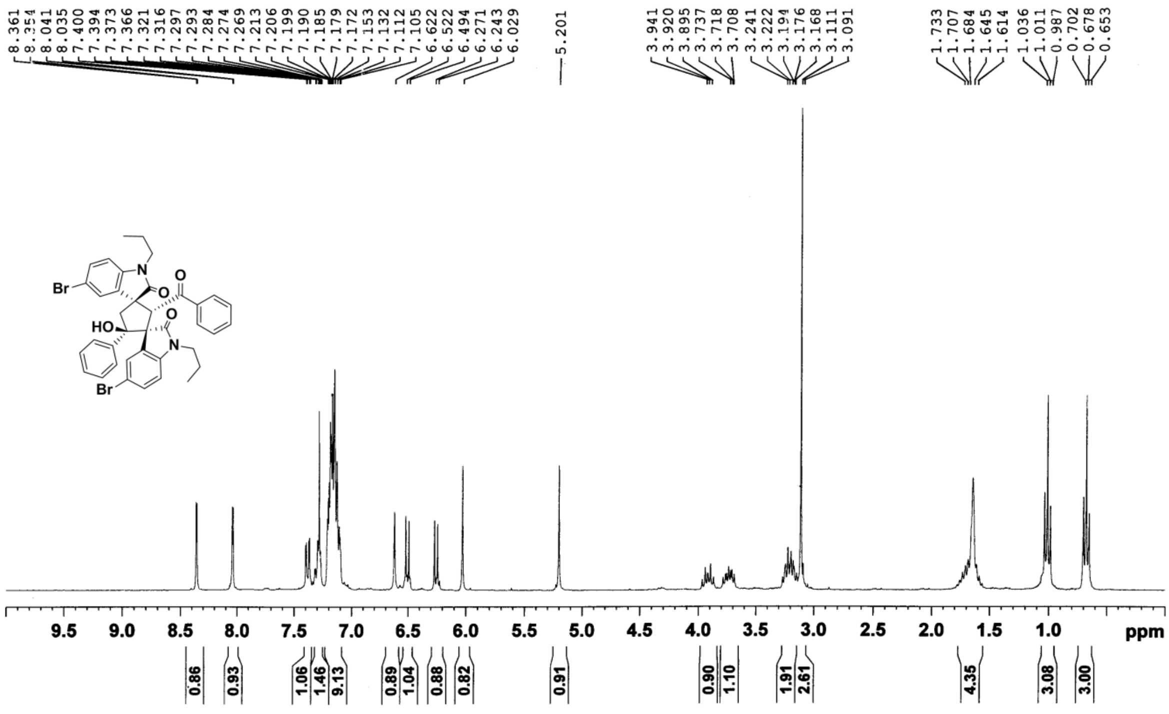

^{1}H NMR Spectrum of compound **3v**

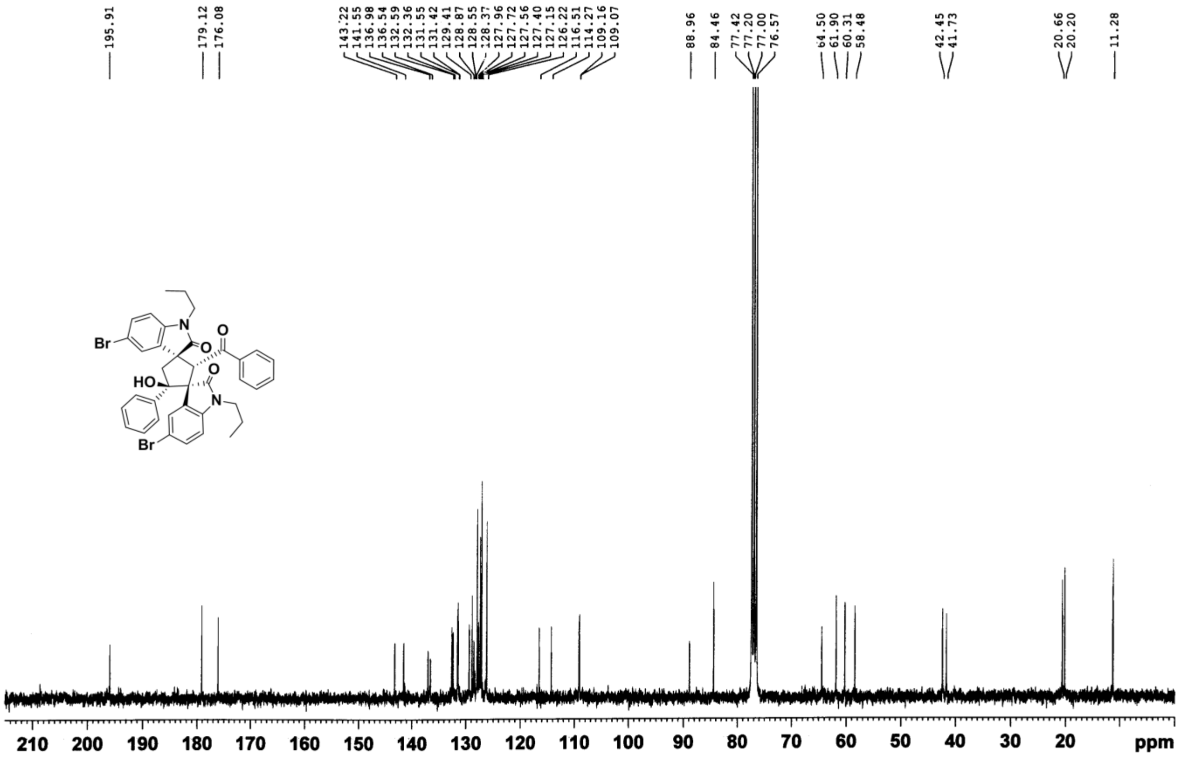

^{13}C NMR Spectrum of compound **3v**

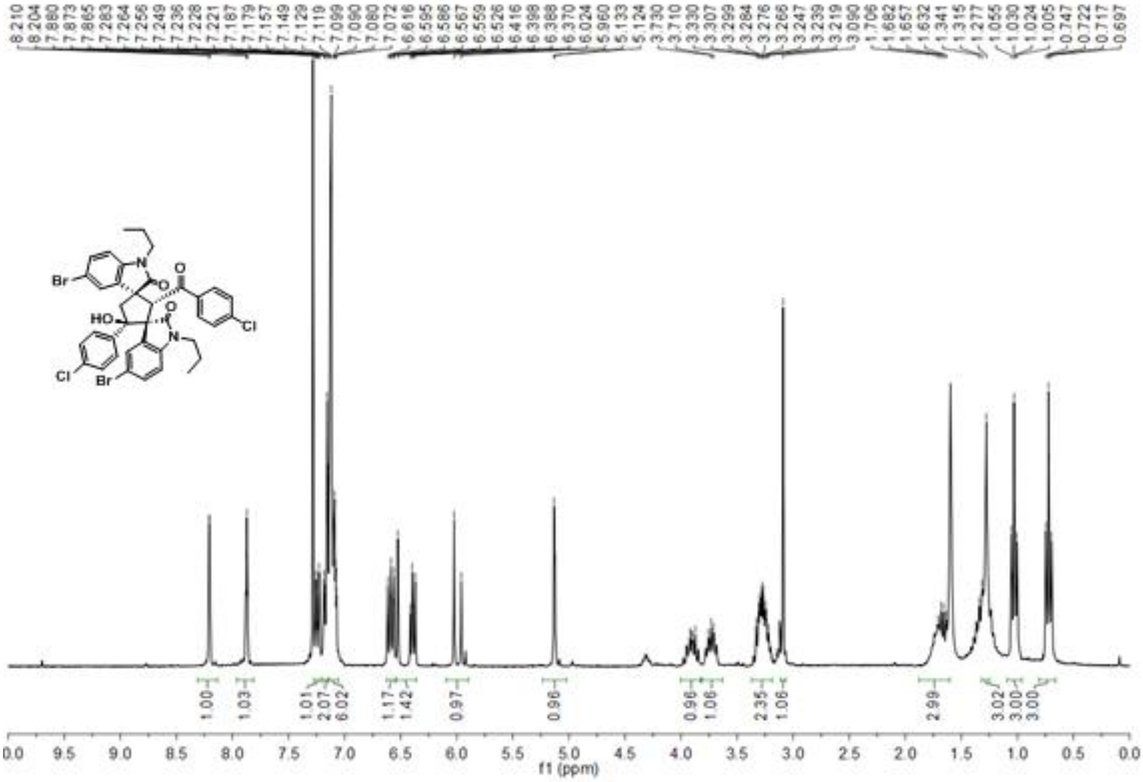

^{1}H NMR Spectrum of compound **3w**

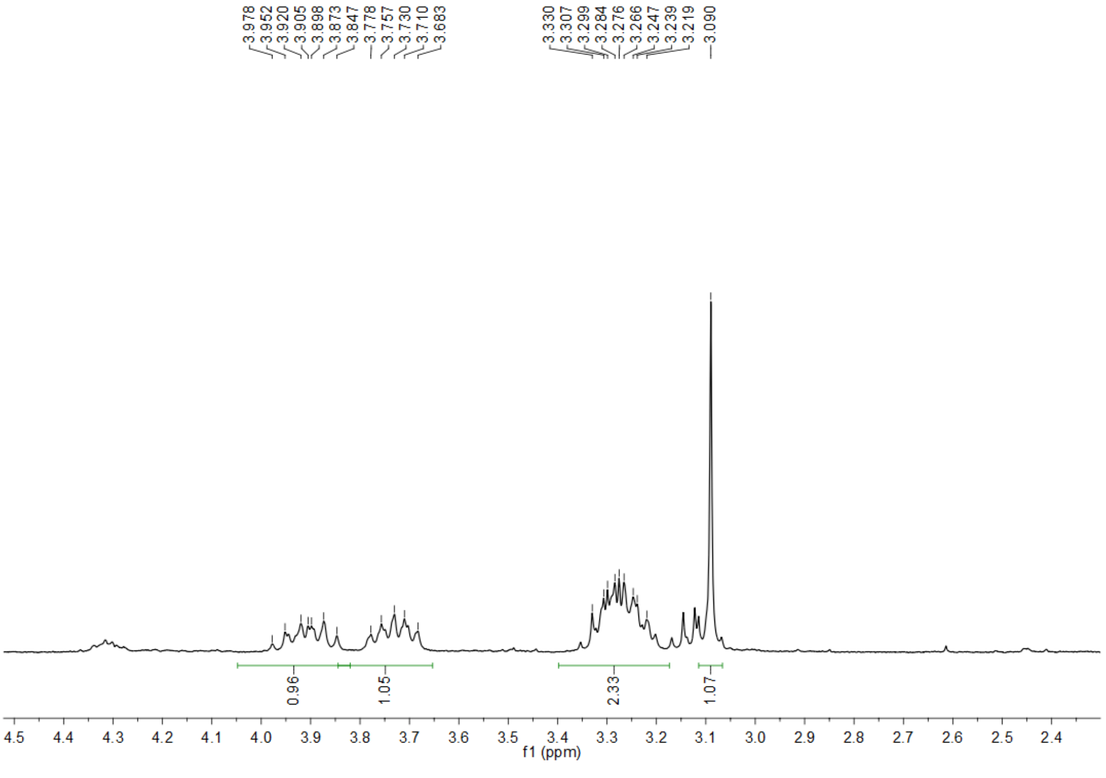

^{1}H NMR Spectrum of compound **3w (Spread)**

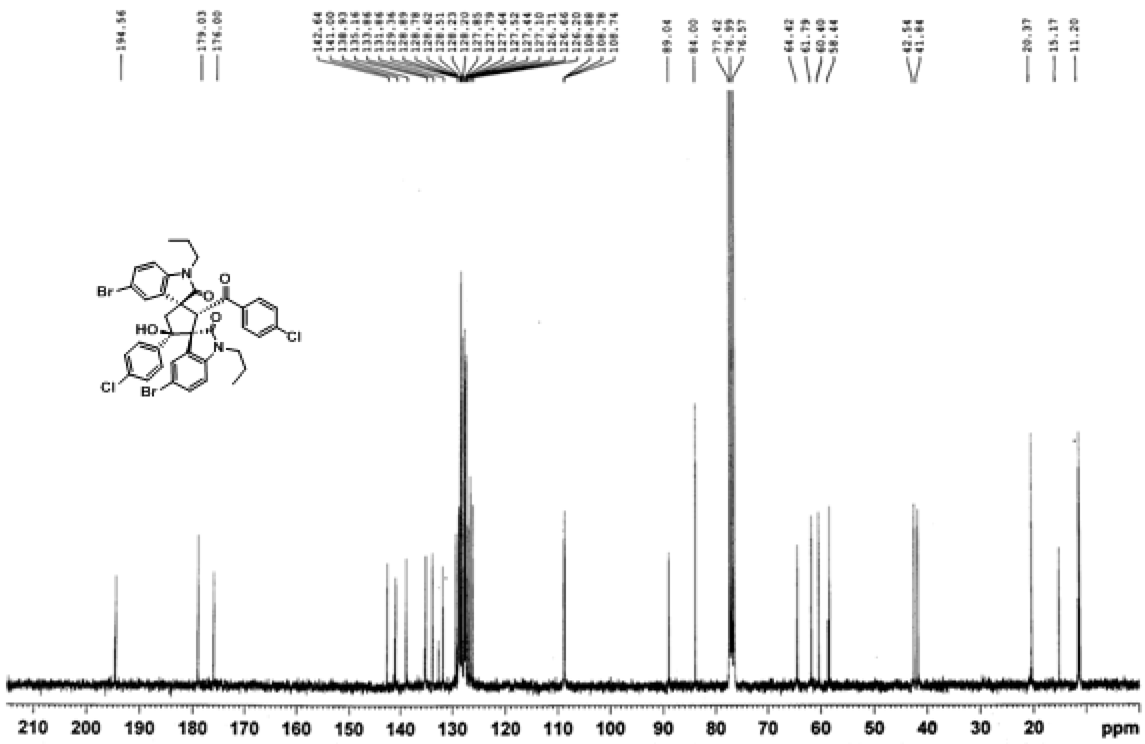

^{13}C NMR Spectrum of compound **3w**

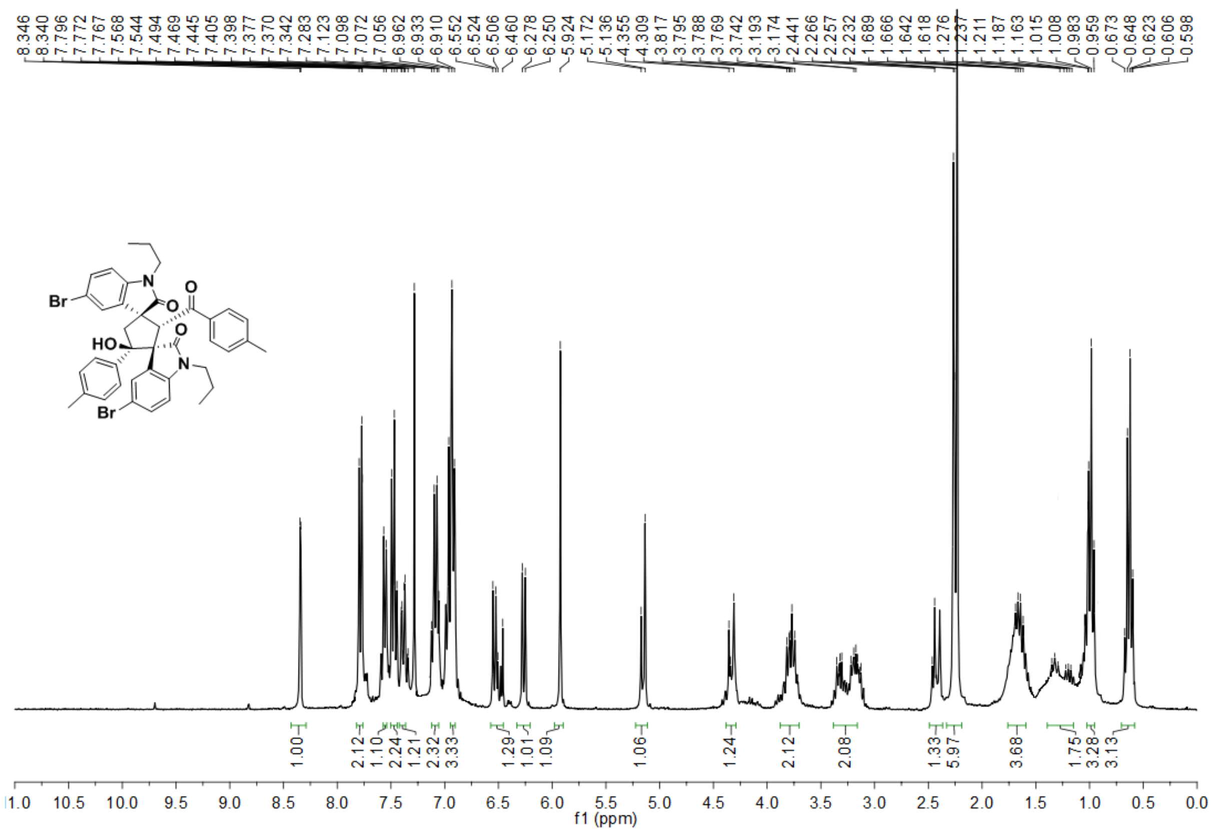

^{1}H NMR Spectrum of compound **3x**

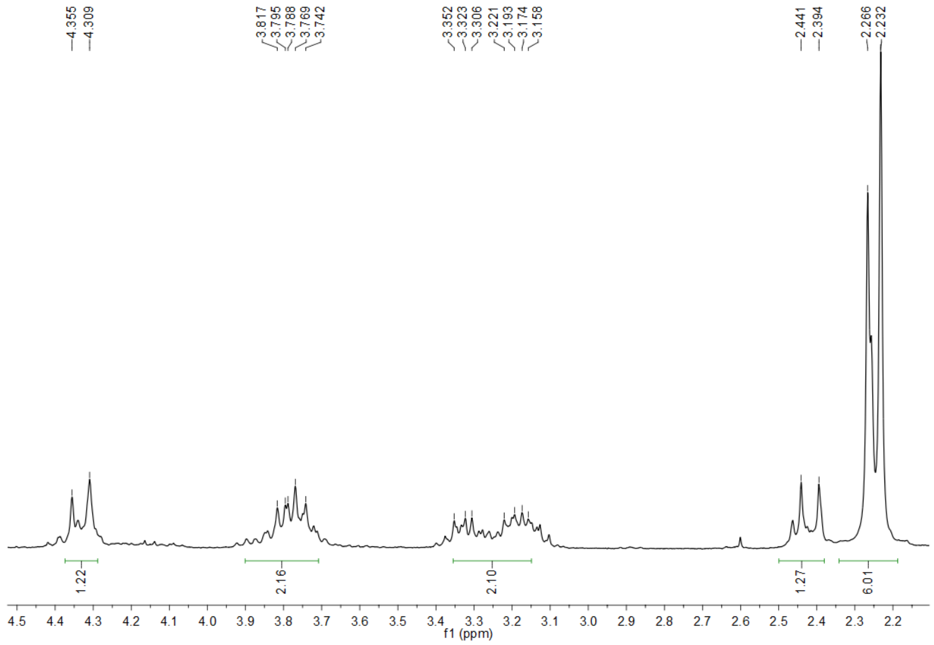

^{1}H NMR Spectrum of compound **3x (Spread)**

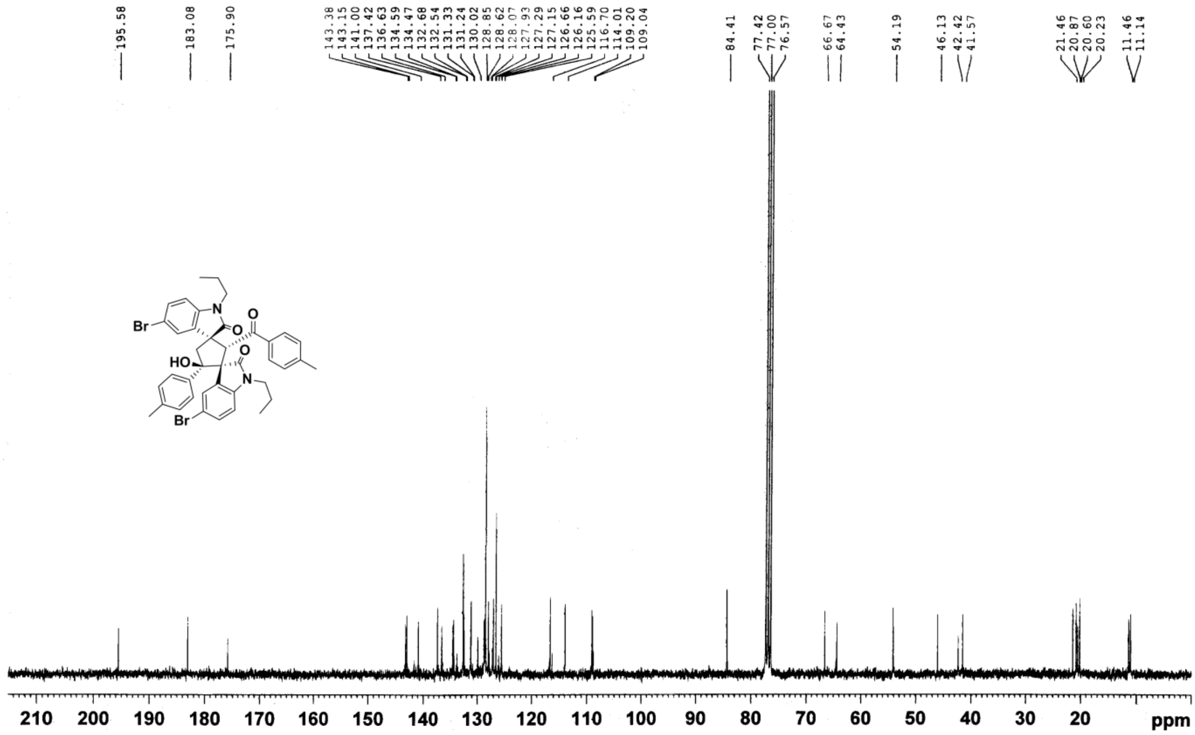

^{13}C NMR Spectrum of compound **3x**

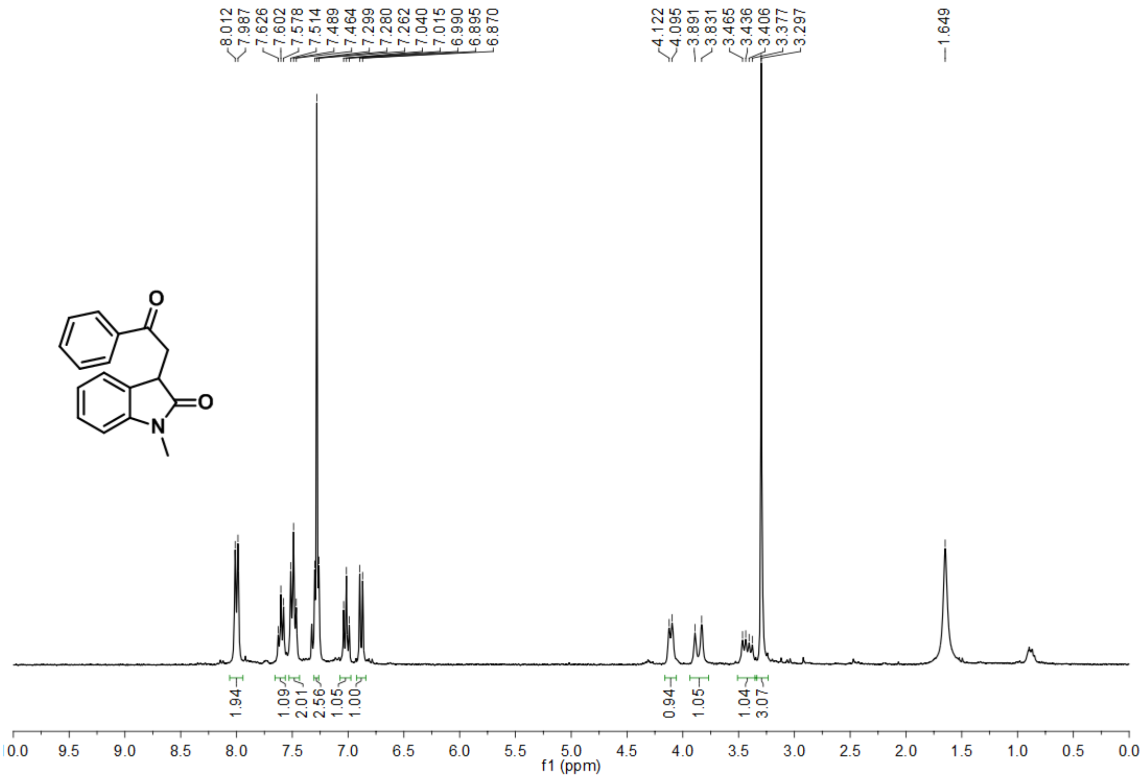

^{1}H NMR Spectrum of compound **4j**

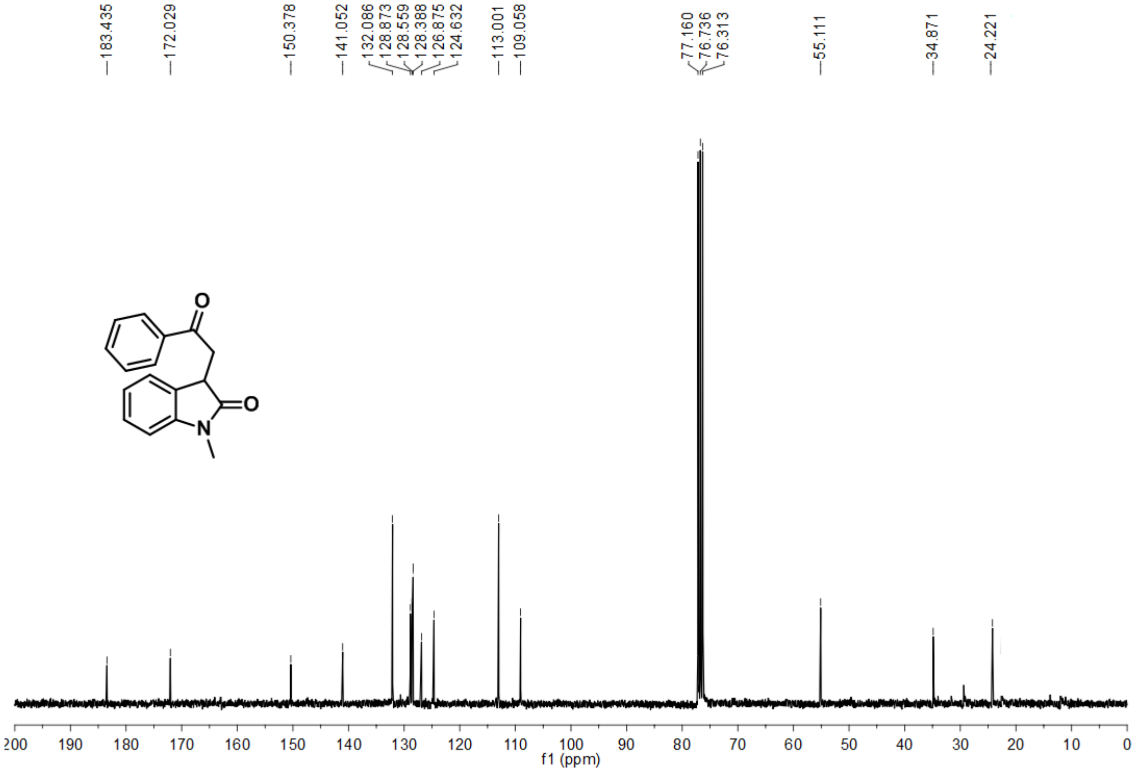

^{13}C NMR Spectrum of compound **4j**

<u>HPLC data of Compound **3p**</u>

Coloumn: GEMINI (100*4.6 mm) C18, 3 um : Dilunt: DMSO

Mobile Phase: A: HCOOH in water : B: CAN

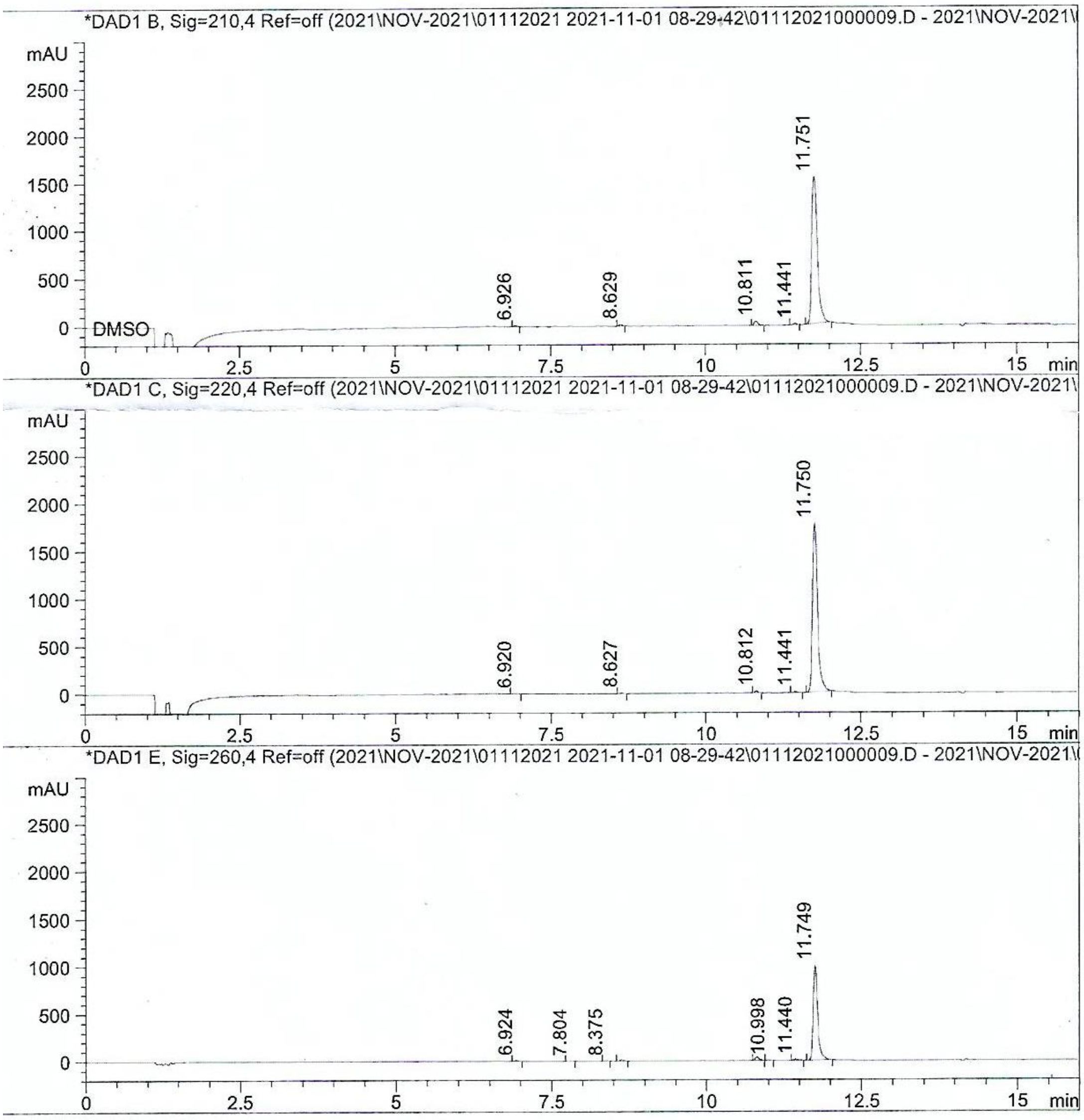

Signal 1: DAD1 B, Sig= 210,4 Ref= off

Peak	RT (min)	Area	Area %
1	6.93	36.03	0.35
2	8.63	58.88	0.58
3	10.81	162.46	1.60
4	11.44	51.23	0.50
5	11.75	9866.77	96.97

Signal 2: DAD1 C, Sig= 220,4 Ref= off

Peak	RT (min)	Area	Area %
1	6.92	26.66	0.26
2	8.63	39.03	0.38
3	10.81	76.40	0.74
4	11.44	56.60	0.55
5	11.75	10150.00	98.08

Signal 3: DAD1 E, Sig= 260,4 Ref= off

Peak	RT (min)	Area	Area %
1	6.92	27.79	0.52
2	7.80	7.07	0.13
3	8.38	8.48	0.16
4	8.63	46.08	0.87
5	10.81	124.70	2.35
6	11.00	5.03	0.09
7	11.44	44.12	0.83
8	11.75	5050.93	95.05

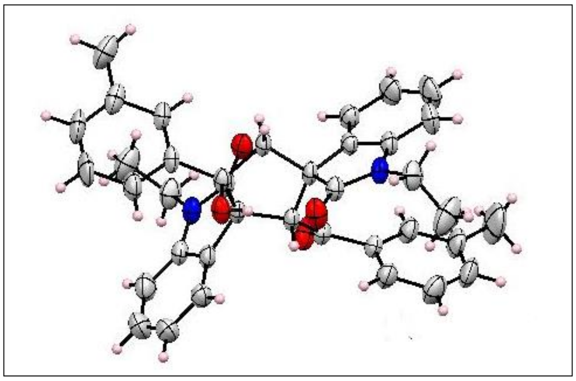

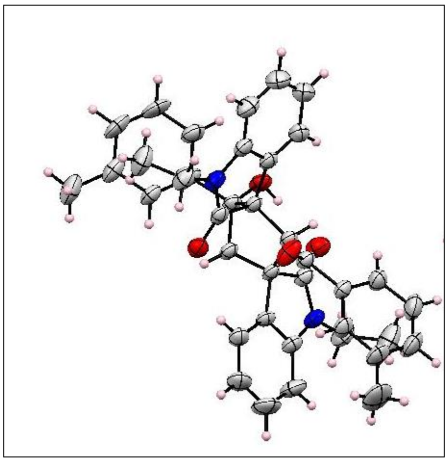

ORTEP diagram of product **3g (CCDC NO. 2072521)**

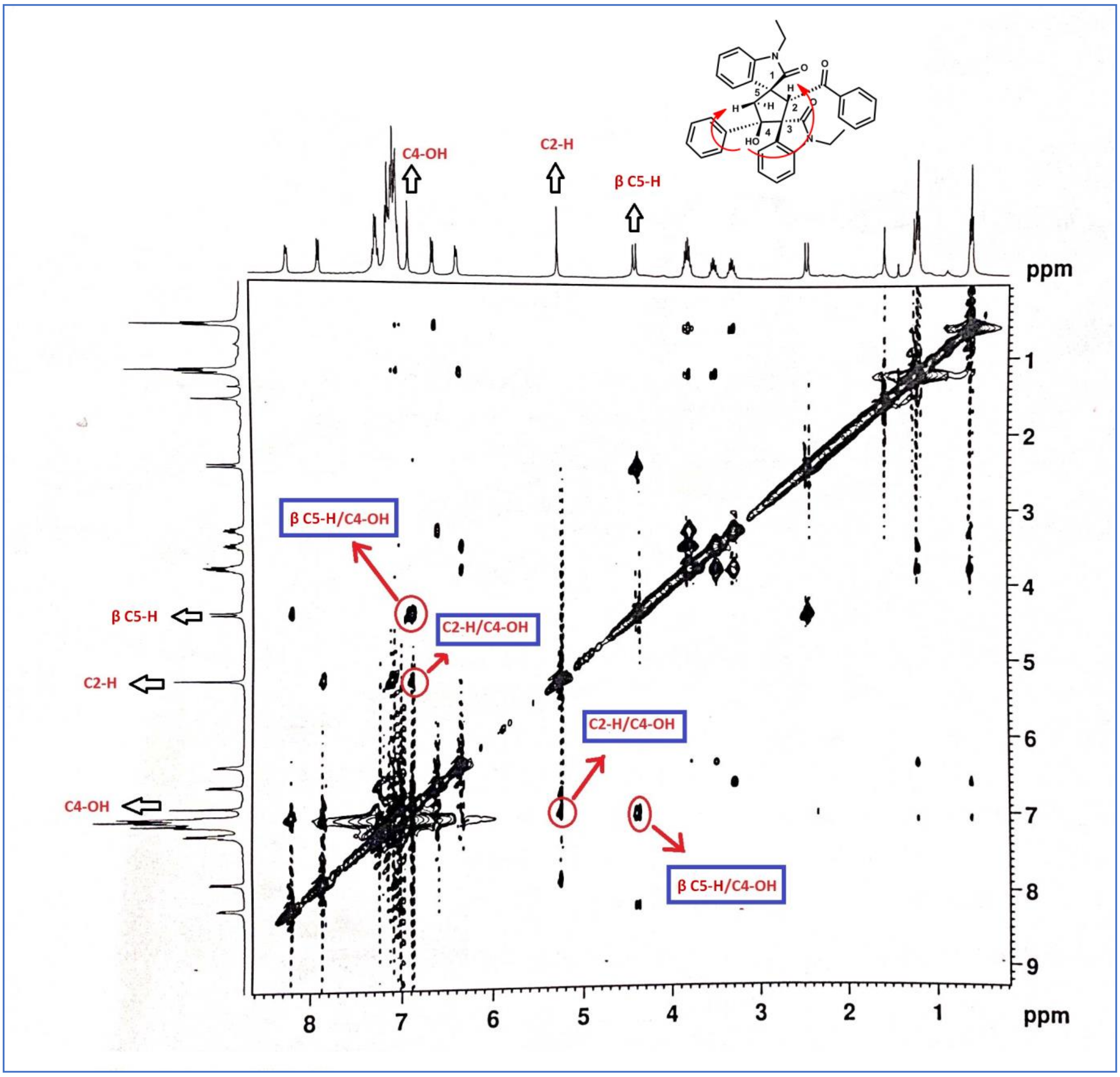

NOESY Spectra of Compound 3e

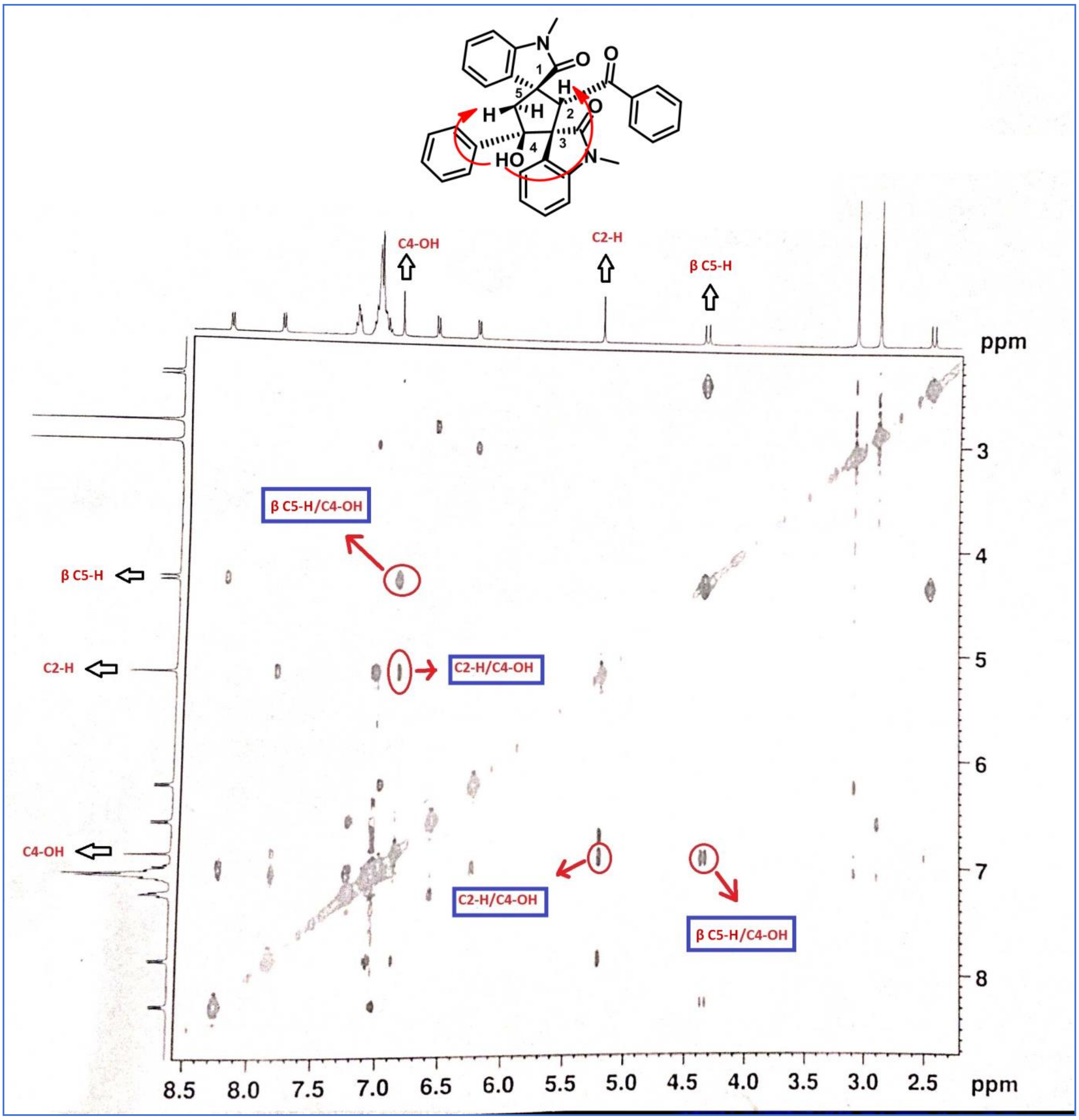

NOESY Spectra of Compound 3j

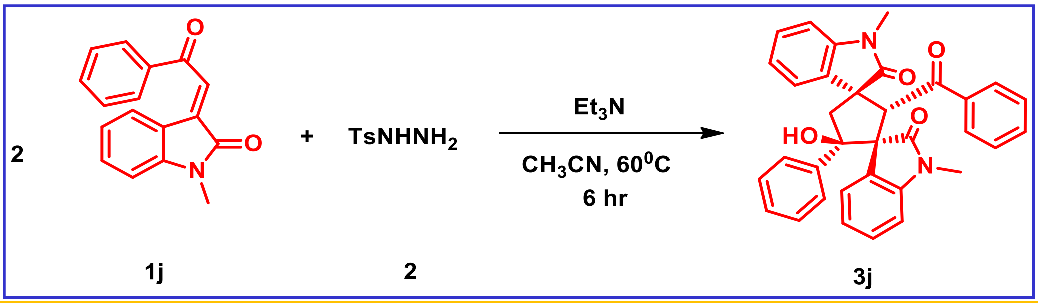

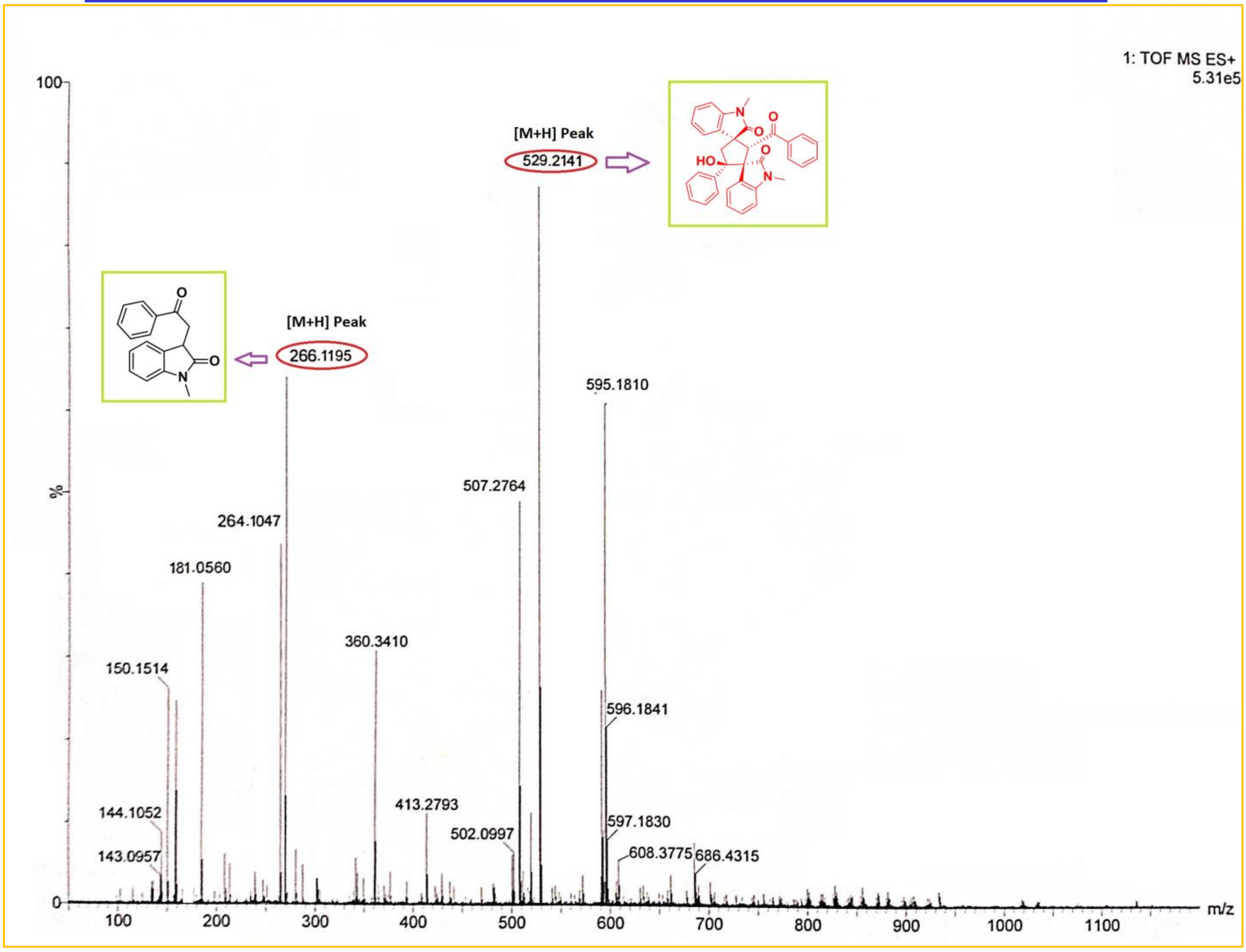

Crude HRMS spectra of reaction mixture

References

1. Pramanik, S.; Ray, S.; Maity, S.; Ghosh, P.; Mukhopadhyay, C. *Synthesis*.**2021**, *53(13)*, 2240-2252

2. Suman, K.; Ramanjaneyulua, M.; Thennarasu, S. *Org. Biomol. Chem*. **2017**, *15*, 1961.

3. Zhao, Y.; Yuan, Y.; Kong, L.; Zhang, F.; Li, Y. *Synthesis.* **2017**, *49(16)*, 3609-3618.
