# Supplementary material for: Tosylhydrazine-promoted self-conjugate reduction–Michael/aldol reaction of 3-phenacylideneoxindoles towards dispirocyclopentanebisoxindole derivatives
Source: Beilstein J Org Chem. 2022 Apr 27;18:469–78. doi: 10.3762/bjoc.18.49 (PMC9062653; doi:10.3762/bjoc.18.49)

BEILSTEIN JOURNAL OF ORGANIC CHEMISTRY

Supporting Information

for

Tosylhydrazine-promoted self-conjugate reduction–Michael/aldol reaction of 3-phenacylideneoxindoles towards dispirocyclopentanebisoxindole derivatives

Sayan Pramanik and Chhanda Mukhopadhyay

Beilstein J. Org. Chem. **2022,** *18,* 469–478. doi:10.3762/bjoc.18.49

Check-CIF-file for compound 3g

License and Terms: This is a supporting information file under the terms of the Creative Commons Attribution License (https://creativecommons.org/licenses/by/4.0). Please note that the reuse, redistribution and reproduction in particular requires that the author(s) and source are credited and that individual graphics may be subject to special legal provisions.
The license is subject to the *Beilstein Journal of Organic Chemistry* terms and conditions: (https://www.beilstein-journals.org/bjoc/terms)

checkCIF/PLATON report

Structure factors have been supplied for datablock(s) S_a

THIS REPORT IS FOR GUIDANCE ONLY. IF USED AS PART OF A REVIEW PROCEDURE FOR PUBLICATION, IT SHOULD NOT REPLACE THE EXPERTISE OF AN EXPERIENCED CRYSTALLOGRAPHIC REFEREE.

No syntax errors found. CIF dictionary Interpreting this report

Datablock: S_a

```
Bond precision:  C-C = 0.0035 A          Wavelength=0.71073

Cell:           a=13.790(4)       b=15.024(5)       c=15.179(5)
                alpha=90.003(4)   beta=102.176(4)   gamma=90.014(4)
Temperature:    296 K

                 Calculated                Reported
Volume           3074.1(17)                3074.0(16)
Space group      P -1                      P -1
Hall group       -P 1                      -P 1
Moiety formula   C38 H36 N2 O4             ?
Sum formula      C38 H36 N2 O4             C76 H70 N4 O8
Mr               584.69                    1167.36
Dx,g cm-3        1.263                     1.261
Z                4                         2
Mu (mm-1)        0.082                     0.082
F000             1240.0                    1236.0
F000'            1240.54
h,k,lmax         16,17,17                  15,17,17
Nref             10307                     9977
Tmin,Tmax        0.994,0.998               0.763,0.846
Tmin'            0.990

Correction method= # Reported T Limits: Tmin=0.763 Tmax=0.846
AbsCorr = MULTI-SCAN

Data completeness= 0.968          Theta(max)= 24.563

R(reflections)= 0.0626( 7141)     wR2(reflections)= 0.1769( 9977)

S = 0.942                  Npar= 803
```

The following ALERTS were generated. Each ALERT has the format
test-name_ALERT_alert-type_alert-level.
Click on the hyperlinks for more details of the test.

Alert level B

```
PLAT112_ALERT_2_B ADDSYM Detects New (Pseudo) Symm. Elem         a        100 %Fit
PLAT113_ALERT_2_B ADDSYM Suggests Possible Pseudo/New  Space Group      P21/c Check
```

Alert level C

```
ABSTY02_ALERT_1_C  An _exptl_absorpt_correction_type has been given without
            a literature citation. This should be contained in the
            _exptl_absorpt_process_details field.
            Absorption correction given as multi-scan
RINTA01_ALERT_3_C  The value of Rint is greater than 0.12
            Rint given   0.147
THETM01_ALERT_3_C  The value of sine(theta_max)/wavelength is less than 0.590
            Calculated sin(theta_max)/wavelength =    0.5849
PLAT029_ALERT_3_C _diffrn_measured_fraction_theta_full value Low .      0.968 Why?
PLAT041_ALERT_1_C Calc. and Reported SumFormula    Strings  Differ     Please Check
PLAT043_ALERT_1_C Calculated and Reported Mol. Weight Differ by ..       1.01 Check
PLAT068_ALERT_1_C Reported F000 Differs from Calcd (or Missing)...     Please Check
PLAT220_ALERT_2_C NonSolvent   Resd 1  C   Ueq(max)/Ueq(min) Range        3.4 Ratio
PLAT220_ALERT_2_C NonSolvent   Resd 2  C   Ueq(max)/Ueq(min) Range        3.3 Ratio
PLAT222_ALERT_3_C NonSolvent Resd 1  H   Uiso(max)/Uiso(min) Range        4.3 Ratio
PLAT222_ALERT_3_C NonSolvent Resd 2  H   Uiso(max)/Uiso(min) Range        4.1 Ratio
PLAT250_ALERT_2_C Large U3/U1 Ratio for Average U(i,j) Tensor ....        2.2 Note
PLAT905_ALERT_3_C Negative K value in the Analysis of Variance ...     -1.590 Report
PLAT911_ALERT_3_C Missing FCF Refl Between Thmin & STh/L=    0.585        312 Report
```

Alert level G

```
FORMU01_ALERT_2_G  There is a discrepancy between the atom counts in the
            _chemical_formula_sum and the formula from the _atom_site* data.
            Atom count from _chemical_formula_sum:C76 H70 N4 O8
            Atom count from the _atom_site data:  C76 H72 N4 O8
CELLZ01_ALERT_1_G Difference between formula and atom_site contents detected.
CELLZ01_ALERT_1_G ALERT: Large difference may be due to a
            symmetry error - see SYMMG tests
           From the CIF: _cell_formula_units_Z    2
           From the CIF: _chemical_formula_sum  C76 H70 N4 O8
           TEST: Compare cell contents of formula and atom_site data

           atom    Z*formula  cif sites diff
           C        152.00    152.00    0.00
           H        140.00    144.00   -4.00
           N          8.00      8.00    0.00
           O         16.00     16.00    0.00
PLAT007_ALERT_5_G Number of Unrefined Donor-H Atoms ..............          2 Report
PLAT020_ALERT_3_G The Value of Rint is Greater Than 0.12 .........      0.147 Report
PLAT045_ALERT_1_G Calculated and Reported Z Differ by a Factor ...       2.00 Check
PLAT154_ALERT_1_G The s.u.'s on the Cell Angles are Equal ..(Note)      0.004 Degree
PLAT793_ALERT_4_G Model has Chirality at C5          (Centro SPGR)          R Verify
PLAT793_ALERT_4_G Model has Chirality at C6          (Centro SPGR)          R Verify
PLAT793_ALERT_4_G Model has Chirality at C7          (Centro SPGR)          R Verify
PLAT793_ALERT_4_G Model has Chirality at C17         (Centro SPGR)          R Verify
PLAT793_ALERT_4_G Model has Chirality at C43         (Centro SPGR)          R Verify
PLAT793_ALERT_4_G Model has Chirality at C44         (Centro SPGR)          R Verify
PLAT793_ALERT_4_G Model has Chirality at C45         (Centro SPGR)          R Verify
PLAT793_ALERT_4_G Model has Chirality at C53         (Centro SPGR)          R Verify
PLAT883_ALERT_1_G No Info/Value for _atom_sites_solution_primary .     Please Do !
PLAT909_ALERT_3_G Percentage of I>2sig(I) Data at Theta(Max) Still        49% Note
PLAT941_ALERT_3_G Average HKL Measurement Multiplicity ...........        2.1 Low
PLAT965_ALERT_2_G The SHELXL WEIGHT Optimisation has not Converged     Please Check
PLAT978_ALERT_2_G Number C-C Bonds with Positive Residual Density.          3 Info
```

```
PLAT992_ALERT_5_G Repd & Actual _reflns_number_gt Values Differ by          2 Check
```

```
   0 ALERT level A = Most likely a serious problem - resolve or explain
   2 ALERT level B = A potentially serious problem, consider carefully
  14 ALERT level C = Check. Ensure it is not caused by an omission or oversight
  21 ALERT level G = General information/check it is not something unexpected

   9 ALERT type 1 CIF construction/syntax error, inconsistent or missing data
   8 ALERT type 2 Indicator that the structure model may be wrong or deficient
  10 ALERT type 3 Indicator that the structure quality may be low
   8 ALERT type 4 Improvement, methodology, query or suggestion
   2 ALERT type 5 Informative message, check
```

Validation response form

Please find below a validation response form (VRF) that can be filled in and pasted into your CIF.

```
# start Validation Reply Form
_vrf_ABSTY02_S_a
;
PROBLEM: An _exptl_absorpt_correction_type has been given without
RESPONSE: ...
;
_vrf_RINTA01_S_a
;
PROBLEM: The value of Rint is greater than 0.12
RESPONSE: ...
;
_vrf_THETM01_S_a
;
PROBLEM: The value of sine(theta_max)/wavelength is less than 0.590
RESPONSE: ...
;
_vrf_PLAT112_S_a
;
PROBLEM: ADDSYM Detects New (Pseudo) Symm. Elem         a        100 %Fit
RESPONSE: ...
;
_vrf_PLAT113_S_a
;
PROBLEM: ADDSYM Suggests Possible Pseudo/New  Space Group      P21/c Check
RESPONSE: ...
;
_vrf_PLAT029_S_a
;
PROBLEM: _diffrn_measured_fraction_theta_full value Low .      0.968 Why?
RESPONSE: ...
;
_vrf_PLAT041_S_a
;
PROBLEM: Calc. and Reported SumFormula    Strings  Differ     Please Check
RESPONSE: ...
;
_vrf_PLAT043_S_a
;
PROBLEM: Calculated and Reported Mol. Weight Differ by ..       1.01 Check
RESPONSE: ...
;
_vrf_PLAT068_S_a
```

```
;
PROBLEM: Reported F000 Differs from Calcd (or Missing)...     Please Check
RESPONSE: ...
;
_vrf_PLAT220_S_a
;
PROBLEM: NonSolvent   Resd 1  C   Ueq(max)/Ueq(min) Range        3.4 Ratio
RESPONSE: ...
;
_vrf_PLAT222_S_a
;
PROBLEM: NonSolvent Resd 1  H   Uiso(max)/Uiso(min) Range        4.3 Ratio
RESPONSE: ...
;
_vrf_PLAT250_S_a
;
PROBLEM: Large U3/U1 Ratio for Average U(i,j) Tensor ....        2.2 Note
RESPONSE: ...
;
_vrf_PLAT905_S_a
;
PROBLEM: Negative K value in the Analysis of Variance ...     -1.590 Report
RESPONSE: ...
;
_vrf_PLAT911_S_a
;
PROBLEM: Missing FCF Refl Between Thmin & STh/L=    0.585        312 Report
RESPONSE: ...
;
# end Validation Reply Form
```

It is advisable to attempt to resolve as many as possible of the alerts in all categories. Often the minor alerts point to easily fixed oversights, errors and omissions in your CIF or refinement strategy, so attention to these fine details can be worthwhile. In order to resolve some of the more serious problems it may be necessary to carry out additional measurements or structure refinements. However, the purpose of your study may justify the reported deviations and the more serious of these should normally be commented upon in the discussion or experimental section of a paper or in the "special_details" fields of the CIF. checkCIF was carefully designed to identify outliers and unusual parameters, but every test has its limitations and alerts that are not important in a particular case may appear. Conversely, the absence of alerts does not guarantee there are no aspects of the results needing attention. It is up to the individual to critically assess their own results and, if necessary, seek expert advice.

Publication of your CIF in IUCr journals

A basic structural check has been run on your CIF. These basic checks will be run on all CIFs submitted for publication in IUCr journals (*Acta Crystallographica*, *Journal of Applied Crystallography*, *Journal of Synchrotron Radiation*); however, if you intend to submit to *Acta Crystallographica Section C* or *E* or *IUCrData*, you should make sure that full publication checks are run on the final version of your CIF prior to submission.

Publication of your CIF in other journals

Please refer to the *Notes for Authors* of the relevant journal for any special instructions relating to CIF submission.

PLATON version of 05/12/2020; check.def file version of 05/12/2020

Datablock S_a - ellipsoid plot

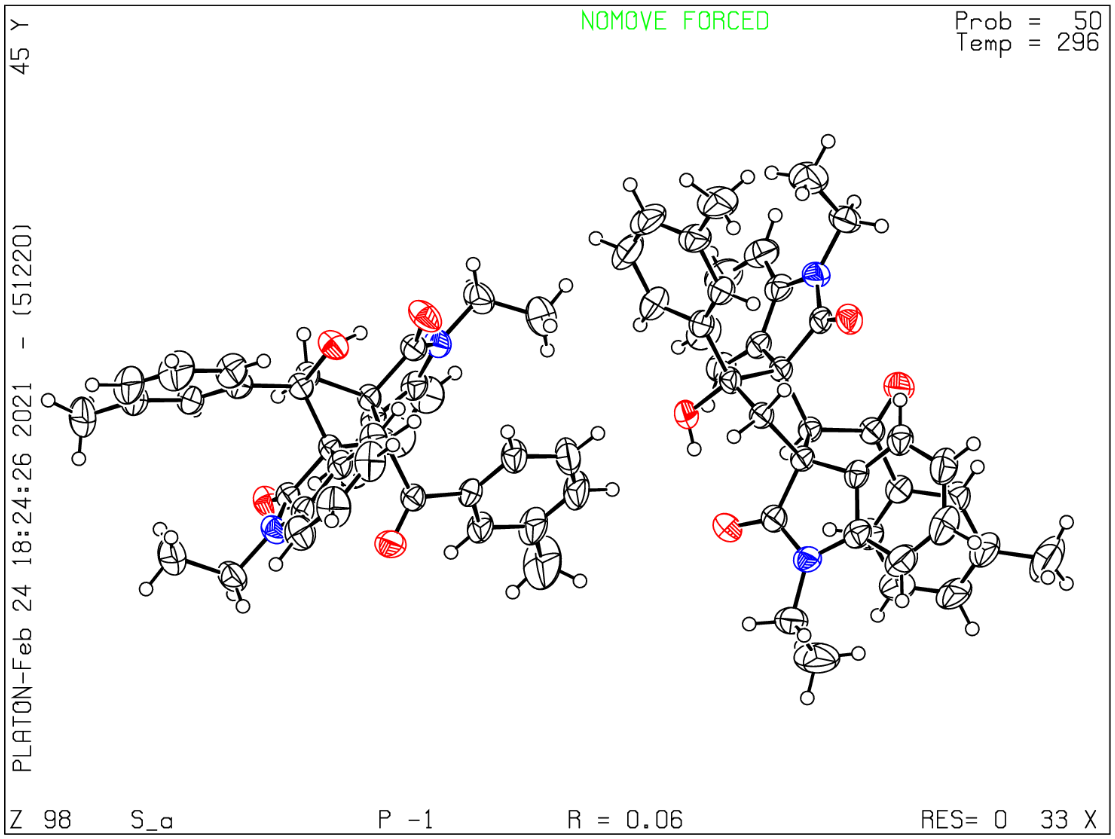

Supplement: File 3 — Check-CIF-file for compound 3g. [file Beilstein_J_Org_Chem-18-469-s003.pdf]
